# Accurate Promoter and Enhancer Identification in 127 ENCODE and Roadmap Epigenomics Cell Types and Tissues by GenoSTAN

**DOI:** 10.1371/journal.pone.0169249

**Published:** 2017-01-05

**Authors:** Benedikt Zacher, Margaux Michel, Björn Schwalb, Patrick Cramer, Achim Tresch, Julien Gagneur

**Affiliations:** 1 Gene Center and Department of Biochemistry, Center for Integrated Protein Science CIPSM, Ludwig-Maximilians-Universität Munich, Germany; 2 Department of Biology, University of Cologne, Cologne, Germany; 3 Max Planck Institute for Plant Breeding Research, Cologne, Germany; 4 Department of Molecular Biology, Max Planck Institute for Biophysical Chemistry, Göttingen, Germany; Università degli Studi di Milano, ITALY

## Abstract

Accurate maps of promoters and enhancers are required for understanding transcriptional regulation. Promoters and enhancers are usually mapped by integration of chromatin assays charting histone modifications, DNA accessibility, and transcription factor binding. However, current algorithms are limited by unrealistic data distribution assumptions. Here we propose GenoSTAN (Genomic STate ANnotation), a hidden Markov model overcoming these limitations. We map promoters and enhancers for 127 cell types and tissues from the ENCODE and Roadmap Epigenomics projects, today’s largest compendium of chromatin assays. Extensive benchmarks demonstrate that GenoSTAN generally identifies promoters and enhancers with significantly higher accuracy than previous methods. Moreover, GenoSTAN-derived promoters and enhancers showed significantly higher enrichment of complex trait-associated genetic variants than current annotations. Altogether, GenoSTAN provides an easy-to-use tool to define promoters and enhancers in any system, and our annotation of human transcriptional cis-regulatory elements constitutes a rich resource for future research in biology and medicine.

## Introduction

Transcription is tightly regulated by cis-regulatory DNA elements known as promoters and enhancers. These elements control development, cell fate and may lead to disease if impaired. A promoter is functionally defined as a region that regulates transcription of a gene, located upstream and in close proximity to the transcription start sites (TSSs) [1]. In contrast, an enhancer was originally functionally defined as a DNA element that can increase expression of a gene over a long distance in an orientation-independent fashion relative to the gene [2]. The functional definition of enhancers and promoters leads to practical difficulties for their genome-wide identification because the direct measurement of the regulatory activity of genomic regions is hard, with current approaches leading to contradicting results [3–5].

Since the direct measurement of cis-regulatory activity is challenging, a biochemical characterization of the chromatin at these elements based on histone modifications, DNA accessibility, and transcription factor binding has been proposed [6–10]. This approach leverages extensive genome-wide datasets of chromatin-immunoprecipitation followed by sequencing (ChIP-Seq) of transcription factors (TFs), histone modifications, or Cap analysis gene expression (CAGE) that have been generated by collaborative projects such as ENCODE [11, 12], NIH Roadmap Epigenomics [13], BLUEPRINT [14] and FANTOM [15, 16].

In this context, the computational approaches employed to classify genomic regions as enhancers or promoters play a decisive role [6, 10]. As the experimental data are heterogeneous, we generally refer to them as tracks. Several studies used supervised learning techniques to predict enhancers based on tracks such as histone modifications or P300 binding (e.g. [17–20]). However, a training set of validated enhancers is needed in this case, which is hard to define since only few enhancers have been validated experimentally so far and these might be biased towards specific enhancer subclasses. Alternatively, unsupervised learning algorithms were developed to identify promoters and enhancers from combinations of histone marks and protein-DNA interactions alone [8, 9, 11, 13, 21–24]. These unsupervised methods perform genome segmentation, i.e. they model the genome as a succession of segments in different chromatin states defined by characteristic combinations of histone marks and protein-DNA interactions found recurrently throughout the genome. All popular genome segmentations are based on hidden Markov models [25], or their generalized form (dynamic Bayesian networks). However, these methods differ in the way the distribution of ChIP-seq signals for each chromatin state is modeled. ChromHMM [8, 21, 26], one of the two methods applied by the ENCODE consortium, requires binarized ChIP-seq signals that are then modeled with independent Bernoulli distributions (conditioning on the hidden state). Consequently, the performance of ChromHMM depends on the non-trivial choice of a proper binarization cutoff. Although the default binarization cutoff proposed by ChromHMM performs relatively well in practice, the quantitative information is lost with this approach. This is especially important for distinguishing promoters from enhancers since these elements are both marked with H3K4me1 and H3K4me3, but at different ratios [27]. Segway [9, 22], the other method applied by the ENCODE consortium, uses independent Gaussian distributions of transformed (by the hyperbolic sine function) and smoothed ChIP-seq signal. Although Segway preserves some quantitative information, the transformation of the original count data leads to variance estimation difficulties for very low counts. Therefore, Segway further makes the strong assumption that for a given track, all states have the same variance. Recently, EpicSeg [28] used a negative multinomial distribution to directly model the read counts without the need for data transformations. However, the EpicSeg model leads to a common dispersion (the parameter adjusting the variance of the negative multinomial) for all tracks. Moreover, EpicSeg does not provide other way to correct for sequencing depth than down-sampling, which makes it inappropriate to the analysis of data sets with multiple cell types with varying library sizes. (Down-sampling, i.e. restricting all libraries to the size of the smallest one discards relevant information from all the better covered libraries). Also, EpicSeg has been applied only to three cell types so far [28]. These methods not only differ in their modeling assumptions but also lead to very different results. In the K562 cell line for instance, ChromHMM identified 22,323 enhancers [11], Segway 38,922 enhancers [11], and EpicSeg 53,982 enhancers [28]. Altogether, improved methods and detailed benchmarking analyses are required for a reliable annotation of transcriptional cis-regulatory elements.

Here we propose a new unsupervised genome segmentation algorithm, GenoSTAN (***Geno***mic ***St***ate ***An***notation from sequencing experiments), which overcomes limitations of current state-of-the-art models. GenoSTAN learns chromatin states directly from sequencing data without the need of data transformation, while still having track- and state-specific variance models. We applied GenoSTAN to a total of 127 cell types and tissues covering 16 datasets of ENCODE and all 111 datasets of the Roadmap Epigenomics project as well as four additional (three used in previous studies, one from this study) ENCODE ChIP-seq dataset for the K562 cell line. GenoSTAN performed better in almost every comparison when benchmarked against Segway, ChromHMM and EpicSeg segmentations using independent evidence for activity of promoter and enhancer regions. Co-binding analysis of TFs reveals that promoters and enhancers both shared the Polymerase II core transcription machinery and general TFs, but they are bound by distinct TF regulatory modules and differ in many biophysical properties. Moreover, GenoSTAN enhancer and promoter annotations had a higher enrichment for complex trait-associated genetic variants than previous annotations, demonstrating the advantage of GenoSTAN and our chromatin state map to understand genotype-phenotype relationships and genetic disease.

## Materials and Methods

### Availability of GenoSTAN and chromatin state annotations

GenoSTAN is freely available from http://bioconductor.org/ as part of our previously published R/Bioconductor package STAN [29]. All chromatin state annotations can be downloaded from http://i12g-gagneurweb.in.tum.de/public/paper/GenoSTAN.

### Motivation of Poisson-lognormal and negative binomial emissions

The Poisson-lognormal and the negative binomial distribution can be thought of as extensions of the Poisson distribution that allow for greater variance. We will now motivate both distributions from a Poisson distribution with a prior on the mean of the Poisson.

Suppose that *X* ∼ *Poisson*(*x*|Λ) is a Poisson random variable and Λ ∼ *Gamma*(λ|*α*, *β*). From this we can derive the negative binomial with success rate *p* and size *r*:

$$\begin{array}{ccc} \mathrm{Pr}\left(X=x|\alpha ,\beta \right) & = & \int_{0}^{\infty }Poisson\left(x|\lambda \right)Gamma\left(\lambda |\alpha =r,\beta =\frac{p}{1-p}\right)d\lambda \\ & = & \int_{0}^{\infty }\frac{\lambda^{x}}{x!}e^{-\lambda }\lambda^{r-1}\frac{e^{-\lambda \frac{1-p}{p}}}{{\left(\frac{p}{1-p}\right)}^{r}\Gamma \left(r\right)}d\lambda \\ & = & \frac{\Gamma \left(r+x\right)}{x!\Gamma \left(r\right)}p^{x}{\left(1-p\right)}^{r}\;\text{where }r>0,p\in \left[0,1\right] \end{array}$$

In order to increase interpretability in the context of read counts, we re-parameterize this with mean $\mu =\frac{r\left(1-p\right)}{p}$:

$$\mathrm{Pr}\left(X=x|\mu ,r\right)=\frac{\Gamma \left(r+x\right)}{x!\Gamma \left(r\right)}{\left(\frac{r}{r+\mu }\right)}^{x}{\left(1-\frac{r}{r+\mu }\right)}^{r}\;\text{where }\mu >0$$

The Poisson-lognormal distribution can be motivated likewise. Assume that *X* ∼ *Poisson*(*x*|Λ) is a Poisson random variable and Λ∼N(log(λ)|μ,σ). Then the Poisson-lognormal is given by [30]:

$$\begin{array}{ccc} \mathrm{Pr}\left(X=x|\mu ,\sigma \right) & = & \int_{0}^{\infty }Poisson\left(x|\lambda \right)N\left(\log\left(\lambda \right)|\mu ,\sigma \right)d\lambda \\ & = & \frac{\sqrt{2\pi \sigma^{2}}}{x!}\int_{0}^{\infty }\lambda^{x-1}e^{-\lambda }e^{-\frac{{\left(\log\left(\lambda \right)-\mu \right)}^{2}}{2\sigma^{2}}}d\lambda \end{array}$$

A closed form solution for this distribution does not exist. Thus numerical integration is needed to calculate probabilities, which is done in GenoSTAN by using the R package poilog [31, 32].

### Optimization of Poisson-lognormal and negative binomial emissions

Let $O=(o_{0},...,o_{T}),\;o_{t}={\left(o_{t,d}\right)}_{d\in D}\in {\mathbb{N}}_{0}^{D}$ be an observational sequence of |D|-dimensional count vectors *o*_*t*_. An HMM assumes that each observation *o*_*t*_ is *emitted* by a corresponding hidden (unobserved) variable *s*_*t*_, *t* = 0, …, *T*. A hidden variable can assume values from a finite set of states K. Each state k∈K is associated to an emission distribution *ψ*_*k*_, which defines the probability of making a certain observation, *ψ*_*k*_(*o*_*t*_). GenoSTAN assumes that the components *o*_*t*,*d*_, d∈D, i.e. the individual data tracks (or chromatin modifications), of a single observation *o*_*t*_ at position *t* are independent given the hidden state, and hence $\psi_{k}(o_{t})=\prod_{d\in D}\psi_{k,d}(o_{t,d})$. The value of *s*_*t*_ determines the probability of observing *o*_*t*_ by *Pr*(*o*_*t*_∣*s*_*t*_) = *ψ*_*s*_*t*__(*o*_*t*_). HMM learning is carried out using the Baum-Welch algorithm [25]. The optimization problem for the parameters of a single emission distribution *ψ*_*i*,*d*_ can be written as

$$\underset{\psi_{i,d}}{\arg\max}\sum_{t=0}^{T}\mathrm{Pr}\left(s_{t}=i|O\right)\log\psi_{i,d}\left(o_{t,d}\right)\;,$$

where $Pr(s_{t}=i|O)$ is calculated efficiently by the Forward-Backward algorithm, and *ψ*_*i*,*d*_ is maximized within the class of negative binomial or Poisson-lognormal distributions. An analytical solution for this problem does not exist. Thus, we resort to numerical optimization. As indicated by [28], the above formula can be very costly to compute, since the function needs to evaluate a sum over the complete observation sequence (i.e. the complete binned genome) in each iteration. However, computations are greatly simplified by grouping together observations *o*_*t*,*d*_ with the same count number. Let $C_{d}$ be the set of unique read counts *c* in dimension *d*. Then the following terms can be precomputed for all $c\in C_{d}$ before optimization:

$$f\left(c\right)=\sum_{t;\;o_{t,d}=c}\mathrm{Pr}\left(s_{t}=i|O\right)$$

The objective function becomes

$$\underset{\psi_{i,d}}{\arg\max}\sum_{c\in C_{d}}f\left(c\right)\log\psi_{i,d}\left(c\right)$$

which avoids redundant calculations of *ψ*_*i*,*d*_(*o*_*t*_), *t* = 0, …, *T*, and greatly reduces complexity since $\left|C_{d}\right|\ll T$.

### Correction for library size

The sequencing depth can be very different between experiments. GenoSTAN addresses this problem by using pre-computed scaling factors to correct for varying sequencing depths for a data track between cell types. In this work, the ‘total count’ method is used [33]. Let ℒ be the set of cell types and *r*_*d*,*l*_ the number of reads of data track d∈D in cell line l∈ℒ. The scaling factor is then computed as

$$s_{d,l}=\frac{1}{r_{d,l}}\cdot \frac{\sum_{k\in L}r_{d,k}}{\left|L\right|}$$

The probability of an observation *o*_*t*,*l*_ was $\text{Pr}\left(o_{t,l}|\frac{\mu }{s_{d,l}},r\right)$ in the case of negative binomial and $\text{Pr}\left(o_{t,l}|\text{log}\left(\frac{\mu }{s_{d,l}}\right),\sigma \right)$ in the case of Poisson-lognormal emissions. More robust estimation of library sizes (e.g. [34]) can be used in combination with our software GenoSTAN.

### Model initialization

The initialization of model parameters is crucial for HMMs since the EM algorithm is a gradient method which converges to a local maximum. K-means is a widely used approach to derive an initial clustering to estimate model parameters [25]. In order to make this approach applicable to sequencing data, we added a pseudocount and log-transformed the data before k-means clustering. However, without further processing k-means rarely converged and the procedure was slow on the complete data set. To address these issues, we further processed and filtered the data. First, a threshold for signal enrichment for each data track is calculated using the default binarization approach of ChromHMM [8]. The threshold is the smallest discrete number *n*_*d*_ > 0 such that Pr(*X* > *n*_*d*_) < 10^−4^ where *X* is a Poisson random variable with mean $\lambda_{d}=\frac{\sum_{t=0}^{T}o_{t,d}}{T+1}$. All *o*_*t*,*d*_ < *n*_*d*_ were set to 0, which improved convergence of k-means. To improve the speed, all genomic bins *o*_*t*,*d*_ where $\forall d\in D:\;o_{t,d}=0$ were removed and defined as a ‘background cluster’. K-means was then run on the rest of the data with |K|−1 clusters. This clustering (the ‘background’ and k-means clusters) was then used to derive an initial estimate of emission function parameters. Initial state and transition probabilities were initialized as uniform.

### Data preprocessing

Dataset 1 (K562 ENCODE) sequencing data (for each mark, all available experiments from ENCODE were used) was mapped to the hg20/hg38 (GRCh38) genome assembly (Human Genome Reference Consortium) using Bowtie 2.1.0 [35]. Samtools [36] was used to quality filter SAM files, whereby alignments with MAPQ smaller than 7 (-q 7) were skipped. To obtain midpoint positions of the ChIP-Seq fragments, the (single end) reads were shifted in the appropriate direction by half the average fragment length as estimated by strand coverage cross-correlation using the R/Bioconductor package chipseq [37]. Next, ChIP-Seq tracks were summarized by the number of fragment midpoints in consecutive bins of 200 bp width. The data for the 127 ENCODE and Roadmap Epigenomics cell types was downloaded as preprocessed tagAlign files (hg19) from the Roadmap Epigenomics supplementary website [13]. Preprocessed ENCODE tagAlign files (hg19) for data set 2 and 4 were downloaded from: http://www.broadinstitute.org/~anshul/projects/encode/rawdata/mapped/jan2011/noMultiMapTagAlign/.

For dataset 2, all available ENCODE experiments were used for each mark (as in [23]). For dataset 4, only the experiments from the Bernstein lab as described in [21] were used. For the Roadmap Epigenomics datasets and datasets 2 and 4, fragment length was estimated using the R/Bioconductor package chipseq and reads were shifted by the fragment half size to the average fragment midpoint [37]. The genome was partitioned into 200bp bins and reads were counted within each bin. The count matrix for dataset 3 was kindly provided by the EpicSeg authors.

### Model fitting of GenoSTAN

GenoSTAN was fitted on the complete data of dataset 1. The signal used for GenoSTAN model training on datasets 2, 3 and 4 was extracted from ENCODE pilot regions (1% of the human genome analyzed in the ENCODE pilot phase [38]). For the Roadmap Epigenomics datasets, ENCODE pilot regions were extracted for each cell type. Thus the training data amounted to an equivalent of 20% of the human genome for the models learned on 20 cell types and 127% for the models learned on all 127 cell types. The GenoSTAN-nb-20 model was learned in one day, the GenoSTAN-Poilog-20 model in two days using 10 cores. Model learning on all 127 cell types and tissues using 10 cores took three (GenoSTAN-nb-127) and six days (GenoSTAN-Poilog-127). Precomputed library size factors were used to correct for variation in read coverage.

### Model fitting of ChromHMM, Segway and EpicSeg

For application with ChromHMM, count data was downsampled to a common library size (equal to the size of the smallest data set). The data was binarized as described in [8] and ChromHMM was fitted with default parameters. We made sure the model fitting converged by observing a plateau of the log-likelihood. Before applying Segway, the data was transformed using the hyperbolic sine function [9] and a running mean over a 1kb sliding window was computed to smooth the data. Segway was fitted on ENCODE pilot regions using a 200bp resolution. EpicSeg was fitted on the untransformed count data with default parameters.

### Processing of chromatin state annotations and external data

All state annotations and external data were lifted to the hg20/hg38 (GRCh38) genome assembly using the liftOver function from the R/Bioconductor package rtracklayer [39]. Overlap of state annotations with external data was calculated with GenomicRanges [40]. TT-Seq data was used from [41]. All 86,676 unfiltered transcribed region calls were used for benchmarking. Transcription units on opposing strands were merged (yielding 60,606 non-strand-specific transcribed regions) before overlapping the non-strand-specific chromatin state annotation. All external data used in the analyses can be download from http://i12g-gagneurweb.in.tum.de/public/paper/GenoSTAN/.

### Computation of area under curve

AUC values were calculated on Benchmark set I for GenoSTAN, ChromHMM, Segway and EpicSeg. To this end, a segmentation was transformed into a binary classifier and evaluated as follows. Each 200bp bin in the genome overlapping with HOT (TSSs) regions was considered as ‘true condition’, the rest as ‘false’. For each state *S* the precision for recalling HOT (TSS) regions was calculated as the fraction of all segments annotated with *S* that overlapped with a HOT (TSS) region. States were then sorted by decreasing precision. The rank of each state was used as score in the prediction of HOT (TSS) regions on each 200bp bin in the genome, which was then used to calculate AUC values.

### Analysis of transcription factor (co-)binding

TF enrichment in chromatin states was calculated as described earlier [42]. Let *TF*^*nt*^ be the total number of nucleotides in the binding sites (peaks) a TF and $TF_{s}^{nt}$ the number of nucleotides in the binding sites that overlap with state *s*. Further let *s*^*nt*^ be the total number of nucleotides in the genome covered by state *s* and let *l* be the length of the genome. TF enrichment is then calculated as $\frac{TF_{s}^{nt}/TF^{nt}}{s^{nt}/l}$. For each TF, enrichments were normalized to sum up to 1 across all 18 chromatin states (GenoSTAN-Poilog-K562). The co-binding rate was calculated as the frequency of binding sites of two TFs that co-occur in a chromatin state divided by the number of all binding sites of the two TFs (Jaccard index).

### Tissue-specific enrichment of disease- and complex trait-associated variants in regulatory regions

The GWAS catalog was obtained from the gwascat package from Bioconductor [37, 43]. Statistical testing was carried out in a similar manner as described in [13]. The enrichment of SNPs from individual genome-wide association studies was calculated for traits with at least 20 variants. SNPs for each trait were overlapped with promoter and enhancer regions and tested against the rest of the GWAS catalogue as background using Fisher’s exact test. P-values were adjusted for multiple testing using the Benjamini & Yekutieli correction [44], which applies under any types of dependencies among the null hypotheses. In order to calculate the recall and frequency of SNPs, promoter and enhancer states were randomly sampled until a genomic coverage of 2% for enhancers and 1% of promoters was reached. This was done to control for the fact that methods can differ among each other regarding the length of the promoters and enhancers they predict. This procedure was repeated 100 times enabling the calculation of 95% confidence intervals.

## Results and Discussion

### Modeling of sequencing data with Poisson-lognormal and negative binomial distributions

**Fig 1:**
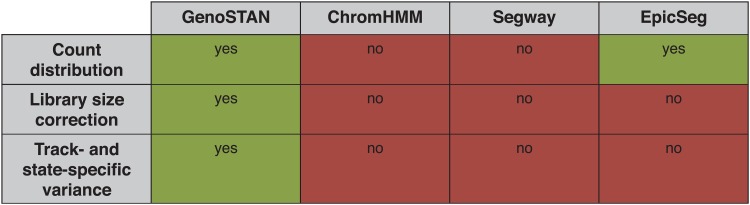
Overview of chromatin state annotation methods. Comparison of features of GenoSTAN against three previous chromatin state annotation algorithms.

We developed a new genomic segmentation algorithm, GenoSTAN, which implements hidden Markov models with more flexible multivariate count distributions than previously proposed. Specifically, GenoSTAN supports two multivariate discrete emission functions, the Poisson-lognormal distribution and the negative binomial distribution. For the sake of reducing running time, the components of these multivariate distributions are assumed to be independent (conditioning on the hidden state). However, the variance is modeled separately for each state and each track, which provides a more realistic variance model than current approaches. To be applicable to data sets with replicate experiments or multiple cell types, GenoSTAN corrects for different library sizes (Methods). All parameters are learnt directly from the data, leaving the number of chromatin states as the only parameter to be manually set. We provide an efficient implementation of the Baum-Welch algorithm for inference of model parameters, which can be run in a parallelized fashion using multiple cores. The method is implemented as part of our previously published R/Bioconductor package STAN [29], which is freely available from http://bioconductor.org/. Altogether, GenoSTAN uniquely combines flexible count distributions, library size correction, and track- and state-specific variance (Fig 1).

**Fig 2:**
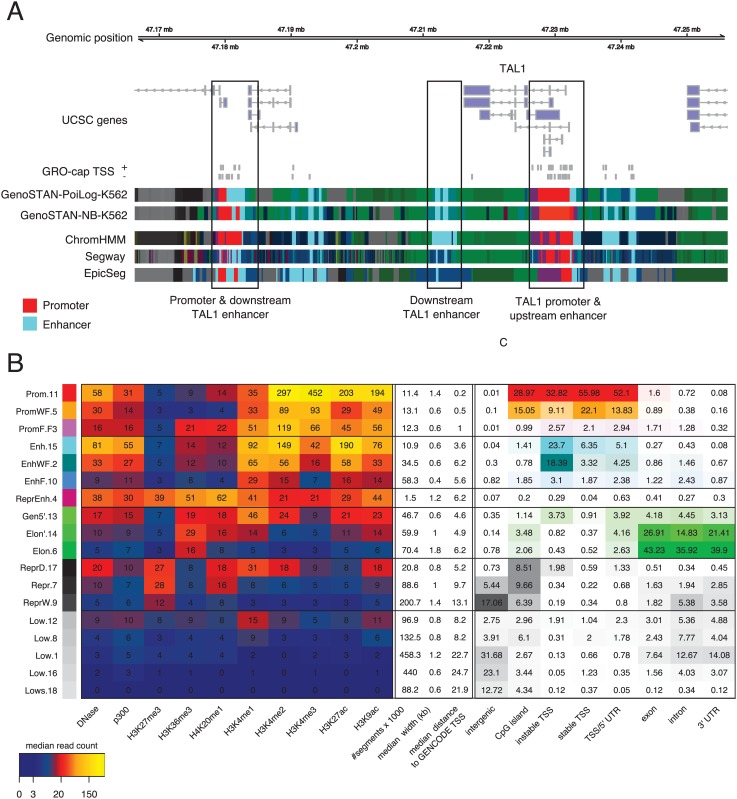
Chromatin states fitted on a dataset using eight histone modifications, P300 and DNase-Seq (dataset 1) using GenoSTAN. (A) GenoSTAN segmentations are shown with published segmentations using ChromHMM-ENCODE [11], Segway-ENCODE [11] and EpicSeg [28] at the TAL1 gene and three known enhancers. GenoSTAN-Poilog-K562 correctly recalls all known promoter and enhancer regions, whereas other methods frequently switch between promoter, enhancer, and other states. (B) Median read coverage of GenoSTAN-Poilog-K562 chromatin states (left), their number of annotated segments in the genome, their median width and distance to the closest GENCODE TSS (middle). The right panel shows recall of genomic regions by chromatin states.

We first fitted two GenoSTAN models, one with Poisson-lognormal emissions (henceforth referred to as GenoSTAN-Poilog-K562 model) and one with negative binomial emissions (GenoSTAN-nb-K562 model) to a dataset of ChIP-seq data of 9 histone modifications, of the histone acetyltransferase P300, and DNA accessibility (by DNase-Seq) data for the K562 cell line at 200 bp binning resolution (Methods). K562 is a major model system to study human transcription and the ENCODE cell line with the largest number of experiments [11]. As pointed out by others [8, 9], there is no purely statistical criterion for choosing the number of states from the data of practical usage in such a setting. In practice, the number of states is manually defined by trading off goodness of fit against interpretability of the model [8, 9, 29]. For GenoSTAN-Poilog-K562, we used 18 chromatin states. For GenoSTAN-nb-K562, we used 23 states, since lower state numbers did not provide enough resolution to give a fine-grained map of chromatin states on this data set. This led to the definition of promoter, enhancer, repressed, actively transcribed and low coverage states, in line with previous studies [21, 22, 28] (Fig 2A and 2B, and S1 Appendix for a description of the identified states). The median read coverage in state segments and genomic distributions were very similar for both the GenoSTAN-Poilog-K562 and the GenoSTAN-nb-K562 models (Fig 2B, S1 Fig). We noticed that GenoSTAN segmentation often gave more accurate annotations of enhancers and promoters compared to previous segmentations for K562 using ChromHMM, Segway and EpicSeg [11, 22, 28, 45], as for instance at the locus of the gene TAL1 (Fig 2A). We then investigated how systematic this improvement was.

### Algorithmic benchmark

**Fig 3:**
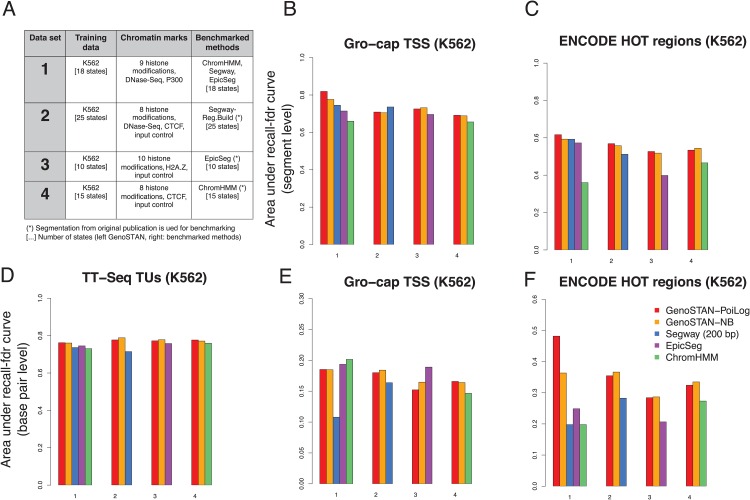
GenoSTAN with other published chromatin state annotation methods applied to four different datasets in K562. (A) Description of the four data sets used for benchmarking. All methods were applied to dataset 1 with 18 states in this study. Datasets 2, 3 and 4 were used in previous studies [21, 23, 28]. Segmentations which were created by the authors of the respective studies were compared to GenoSTAN segmentations using the same number of states. (B-F) Performance of chromatin annotations on each of the dataset 1, 2, 3, and 4 is summarized by the area under the recall-FDR curve for various genomic features. Cumulative FDR and recall are calculated using overlap on state segments level (B,C) or on base pair level (D-F) by subsequently adding states (in order of increasing FDR). S2, S3, S4 and S5 Figs show individual recall-FDR curves for all datasets and segmentations.

The segmentations considered above not only differed for the algorithms but also for the data they have been fitted on. To compare the performance of the algorithms only, we devised benchmarks on common data. First, we benchmarked GenoSTAN and the three alternative methods for a common set of ChIP-seq data of the K562 cell line (Fig 3A, dataset 1 and S1 Appendix). To discard the possibility that this benchmark is favorable to GenoSTAN because we had not optimally applied the other software, we also compared the segmentations obtained by GenoSTAN with the exact same data and number of states than the original segmentations obtained by the authors of the respective methods (datasets 2, 3, and 4).

Transcription initiation activity is not only the hallmark of promoters, but also of enhancers [15, 16, 46, 47]. To benchmark the predictions using independent evidence for transcription initiation, we used published data from a protocol called GRO-cap [46], a nuclear run-on protocol, which very sensitively maps transcription start sites genome-wide. To this end, we sorted for each method chromatin states by their overlap with GRO-cap TSSs by decreasing precision. Starting with the most precise state (i.e. highest overlap with TSSs) we calculated cumulative recall and false discovery rate (FDR) by subsequently adding states with decreasing precision. GenoSTAN-Poilog-K562 had a lower FDR at a similar or higher recall than all other methods (S2A Fig). Only Segway showed a stronger recall when allowing for a lose FDR larger than 20%. However, this was reached with a single state which does not distinguish between promoters and enhancers. As second independent measure, we considered High Occupancy of Target (HOT) regions. Hot regions are genomic regions which are bound by a large number of different transcription-related factors [12], which were shown to function as enhancers [48] and are enriched in disease- and trait-associated genetic variants [49]. HOT regions are not necessarily transcriptionaly active regions and thus provide a different kind of benchmark. As for the benchmark with GRO-cap, the best performing segmentations for HOT regions was GenoSTAN-Poilog-K562 (S2B Fig). Only Segway showed a stronger recall at an FDR over 20%, but again with a single state which does not discriminate promoters from enhancers. We also benchmarked the models for recovering complete transcribed regions at the base pair level, using an independent dataset of transcriptional units obtained by transient transcriptome sequencing (TT-seq), a protocol we recently developed that sensitively identifies transcribed regions [41]. GenoSTAN-Poilog-K562 showed an improved recall of transcribed base pairs up to 20% FDR against all other methods (S3C Fig).

The overall results over data sets 1, 2, 3 and 4 are summarized by area under the recall-FDR curves (Fig 3B–3F) and individual curves provided in S2, S3, S4 and S5 Figs. Overall, GenoSTAN ranked first in 17 out of 20 comparisons, whereby GenoSTAN-Poilog and GenoSTAN-nb showed comparatively good performances. These results include comparisons for recalling GRO-cap and HOT regions at the base-pair level, to control for the possibility that some methods get good performance on segment-level benchmarks by reporting aberrantly long segments. Altogether, this extensive benchmark in the K562 cell line demonstrates that GenoSTAN-Poilog and to a slightly lesser extent GenoSTAN-nb, outperforms current chromatin state annotation algorithms for a variety of genomic features.

Because the K562 cell line is a widely used model system for the study of human transcription, we provide in S1 Appendix a detailed comparison of the advantage of this segmentation over the former K562 chromatin state annotations. This comparisons includes benchmarks for GRO-cap TSS (S6A Fig), HOT regions (S6B Fig), transcription factor binding (S6C Fig), discrimination between enhancers and promoters (S6D and S7 Figs, S1 Table), activity of enhancers from reporter assays (S6E and S6F Fig), and robustness regarding state number (S8 Fig).

### Chromatin state annotation for ENCODE and Roadmap Epigenomics cell types and tissues

We next applied GenoSTAN to 127 cell types and tissues from ENCODE and Roadmap Epigenomics, the largest compendium of chromatin-related data. To this end, we used genomic input and the five chromatin marks H3K4me1, H3K4me3, H3K36me3, H3K27me3, and H3K9me3 that have been profiled across the whole compendium [13] (GenoSTAN-127, S9 and S10 Figs). Moreover, we performed a dedicated analysis to 20 of these cell types and tissues which had three further important data tracks: H3K27ac, H3K9ac and DNase-Seq (GenoSTAN-20, S11 Fig). These further three tracks are important features of active promoters and enhancers, which can lead to more precisely mapped enhancer boundaries [11]. For completeness, each analysis was performed with Poisson-lognormal emission distributions and with negative binomial distributions. We focus primarily on the GenoSTAN-Poilog-127 results because these cover the full compendium. We provide results for the other analyses when relevant.

**Fig 4:**
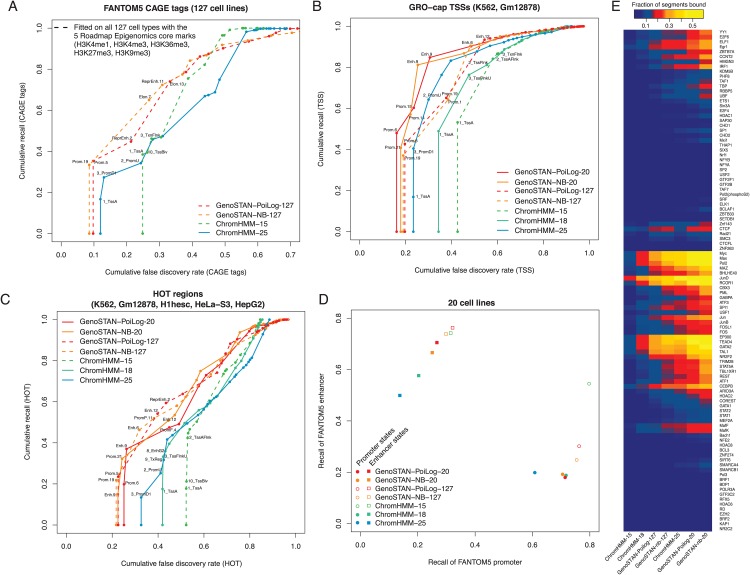
Comparison of GenoSTAN to other published ChromHMM segmentations from the Roadmap Epigenomics project. GenoSTAN was learned on all 127 cell types and tissues (GenoSTAN-127) using the five core marks H3K4me1, H3K4me3, H3K36me3, H3K27me3, H3K9me3 and an input control (ChromHMM-15 was learned on the same data). To improve accuracy additional histone modifications H3K27ac, H3K9ac and DNase-Seq were used to learn another model (GenoSTAN-20) on a subset of 20 cell types and tissues, where the marks were available. (A) Performance of chromatin states in recovering FANTOM5 CAGE tags in 127 cell types. CAGE tags were verlapped with chromatin states wihout the use of cell type information. Cumulative FDR and recall are calculated by subsequently adding states (in order of increasing FDR). (B) Performance of chromatin states in recovering GRO-cap transcription start sites in two cell types where GRO-cap data was available. (C) The same as in (B) for ENCODE HOT regions for five cell types where annotation of HOT regions was available. (D) Recall of FANTOM5 promoters and enhancers by predicted promoters and enhancersis plotted to assess how well models distinguish promoters from enhancers. (E) The fraction of predicted enhancer segments bound by individual TFs is shown for different studies. GenoSTAN enhancers are more frequently bound by TFs than those from other studies.

We performed similar comparisons as for K562 to the three available segmentations from the Roadmap Epigenomics project with 15, 18 and 25 states (ChromHMM-15, -18, and -25). ChromHMM-15 is the segmentation that had been applied to all 127 cell types and tissues [13, 50] and should be compared to GenoSTAN-Poilog-127 which was run on the same data. ChromHMM-18, and -25 had been applied to specific subsets [13, 50]. All methods were less performant than for the K562 annotations, possibly due to lower read coverage or to less rich data. Nonetheless, the GenoSTAN annotations consistently outperformed the existing ones. Specifically, this held when assessing the recovery of FANTOM5 CAGE tags (Fig 4A, assessed for all 127 cell types and tissues), of GRO-cap TSSs (Fig 4B assessed for the cell types with available GRO-cap TSSs), of HOT regions (Fig 4C, assessed for the cell types with available HOT regions), and of transcribed regions (GENCODE genes, S12A Fig, and TT-seq transcribed regions, S12B Fig). Moreover, the GenoSTAN-Poilog-127 model distinguished better promoters from enhancers than ChromHMM-15 when using FANTOM5 annotations for benchmark (Fig 4D, S2 Table). One possible reason for this better discrimination is that the ChromHMM-15 annotation had two states called “Flanking active TSS (TssAFlnk)” and “Transcription at gene 5’ and 3’ (TxFlnk)” that map to either enhancers or promoter states of the GenoSTAN-Poilog-127 segmentation (S13A Fig). Inspection of the fits indicated that the lower accuracy of the ChromHMM promoters and enhancers might be caused by frequent state switching between the promoter and promoter flanking state (S9 Fig for an example at the TAL1 locus). Consistent with this hypothesis, the distance between consecutive promoters was much shorter for the ChromHMM-15 annotation than for the GenoSTAN-Poilog-127 and for the GenoSTAN-nb-127 segmentations (S14A Fig). Also, the ChromHMM-15 segmentations showed weaker agreement across cell types: the number of 200bp-bins annotated with promoter state in only one of the 127 cell types was much larger for ChromHMM-15 (298,859) than for GenoSTAN (133,062 for GenoSTAN-Poilog-127 and 107,417 for GenoSTAN-nb-127, S14B Fig).

The higher accuracy of enhancers for GenoSTAN was also reflected by the enrichment for transcription factor binding in K562 (Fig 4E). For instance 46% (25%) of enhancers were bound by Pol II in the GenoSTAN-Poilog-20 (-127) model, compared to 8%, 18% and 36% in the ChromHMM 15, 18 and 25 state models. Also, the lineage-specific enhancer-binding transcription factor TAL1 binds at 37% (GenoSTAN-Poilog-20) and 27% (GenoSTAN-Poilog-127) of predicted enhancers. Conversely, 13%, 16% and 27% of putative enhancers were bound by TAL1 in the respective 15, 18 and 25 state ChromHMM models (Fig 4E).

Collectively, these results show that the improved performance of GenoSTAN is not specific to the K562 dataset.

### Cell-type specific enrichment of disease- and other complex trait-associated genetic variants at promoters and enhancers

**Fig 5:**
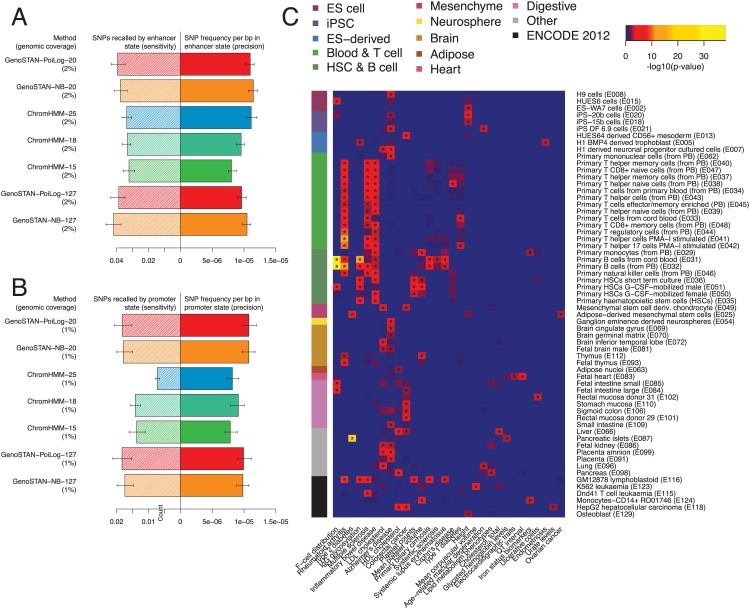
Enrichments of genetic variants associated with diverse traits in enhancers and promoters are specific to the relevant cell types or tissues. (A) Median SNP recall and frequency was calculated for enhancer states in different segmentations by restricting it to a total genomic coverage of 2% (100 samples of random subsetting) to control for different number of enhancer calls between the segmentations. Error bars show the 95% confidence interval. (B) The same as in (A) but for promoters. (C) The heatmap shows the -log10(p-value) of significantly enriched traits in enhancer states (GenoSTAN-Poilog-127, p-value < 0.01, marked by ‘*’). Only cell types and tissues where at least one trait was significantly enriched are shown. P-values were adjusted for multiple testing using the Benjamini-Yekutieli correction.

Previous studies showed that disease-associated genetic variants are enriched in potential regulatory regions [13, 21, 51–54] demonstrating the need for accurate maps of these elements to understand genotype-phenotype relationships and genetic disease. To study the potential impact of variants in regulatory regions on various traits and diseases, we overlapped our enhancer and promoter annotations from 127 cell types and tissues with phenotype-associated genetic markers from the NHGRI genome-wide association studies catalog (NHGRI GWAS Catalog [43]). We note that the functional variants might not be the markers themselves but some other variants that are in linkage with these markers. Therefore this analysis conservatively underestimates the true sensitivity, but, importantly, the same way for all methods. First, we intersected trait-associated variants with enhancer and promoter states (GenoSTAN-Poilog-127). Overall, 37% of all trait-associated SNPs were located in potential enhancers and 7% in potential promoters. The number of traits significantly enriched (at FDR <0.05) with enhancers or promoters in at least one cell type or tissue was similar for GenoSTAN-Poilog-127 (39 traits for GenoSTAN-Poilog-127 for enhancers and 9 traits for promoters) than for the best performing ChromHMM-model (ChromHMM-15, 35 traits for enhancers and 11 traits for promoters, S15A and S15B Fig). We next assessed the sensitivity and the precision for recalling disease- and complex trait-associated markers. To control for the fact that methods can differ among each other regarding the length of the promoters and enhancers they predict, we furthermore computed the recalls of GWAS variants for a fixed genomic coverage. Restricting to a total genomic coverage of 2% (random subsetting, also allowing confidence interval computation, Methods), enhancers of all GenoSTAN models overlapped a higher fraction of GWAS variants at a similar to better per base pair density compared to the current ChromHMM annotations (Fig 5A). The same trend was observed for promoters when restricting to 1% of genomic coverage (Fig 5B). These results also held for weak enhancers (S16 Fig), and when controlling for possible segment length effect by benchmarking at the base-pair level rather than at the segment level to (S17 Fig). The improved overlap with trait-associated variants indicates that GenoSTAN annotation has a higher enrichment for functional elements than the current annotation.

In accordance with previous studies [13, 21] we found that individual variants were strongly enriched in enhancer or promoter states specifically active in the relevant cell types or tissues (Fig 5C, S15C Fig). Variants associated with height were significantly associated with osteoblasts enhancers (at FDR <0.001 here and after). Variants associated with immune response or autoimmune disorders were enriched in B- and T-cell enhancers (Fig 5C) and promoters (S15C Fig). These include for instance autoimmune disease associated SNPs for systemic lupus erythematosus, inflammatory bowel disease, ulcerative colitis, rheumatoid arthritis, and primary biliary cirrhosis. Variants associated with electrocardiographic traits and QT interval were enriched in fetal heart enhancers. SNPs associated with colorectal cancer were enriched in enhancers specific to the digestive system. These results illustrate that the annotation of potential promoters and enhancers generated in this study can be of great use for interpreting genetic variants associated, and underscore the importance of cell-type or tissue-specific annotations.

### A novel annotation of enhancers and promoters in human cell types and tissues

We then compiled the results from the best performing annotations for each cell type and tissue into a single annotation file. The combined annotation files are available from http://i12g-gagneurweb.in.tum.de/public/paper/GenoSTAN. For the combined annotation file, we chose GenoSTAN with Poisson-lognormal in every instance, as it performed best in almost every comparison we conducted. We used the results from dataset 1 for K562, from GenoSTAN-Poilog-20 for the 20 cell types and tissues, and from GenoSTAN-Poilog-127 for all the remaining Roadmap Epigenomics cell types and tissues. Overall, our annotation reports typically between 8,945 and 16,750 (10% and 90% quantiles of number of promoters across all 127 cell types and tissues) active promoters per cell type or tissue. This number is consistent with the typical number of expressed genes per tissue (in 11,953 to 16,869 range, [55]). However, the median width of these elements depends on the data on which the annotation was based. For the GenoSTAN-Poilog-20 segmentation, promoters are much narrower (800bp median) than for the K562 annotations (1.4 kb), suggesting that promoter regions in the 20 cell types more accurately match DNase hypersensitivity sites (DHS) of the core promoter. The number of enhancers per cell type or tissue varied more greatly (between 8,208 and 33,596 for the 10% and 90% quantiles). The large variation of the number of enhancers might be partly due to differences of sensitivity in complex biological samples. Consistent with this hypothesis, much fewer enhancers were identified in tissues than in primary cells and cell lines (S18 Fig) likely because enhancers that are active only in a small subsets of all cell types present of a tissue may be not detected. As more cell-type specific data will be available, improved maps can be generated. The GenoSTAN software, which is publicly available, will be instrumental to update these genomic annotations.

### Promoters and enhancers have a distinct TF regulatory landscape

**Fig 6:**
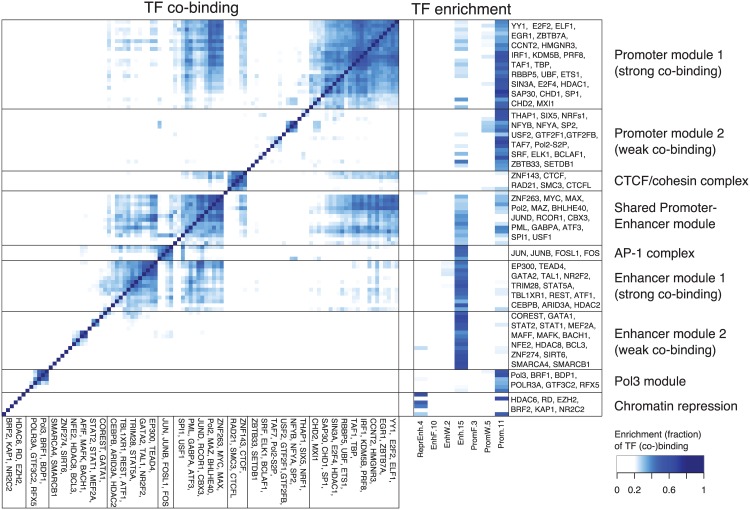
Promoters and enhancers have a distinctive TF regulatory landscape. Co-binding (left) and enrichment of transcription factor binding sites (right) in chromatin states (GenoSTAN-Poilog-K562) for 101 transcription factors in K562 reveals TF regulatory modules with distinct binding preferences for promoters, enhancers and repressed regions. The co-binding is depicted as the frequency of binding sites of two TFs that co-occur in a chromatin state divided by the number of all binding sites of the two TFs (Jaccard index). For each TF, enrichments were normalized to sum up to 1 across all 18 chromatin states of GenoSTAN-Poilog-dataset 1.

The biochemical distinction between enhancers and promoters is a topic of debate [6, 7]. We explored to which extent enhancers and promoters are differentially bound by TFs using the K562 cell line dataset because i) we obtained the most accurate annotation for this cell line (GenoSTAN-Poilog-K562, dataset 1) and ii) ChIP-seq data was available for as many as 101 TFs in this cell line [11]. Nine TF modules were defined by clustering based on binding pattern similarity across enhancers and promoters (Methods, Fig 6). These 9 TF modules were further characterized by the propensity of their TFs to bind promoters, enhancers or both (Fig 6). In accordance with previous studies [42, 56], this recovered many complexes and promoter-associated and enhancer-associated proteins, including the CTCF/cohesin complex (CTCF, Rad21, SMC3, Znf143), the AP-1 complex (Jun, JunB, FOSL1, FOS), Pol3, promoter and enhancer associated modules, and factors associated with chromatin repression (EZH2, HDAC6).

Moreover, the modules identified provided insights into the distinction of promoters and enhancers. On the one hand, some TFs are common to both enhancers and promoters, which supports previous reports [7, 15]. In accordance with the recent finding of widespread transcription at enhancers [46], Pol II and multifunctional TFs Myc, Max, and MAZ [57] are part of a TF module—which we called the Promoter-Enhancer-Module (PEM)—which had approximately equal binding preferences for promoter and enhancer states, but also co-localized with other TFs specifically binding enhancers or promoters (Fig 6).

On the other hand enhancers and promoters were also bound by distinct TFs, which is consistent with previously reported TF co-occurrence patterns at gene-proximal and gene-distal sites [42, 56]. Among the promoter and enhancer-associated proteins we defined Promoter module 1 and 2 (PM1, PM2), Enhancer module 1 and 2 (EM1, EM2), which had a strong preference for binding either a promoter or an enhancer, but exhibited different co-binding rates (Fig 6). Promoter module 1 contained TFs which were specifically enriched in promoter states and associated with basic promoter functions, such as chromatin remodeling (CHD1, CHD2), transcription initiation or elongation (TBP, TAF1, CCNT2, SP1) and other TFs involved in the regulation of specific gene classes (e.g. cell cycle: E2F4) [57]. However, it also included TFs known as transcriptional repressors (e.g. Mxi1, a potential tumor suppressor, which negatively regulates Myc). While TFs in PM1 showed a high co-binding rate, PM2 factors exhibited low co-binding. This might be partially explained by lower efficiency of the ChIP, since PM2 also contained general TFs such as TFIIB, TFIIF or the Serine 2 phospho-isoform of Pol II, which are expected to co-localize with other general TFs from PM1.

EM1 contained TFs with high co-binding rate, which included TAL1, an important lineage-specific regulator for erythroid development (K562 are erythroleukemia cells) and which had been shown to interact with CEBPB, GATA1 and GATA2 at gene-distal loci [56, 58]. It also contained the enhancer-specific transcription factor P300 [59] and transcriptional activators (e.g. ATF1) and repressors (e.g. HDAC2, REST) [57]. Analogously to PM2, EM2 contained enhancer-specific transcriptional activators and repressors with a low co-binding rate.

Altogether this analysis highlights the common and distinctive TF binding properties of enhancers and promoters.

## Conclusion

We introduced GenoSTAN, a method for *de novo* and unbiased inference of chromatin states from genome-wide profiling data. In contrast to previously described methods for chromatin state annotation, GenoSTAN directly models read counts, thus avoiding data transformation and the manual tuning of thresholds (as in ChromHMM and Segway), and variance is not shared between data tracks or states (as in EpicSeg and Segway) [8, 9, 28]. GenoSTAN is released as part of the open-source R/Bioconductor package STAN [29, 32, 37], which provides a fast, parallel implementation that can process data from 127 human cell types in less 3–6 days (GenoSTAN-Poilog-127: 6 days, -nb: 3 days).

Application of GenoSTAN significantly improved chromatin state maps of 127 cell types and tissues from the ENCODE and Roadmap Epigenomics projects [11, 13]. Binding of enhancer-associated co-activator CBP and histone acetyltransferase P300 was used by several studies for the genome-wide prediction of enhancers [27, 59, 60]. From these predictions a distinctive chromatin signature for promoters and enhancers was derived based on H3K4me1 and H3K4me3 [27]. In particular, the ratio H3K4me1/H3K4me3 was found to be low at promoters, in comparison to enhancers. Active and poised enhancers could also be distinguished by presence or absence of H3K27me3 and H3K9me3 [61]. All these features could be confirmed by GenoSTAN, making it a promising tool for the biochemical characterization of enhancers and promoters. Moreover, extensive benchmarks based on independent data including transcriptional activity, TF binding, cis-regulatory activity, and enrichment for complex trait-associated variants showed the highest accuracy of GenoSTAN annotations over former genome segmentation methods.

We have implemented two count distributions, the Poisson log-normal and the negative binomial distribution. Both distributions capture over-dispersion. The negative binomial distribution is a popular distribution for modeling count data, because it is part of the exponential family, and it is used for many genomics applications. However, in almost every benchmark we performed, the Poisson log-normal distribution turned out to give better results. We therefore suggest to use GenoSTAN with the Poisson-lognormal distribution. Independently of which of the Poisson-lognormal or the negative binomial, GenoSTAN generally improved over former methods. This indicates that the importance of i) a count distribution and ii) modeling a state- and track-specific variance. GenoSTAN tended to attribute more states to regions with low coverage compared to ChromHMM, which is based on a binarization of the data (S12 Fig). The biological interpretation of these multiple states with low coverage is unclear. This is not a problem in practice because all these states are typically considered as a single group. Different noise model could be investigated to cope with these low count regions more uniformly, for instance with mixture models such as the zero-inflated poisson. In contrast, ChromHMM provided more states regions with high coverage (S12 Fig). Although potentially more relevant, the qualitative distinction of these states also has unclear biological interpretation. In practice, these are often grouped with other states for further analysis [13]. If wished, capturing more states with high coverage could be obtained with GenoSTAN by increasing the number of states.

The GenoSTAN annotation sheds light on the common and distinctive features of promoters and enhancers, which currently are an intense subject of debate [6, 7]. Among other characteristics, a shared architecture of promoters and enhancers was proposed based on the recent discovery of widespread bidirectional transcription at enhancers [7, 46, 47]. This was supported by the observation that enhancers, which are depleted in CpG islands have similar transcription factor (TF) motif enrichments as CpG poor promoters [15]. However, another study showed that TF co-occurrence differed between gene-proximal and gene-distal sites [42, 56]. GenoSTAN chromatin states revealed a very distinct TF regulatory landscape of these elements and therefore suggest that promoters and enhancers are fundamentally different regulatory elements, both sharing the binding of the core transcriptional machinery. Our annotation of enhancers and promoters will be a valuable resource to help characterizing the genomic context of the binding of further TFs.

Indirectly, our analysis showed that chromatin state annotations are better predictors of enhancers than the transcription-based definition provided by the FANTOM5 consortium [15]. While FANTOM5 enhancers are an accurate predictor for transcriptionally active enhancers, the sensitivity remains poor (only 4,263 enhancers were called by overlap with GRO-cap TSSs and DHS, which is less than the estimated number of transcribed genes, for K562 cells compared to about 20,000–30,000 for ChromHMM and 10,000–20,000 for GenoSTAN). Although, the sensitivity of the transcription-based approach can increase with transient transcriptome profiling [62–63] or nascent transcriptome profiling [64], the chromatin state data undoubtedly add valuable information for the identification of promoters and enhancers. Because it models count data, GenoSTAN analysis can in principle also integrate RNA-seq profiles, for instance using it in a strand-specific fashion [29].

Systematic identification of cis-regulatory active elements by direct activity assays is notoriously difficult. STARR-Seq for instance is a high-throughput reporter assay for the *de novo* identification of enhancers [5]. It was previously used to identify thousands of cell-type specific enhancers in *Drosophila*, but has not been applied genome-wide to human yet. Moreover, STARR-Seq makes rigid assumptions about the location of the enhancer element with respect to the promoter, and it does not account for the native chromatin structure. This might identify regions that are inactive *in situ* [5]. Other experimental assays for the validation of predicted ENCODE enhancers lead to different results [3, 4]. Complementary to these approaches, the systematic evaluation of cis-regulatory activity based on candidate regions in human cells have made progress with the advent of high-throughput CRISPR perturbation assays [65]. Because it requires candidate cis-regulatory regions in a first place, such approach will benefit from improved annotation maps as the one we are providing.

Thus, we foresee GenoSTAN to be instrumental in future efforts to generate robust, genome-wide maps of functional genomic regions like promoters and enhancers.

## Supporting Information

S1 Fig Summary statistics for GenoSTAN-nb-K562 model. Median read coverage of GenoSTAN-nb-K562 chromatin states (left), their number of annotated segments in the genome, their median width and distance to the closest GENCODE TSS (middle). The right panel shows recall of genomic regions by chromatin states.
(PDF)

S2 Fig Algorithmic benchmark of GenoSTAN with ChromHMM, EpicSeg and Segway on dataset 1. (A) Performance of chromatin states in recovering GRO-cap transcription start sites using state segments. Cumulative FDR and recall are calculated using overlap with state segments by subsequently adding states (in order of increasing FDR). (B) The same as in (A) for ENCODE HOT regions. (C) TT-Seq transcribed regions were overlapped with state annotations on bp level and cumulative FDR and recall were calculated. (D,F) Performance of chromatin states in recovering GRO-cap transcription start sites and ENCODE HOT regions using bp overlap.
(PDF)

S3 Fig Algorithmic benchmark of GenoSTAN with Segway on dataset 2. (A) Performance of chromatin states in recovering GRO-cap transcription start sites using state segments. Cumulative FDR and recall are calculated using overlap with state segments by subsequently adding states (in order of increasing FDR). (B) The same as in (A) for ENCODE HOT regions. (C) TT-Seq transcribed regions were overlapped with state annotations on bp level and cumulative FDR and recall were calculated. (D,F) Performance of chromatin states in recovering GRO-cap transcription start sites and ENCODE HOT regions using bp overlap.
(PDF)

S4 Fig Algorithmic benchmark of GenoSTAN with EpicSeg on dataset 3. (A) Performance of chromatin states in recovering GRO-cap transcription start sites using state segments. Cumulative FDR and recall are calculated using overlap with state segments by subsequently adding states (in order of increasing FDR). (B) The same as in (A) for ENCODE HOT regions. (C) TT-Seq transcribed regions were overlapped with state annotations on bp level and cumulative FDR and recall were calculated. (D,F) Performance of chromatin states in recovering GRO-cap transcription start sites and ENCODE HOT regions using bp overlap.
(PDF)

S5 Fig Algorithmic benchmark of GenoSTAN with ChromHMM on dataset 4. (A) Performance of chromatin states in recovering GRO-cap transcription start sites using state segments. Cumulative FDR and recall are calculated using overlap with state segments by subsequently adding states (in order of increasing FDR). (B) The same as in (A) for ENCODE HOT regions. (C) TT-Seq transcribed regions were overlapped with state annotations on bp level and cumulative FDR and recall were calculated. (D,F) Performance of chromatin states in recovering GRO-cap transcription start sites and ENCODE HOT regions using bp overlap.
(PDF)

S6 Fig Comparison of GenoSTAN-(NB/PoiLog)-K562 (dataset1) to other published segmentations (‘ChromHMM-ENCODE’ [11, 22], ‘ChromHMM-dataset4’ [21], ‘ChromHMM-15’, ‘-18’ and ‘-25’ [13], ‘Segway-ENCODE’ [11, 22], ‘Segway-nmeth’ [9], ‘Segway-dataset2’ [23] and EpicSeg-dataset3 [28]. (A) Performance of chromatin states in recovering GRO-cap transcription start sites. Cumulative FDR and recall are calculated by subsequently adding states (in order of increasing FDR). (B) The same as in (A) for ENCODE HOT regions. (C) The fraction of predicted enhancer segments bound by individual TFs is shown for different studies. GenoSTAN enhancers are more frequently bound by TFs than those from other studies. (D) Recall of FANTOM5 promoters and enhancers which are active in K562 (i.e. overlapping with a GRO-cap TSS and an ENCODE DNase hypersensitivity site) by predicted promoters and enhancers is plotted to assess how well models distinguish promoters from enhancers. (E) Predicted enhancers show significantly higher activity than repressed and low coverage regions as measured by a reporter assay (‘*’, ‘**’ and ‘***’ indicate p-values <0.05, 0,01 and 0.001). (F) Comparison of experimental measures of enhancer activity between different studies.
(PDF)

S7 Fig Overlap of promoter and enhancer annotations in K562 between different studies. (A) Heatmap of pairwise overlap (Jaccard index) of promoter (red) and enhancer (orange) state annotations from different studies. Rows and columns were ordered by separate clustering of promoter and enhancer overlaps. (B) Distribution of pairwise Jaccard indices for strong promoters and enhancers (off-diagonal elements of promoter and enhancer sub-matrices from (A)).
(PDF)

S8 Fig Algorithmic benchmark of GenoSTAN, ChromHMM, Segway and EpicSeg on dataset 1. Comparison of chromatin segmentation algorithms with respect to their ability to call GRO-cap transcription start sites (left panels) and ENCODE HOT regions (right panels), as a function of the state number used in the respective algorithm (x-axes). All models were learned on dataset 1. (A-B) For each model, the state with highest precision in recalling HOT (respectively TSS) regions is shown. (C-D) For each model, an area under curve (AUC) score (see Methods) is plotted to asses the spatial accuracy of a genome segmentation.
(PDF)

S9 Fig GenoSTAN and ChromHMM segmentations on Roadmap Epigenomics data around the TAL1 gene in K562. GenoSTAN models fitted on a subset of 20 and all 127 cell types and tissues from Roadmap Epigenomics are compared to ChromHMM models with 15, 18 and 25 states at the TAL1 gene in K562.
(PDF)

S10 Fig GenoSTAN models with 20 states fitted on five core marks of all 127 cell types and tissues from Roadmap Epigenomics. (A) Median read coverage of GenoSTAN-Poilog-127 chromatin states (left), their number of annotated segments in the genome, their median width and distance to the closest GENCODE TSSs of segments (middle). The right panel shows recall of genomic regions by chromatin states. (B) The same as (A) for GenoSTAN-nb-127.
(PDF)

S11 Fig GenoSTAN models with 25 states fitted on nine chromatin marks on a subset of 20 cell types and tissues form Roadmap Epigenomics. (A) Median read coverage of GenoSTAN-Poilog-20 chromatin states (left), their number of annotated segments in the genome, their median width and distance to the closest GENCODE TSSs of segments (middle). The right panel shows recall of genomic regions by chromatin states. (B) The same as (A) for GenoSTAN-nb-20.
(PDF)

S12 Fig Algorithmic benchmark of GenoSTAN-(Poilog/NB)-127 and ChromHMM-15. GenoSTAN-(Poilog/NB)-127 is shown in red/orange, ChromHMM-15 in green. All three models were learned on the same data (H3K4me1, H3K4me3, H3K36me3, H3K27me3, H3K9me3 and an input control). GenoSTAN models were learned with 20, ChromHMM-15 with 15 states. In both plots, cumulative FDR and recall are calculated by subsequently adding states (in order of increasing FDR). Performance of chromatin states in recovering GENCODE gene annotations (A) and TT-seq transcribed regions (B) in K562 at bp level.
(PDF)

S13 Fig Overlap (base pair) between GenoSTAN-Poilog-127 and ChromHMM-15 is shown. (A) Rows were normalized to sum up to 1. (B) Columns were normalized to sum up to 1.
(PDF)

S14 Fig Comparison of stability of promoter and enhancer annotation between GenoSTAN-Poilog-127, GenoSTAN-NB-127 and ChromHMM-15. (A) Estimated cumulative distribution of promoter states within a certain distance along genome in K562. The number of 200bp bins that are annotated in only one (out of 127) cell types are counted for promoters (B) and enhancers (C) for the different segmentations.
(PDF)

S15 Fig Enrichments of genetic variants associated with diverse traits in enhancers and promoters are specific to the relevant cell types. (A) The number of traits which are enriched in enhancer states in at least one cell type or tissue is plotted for p-values < 0.05. (B) The same as in (A) but for promoters. (C) The heatmap shows the -log10(p-value) of significantly enriched traits in promoter states (GenoSTAN-Poilog-127, p-value < 0.05, marked by ‘*’). P-values were adjusted for multiple testing using the Benjamini-Yekutieli correction.
(PDF)

S16 Fig Frequency of SNPs in enhancers precicted from Roadmap Epigenomics data. Median SNP recall and frequency was calculated for weak intergenic enhancer or enhancer flanking states (ChromHMM-18: ‘10_EnhA2’, ChromHMM-25: ‘14_EnhA2’, GenoSTAN-Poilog-20: ‘EnhF.13’, GenoSTAN-NB-20: ‘EnhF.12’, GenoSTAN-Poilog-127: ‘EnhW.9’, GenoSTAN-NB-127: ‘EnhW.8’) in different segmentations by restricting it to a total genomic coverage of 2% (100 samples of random subsetting) to control for different number of enhancer calls between the segmentations. Error bars show the 95% confidence interval. ChromHMM-15 is omitted because it only has one intergenic enhancer state.
(PDF)

S17 Fig Precision and recall of GWAS SNPs for promoter and enhancer 200bp bins. (A) Median SNP recall and frequency was calculated for enhancer states in different segmentations by restricting it to a total genomic coverage of 2% (100 samples of random subsetting) to control for different number of enhancer calls between the segmentations. Error bars show the 95% confidence interval. (B) The same as in (A) but for promoters.
(PDF)

S18 Fig Dependency of number of predicted promoters and enhancers on tissue group and sample type. (A) Number of enhancer states per Roadmap Epigenomics cell/tissue group. (B) The same as in (A) for promoters. (C) Number of enhancer states per Roadmap Epigenomics sample type. (D) The same as in (C) for promoters.
(PDF)

S1 Appendix Additional information. This document contains the preprocessing steps of dataset 1 for ChromHMM, a detailed description of the GenoSTAN state annotation on dataset 1 and the author contributions.
(PDF)

S1 Table Number of promoter and enhancer states for the chromatin state annotations analyzed in this study. (PDF)

S2 Table Promoter and enhancers states used to calculate recall of FANTOM5 promoters and enhancers. Two promoter and enhancer states were used for each segmentation, except for the EpicSeg segmentation, which only fitted one enhancer state.
(PDF)
