# Supplementary figures and images for: Accurate Promoter and Enhancer Identification in 127 ENCODE and Roadmap Epigenomics Cell Types and Tissues by GenoSTAN
Source: PLoS One. 2017 Jan 5;12(1):e0169249. doi: 10.1371/journal.pone.0169249 (PMC5215863; doi:10.1371/journal.pone.0169249)

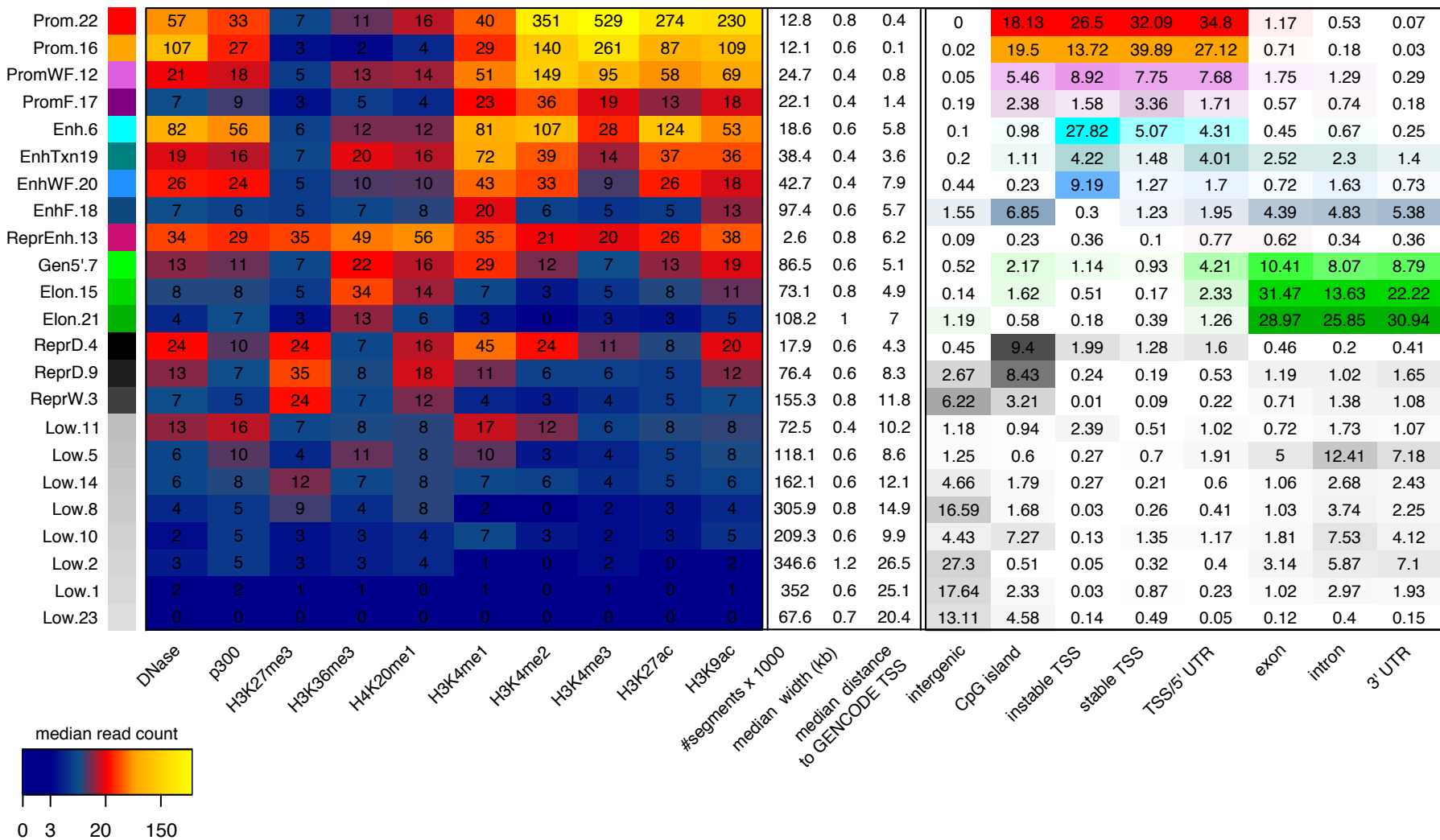

Supplement: S1 Fig — Median read coverage of GenoSTAN-nb-K562 chromatin states (left), their number of annotated segments in the genome, their median width and distance to the closest GENCODE TSS (middle). The right panel shows recall of genomic regions by chromatin states. (PDF) [file pone.0169249.s001.pdf]

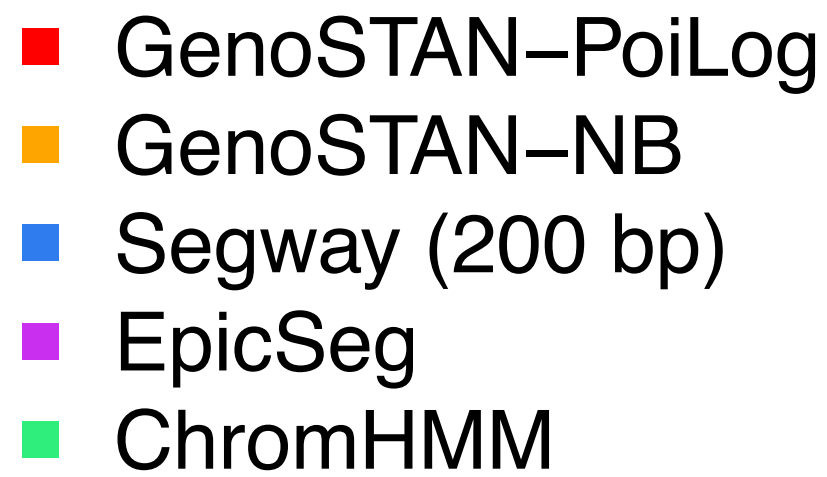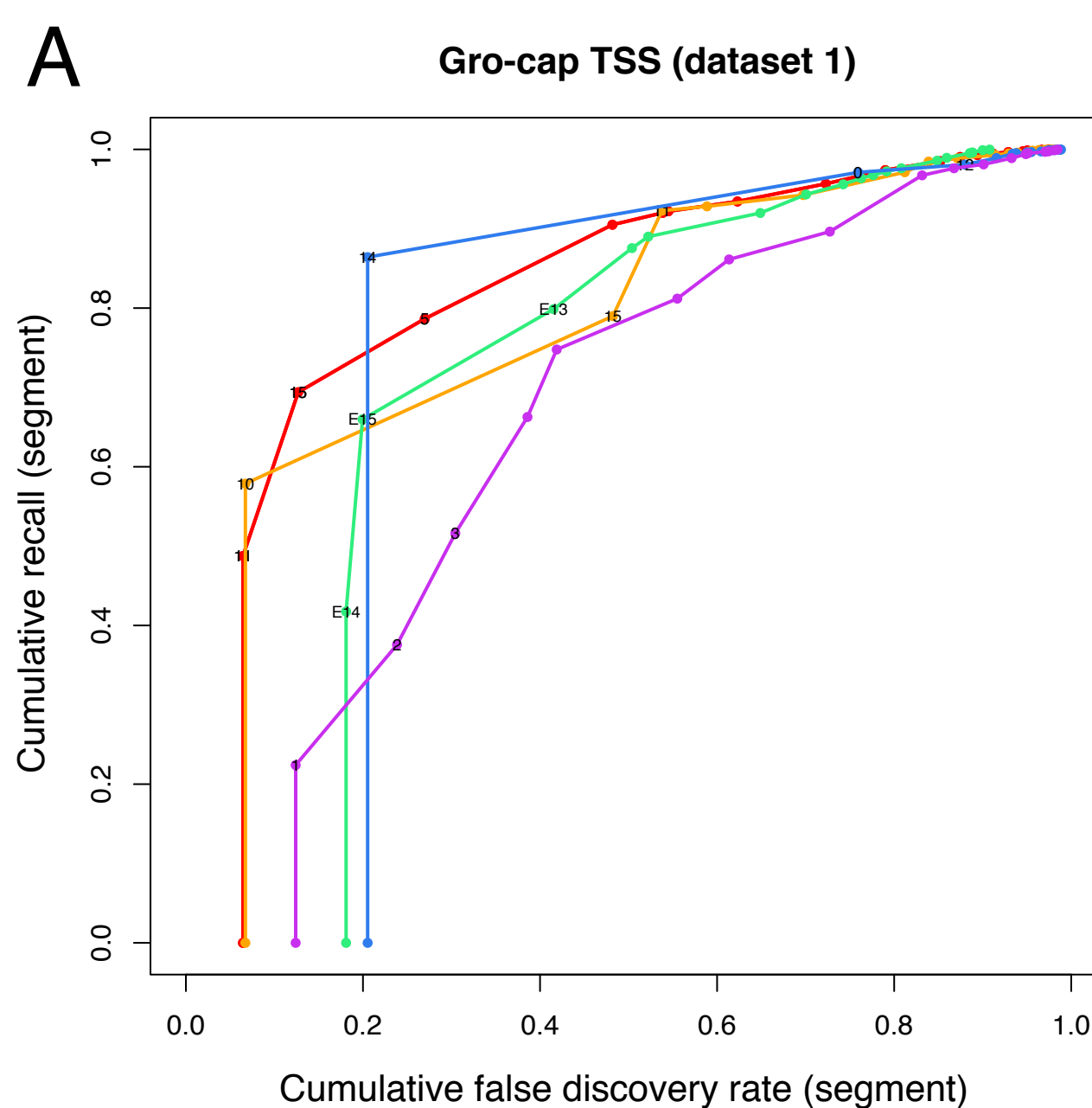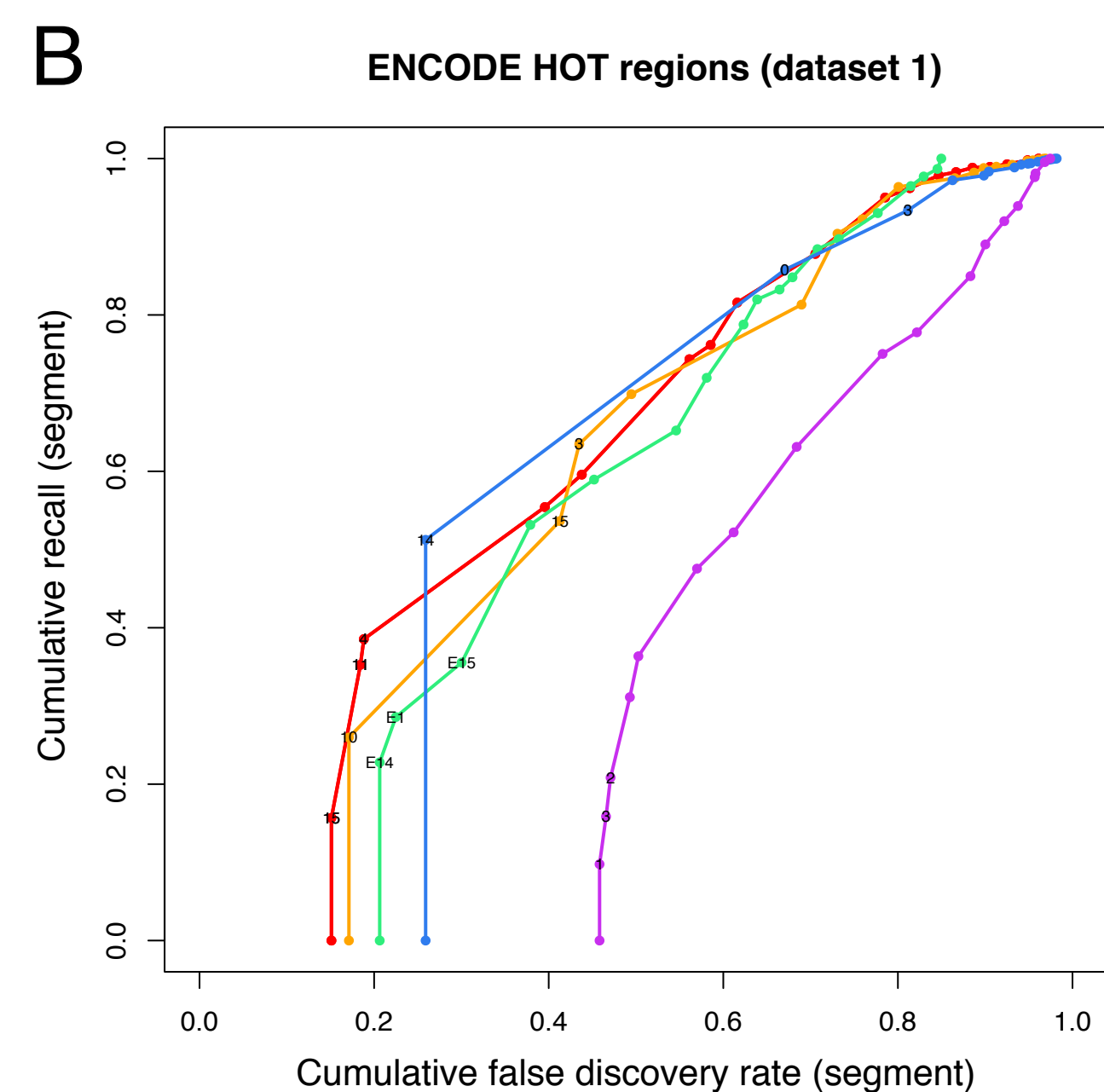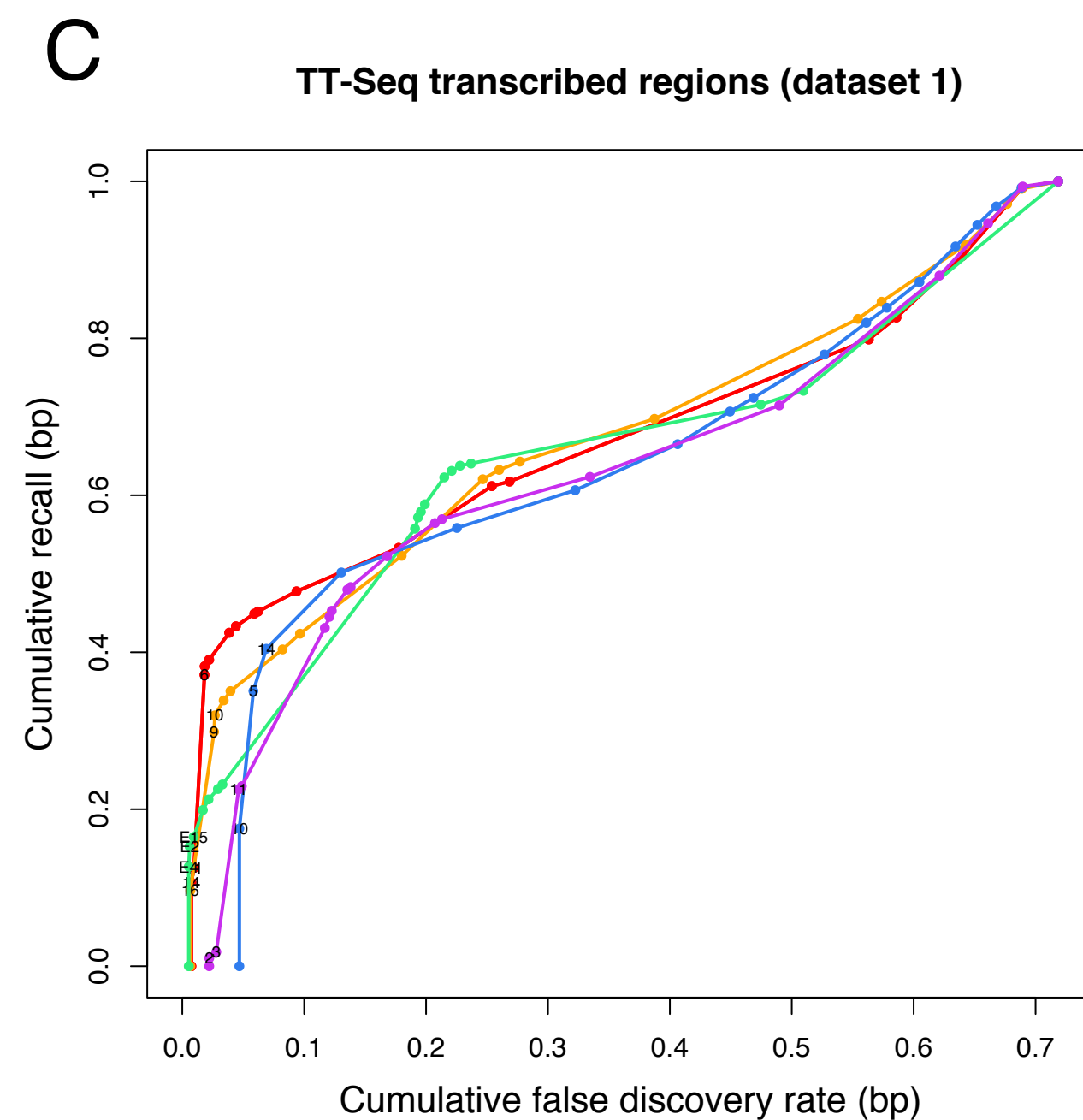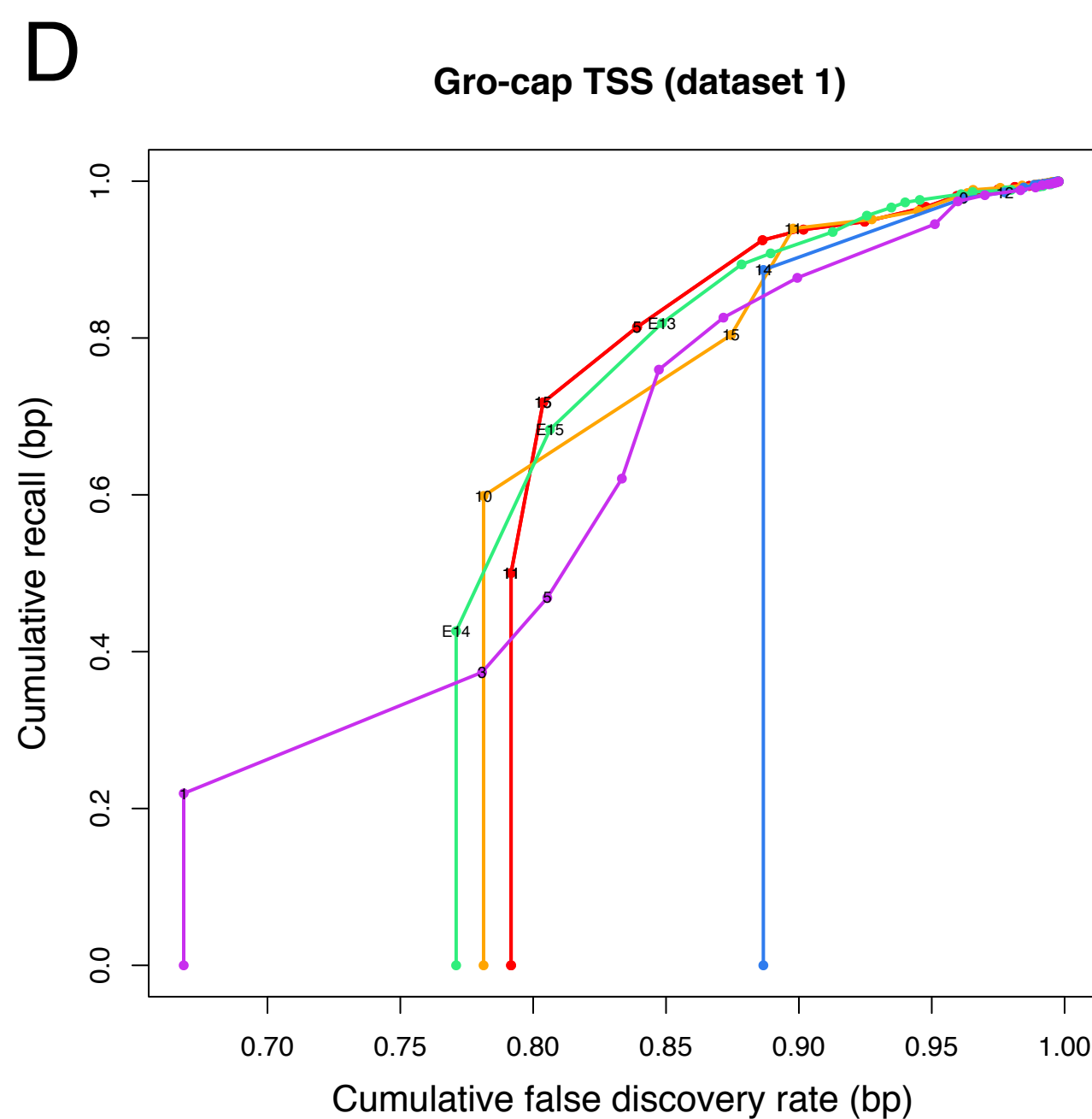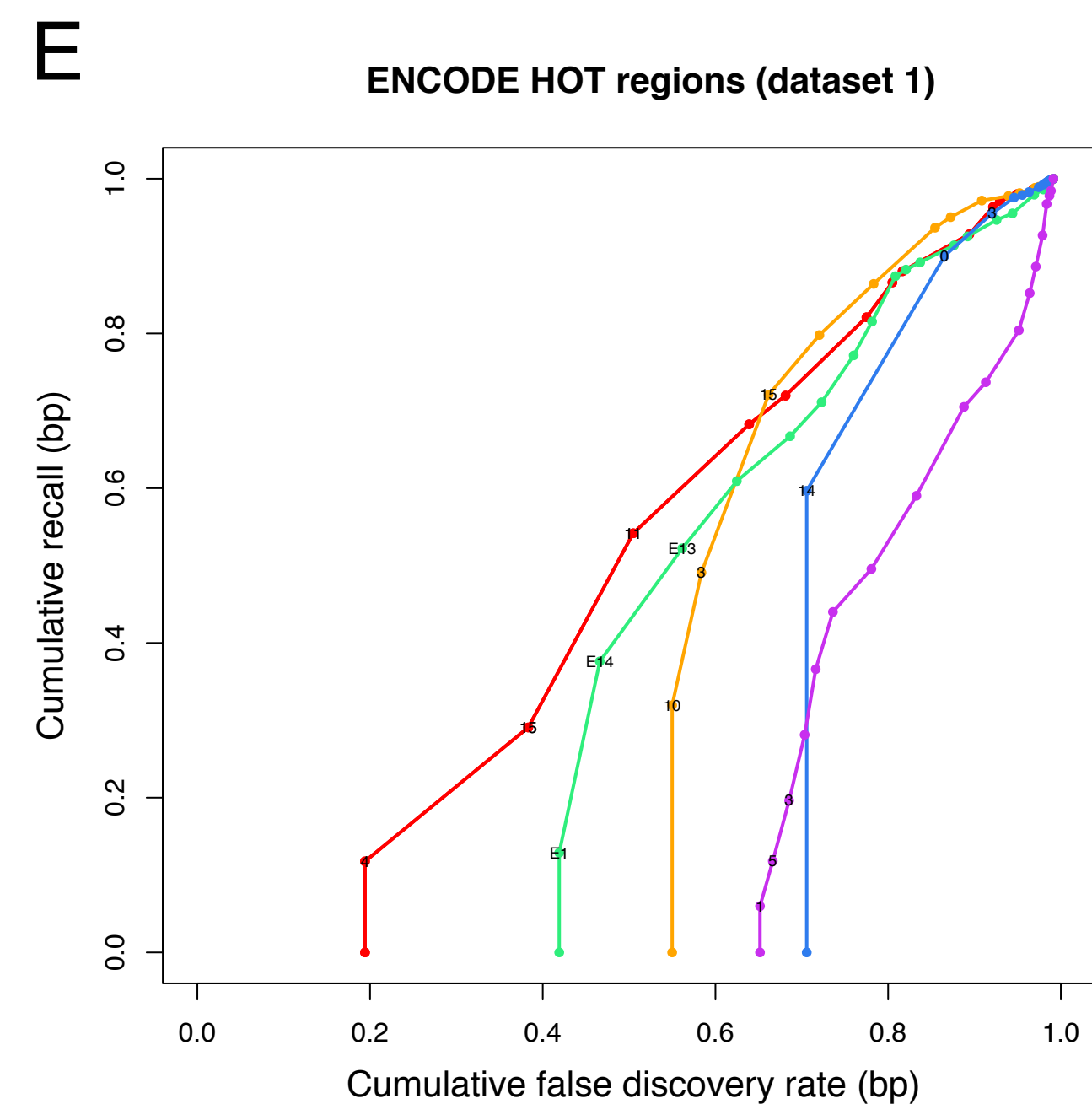

Supplement: S2 Fig — (A) Performance of chromatin states in recovering GRO-cap transcription start sites using state segments. Cumulative FDR and recall are calculated using overlap with state segments by subsequently adding states (in order of increasing FDR). (B) The same as in (A) for ENCODE HOT regions. (C) TT-Seq transcribed regions were overlapped with state annotations on bp level and cumulative FDR and recall were calculated. (D,F) Performance of chromatin states in recovering GRO-cap transcription start sites and ENCODE HOT regions using bp overlap. (PDF) [file pone.0169249.s002.pdf]

■ GenoSTAN-PoiLog  
■ GenoSTAN-NB  
■ EpicSeg

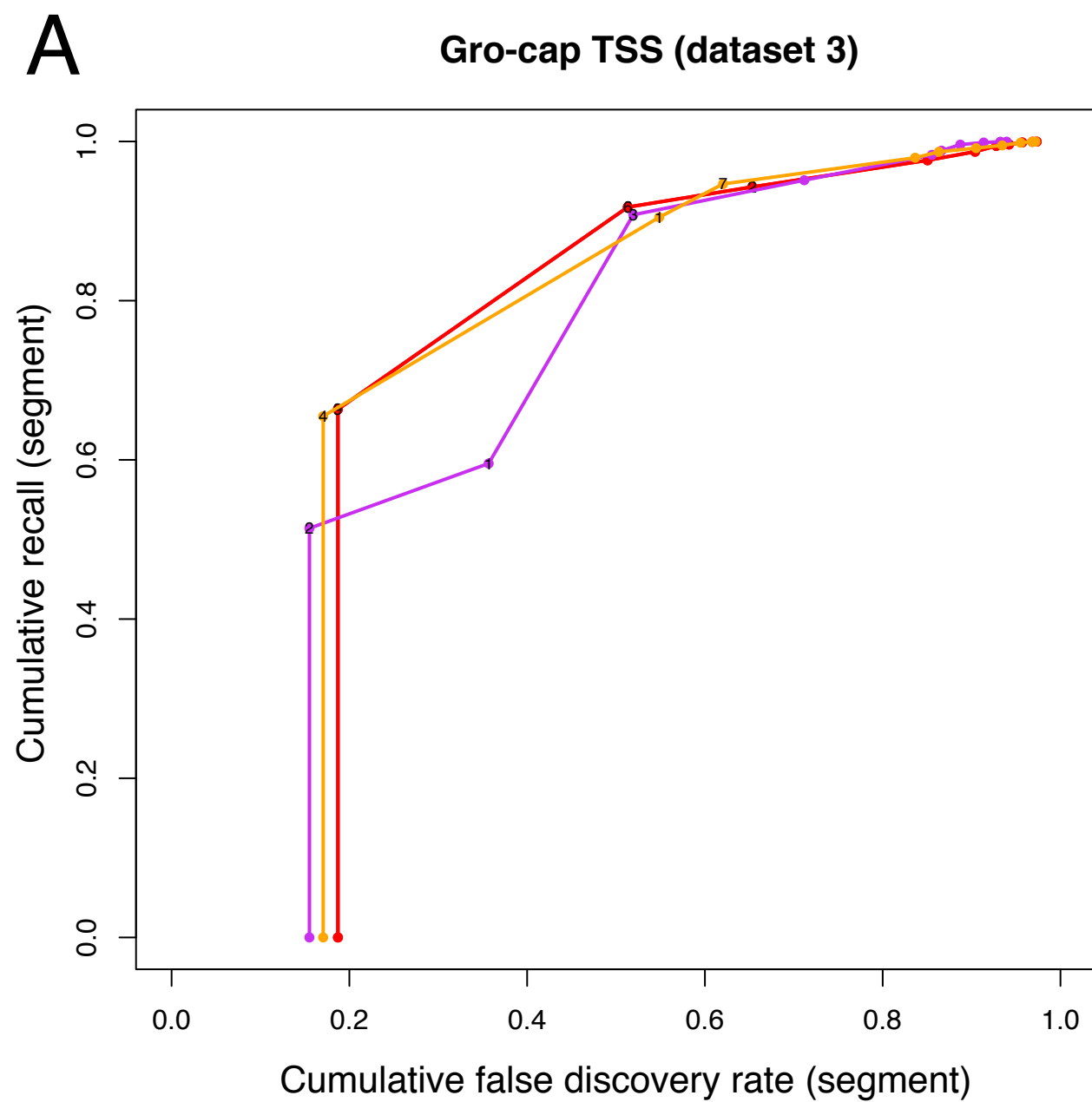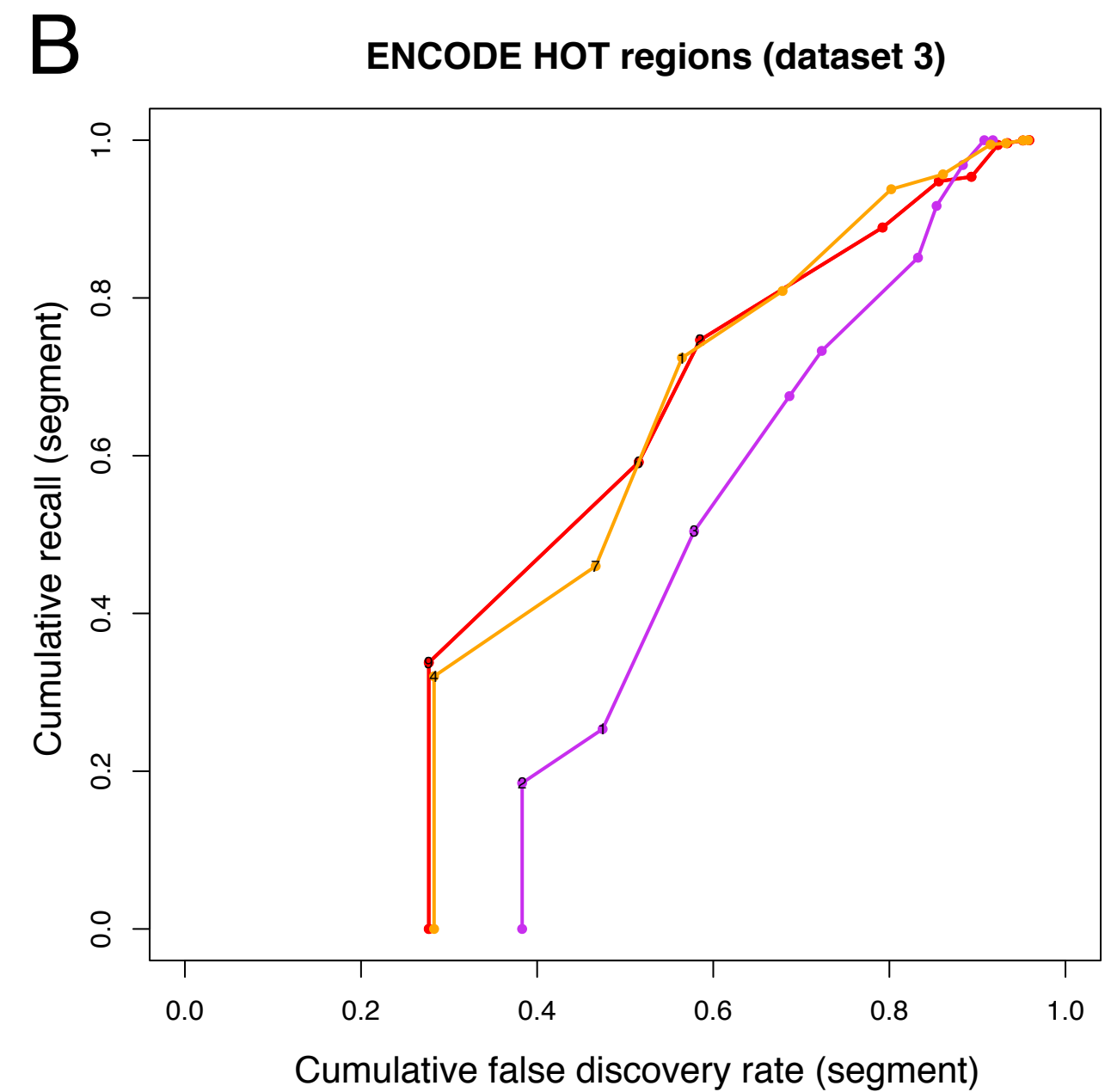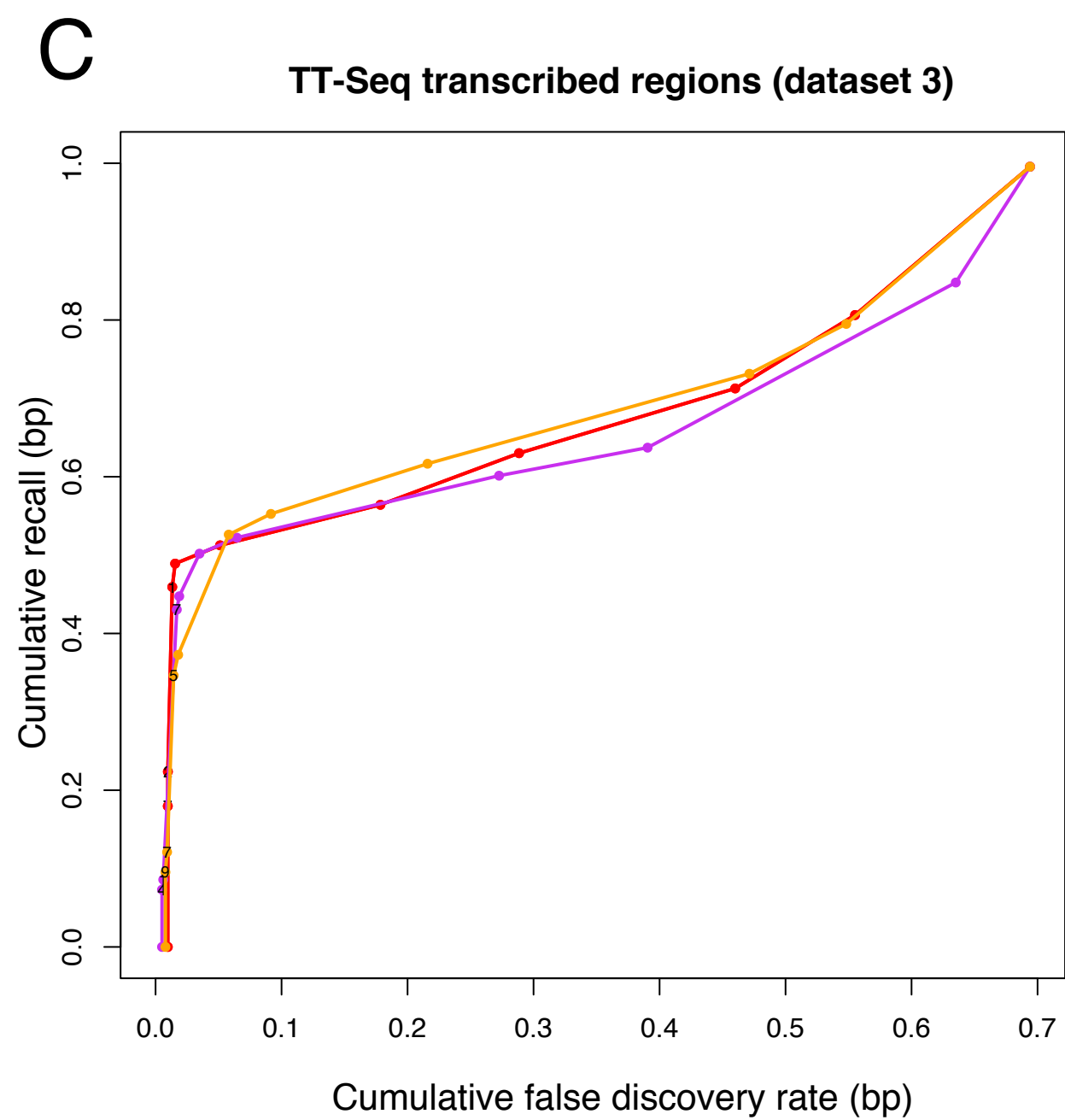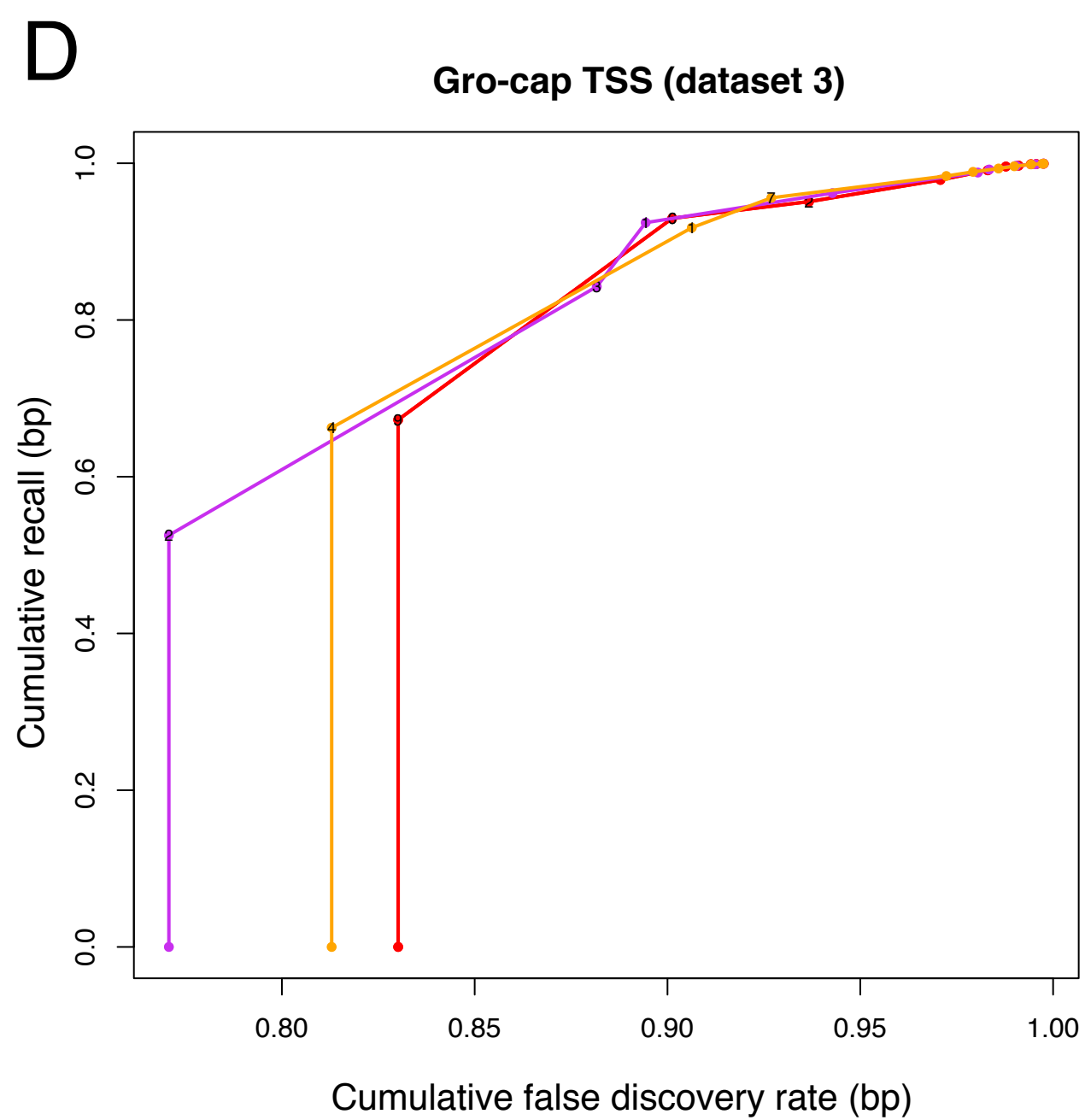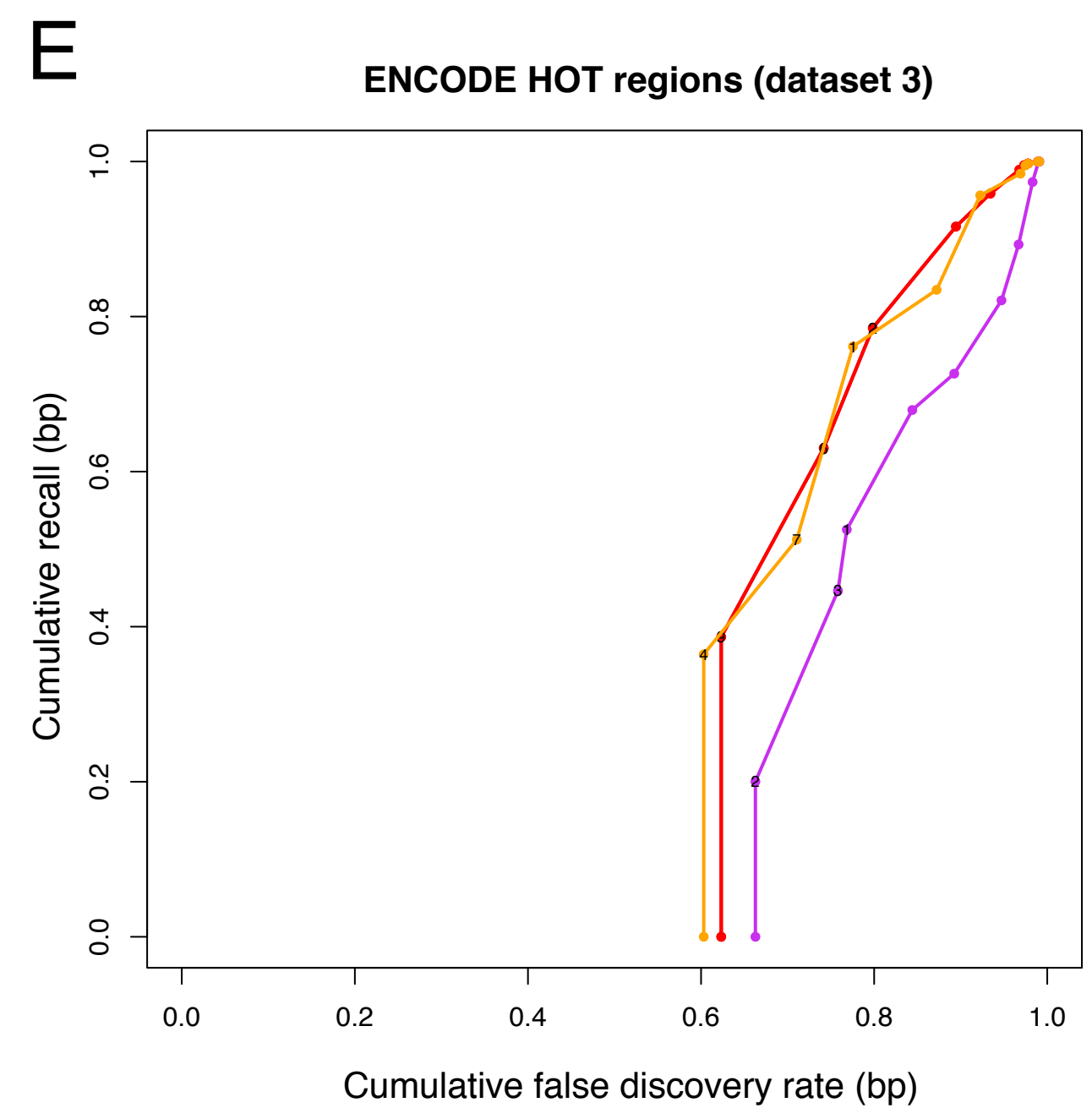

Supplement: S4 Fig — (A) Performance of chromatin states in recovering GRO-cap transcription start sites using state segments. Cumulative FDR and recall are calculated using overlap with state segments by subsequently adding states (in order of increasing FDR). (B) The same as in (A) for ENCODE HOT regions. (C) TT-Seq transcribed regions were overlapped with state annotations on bp level and cumulative FDR and recall were calculated. (D,F) Performance of chromatin states in recovering GRO-cap transcription start sites and ENCODE HOT regions using bp overlap. (PDF) [file pone.0169249.s004.pdf]

A

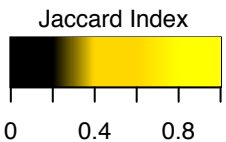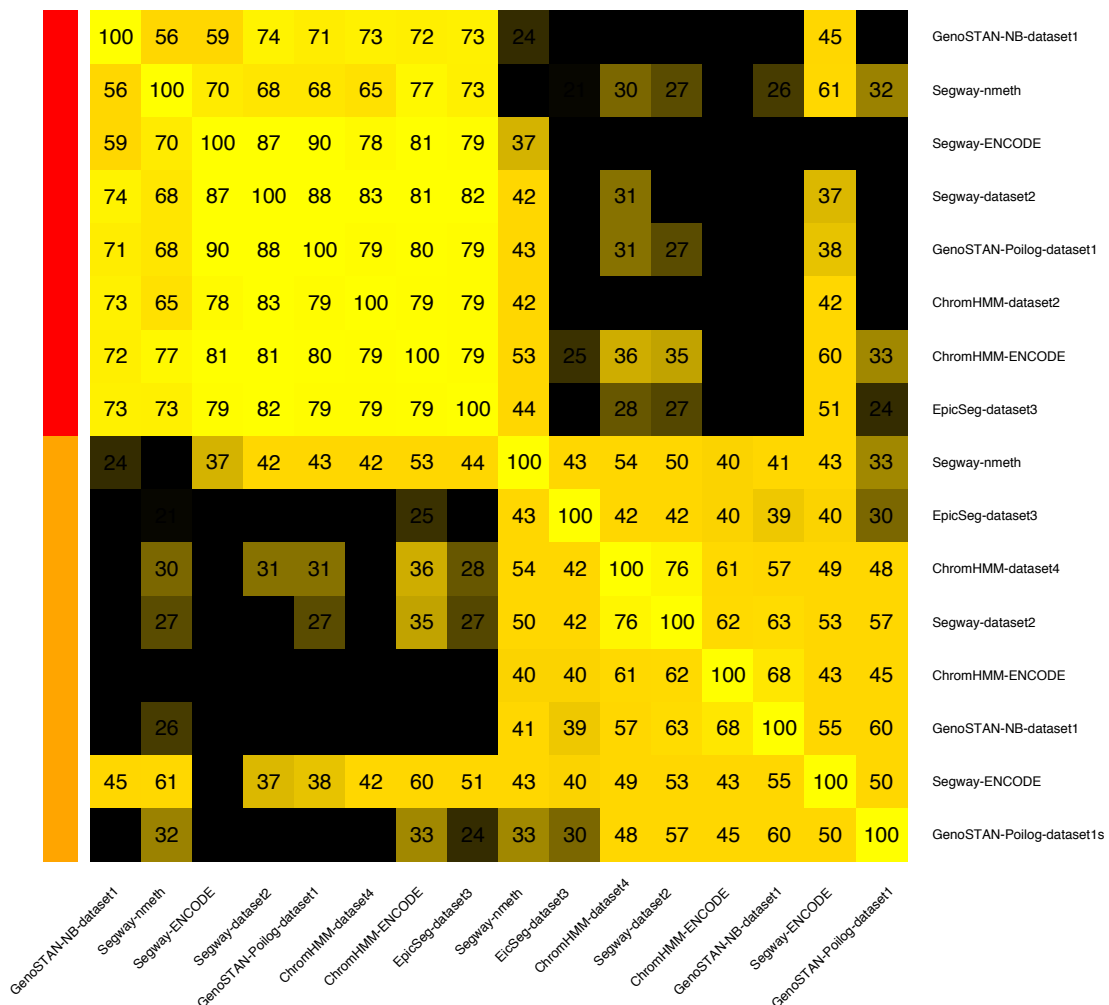

B

Overlap between state annotations (Jaccard Index)

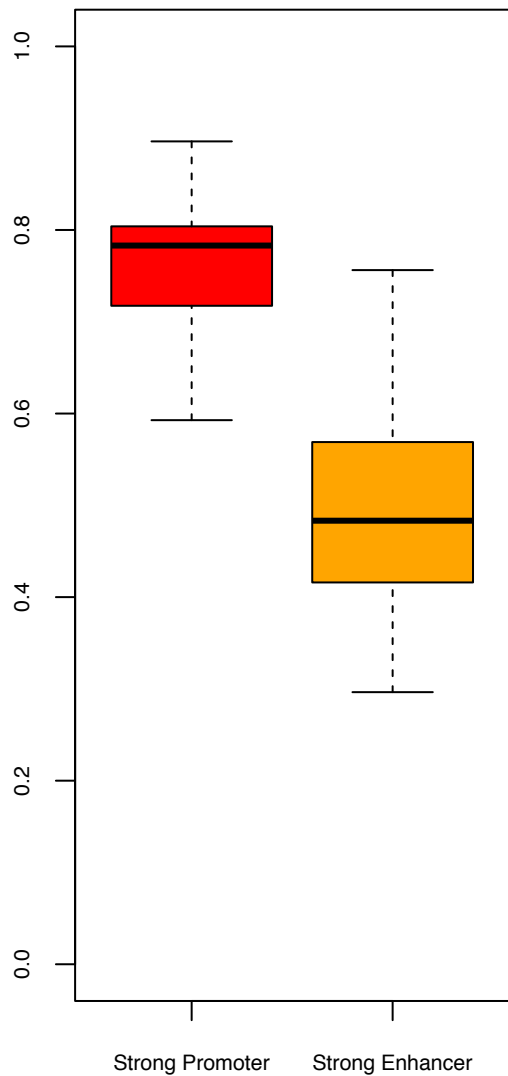

Supplement: S7 Fig — (A) Heatmap of pairwise overlap (Jaccard index) of promoter (red) and enhancer (orange) state annotations from different studies. Rows and columns were ordered by separate clustering of promoter and enhancer overlaps. (B) Distribution of pairwise Jaccard indices for strong promoters and enhancers (off-diagonal elements of promoter and enhancer sub-matrices from (A)). (PDF) [file pone.0169249.s007.pdf]

A

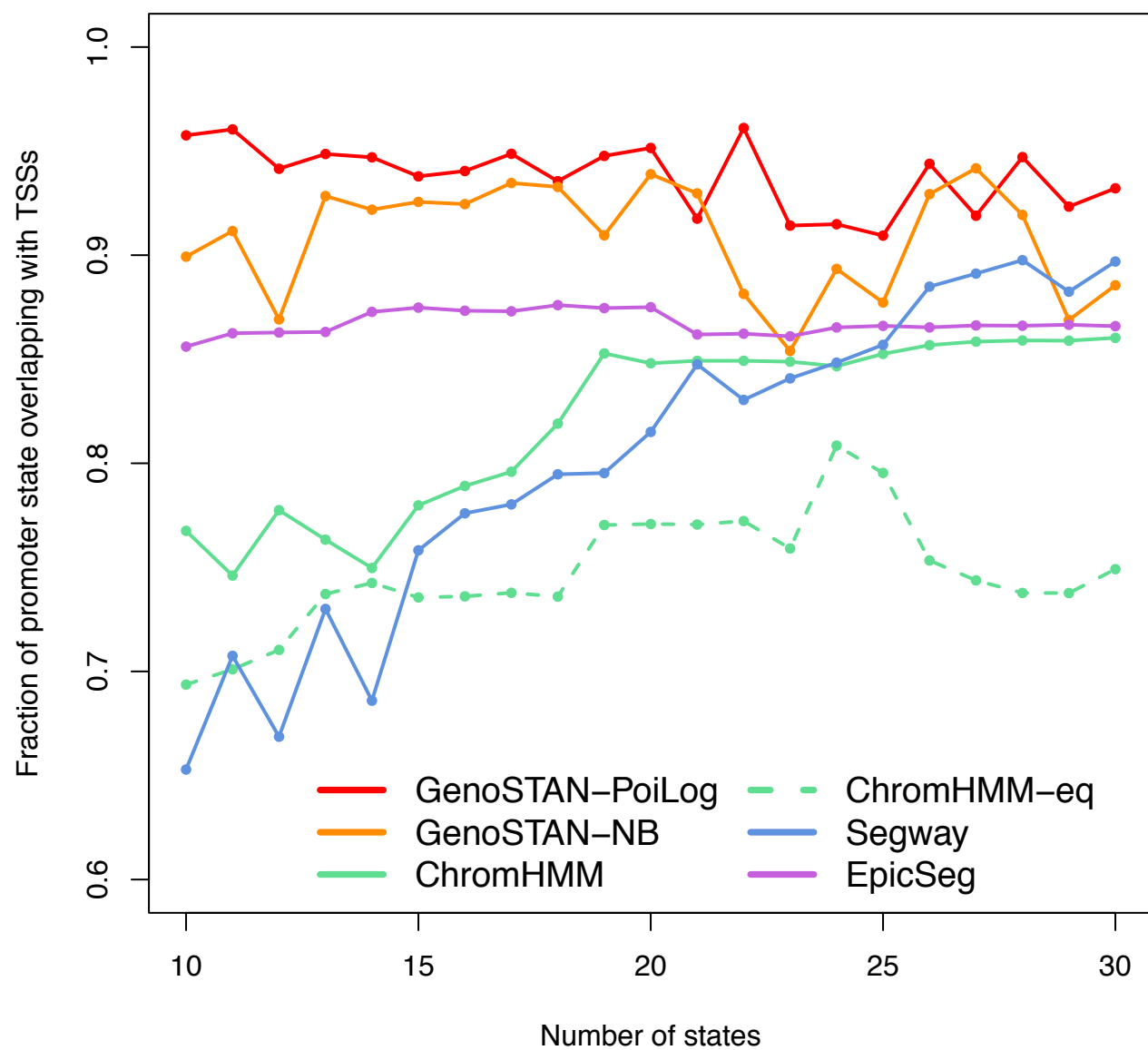

B

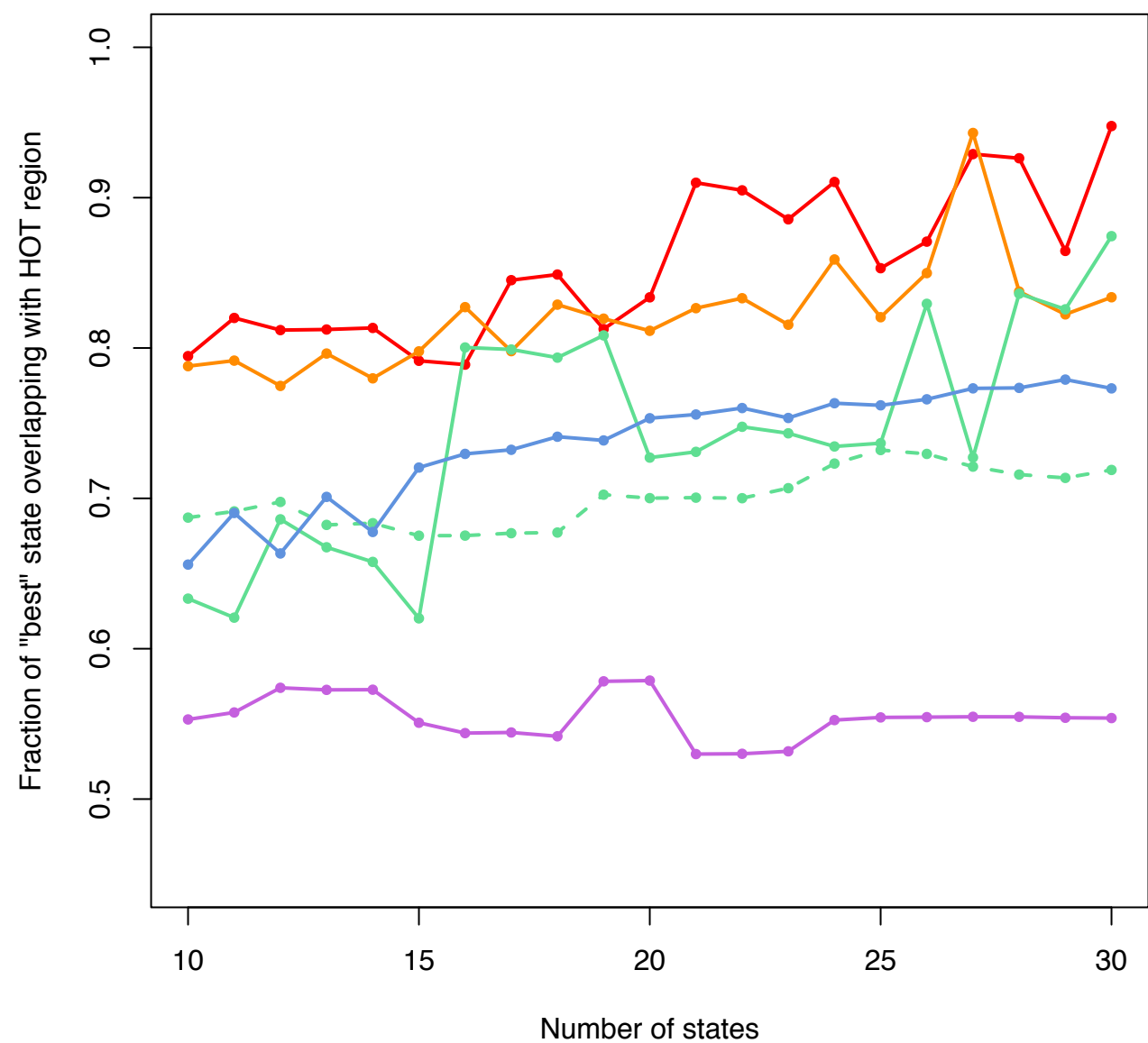

C

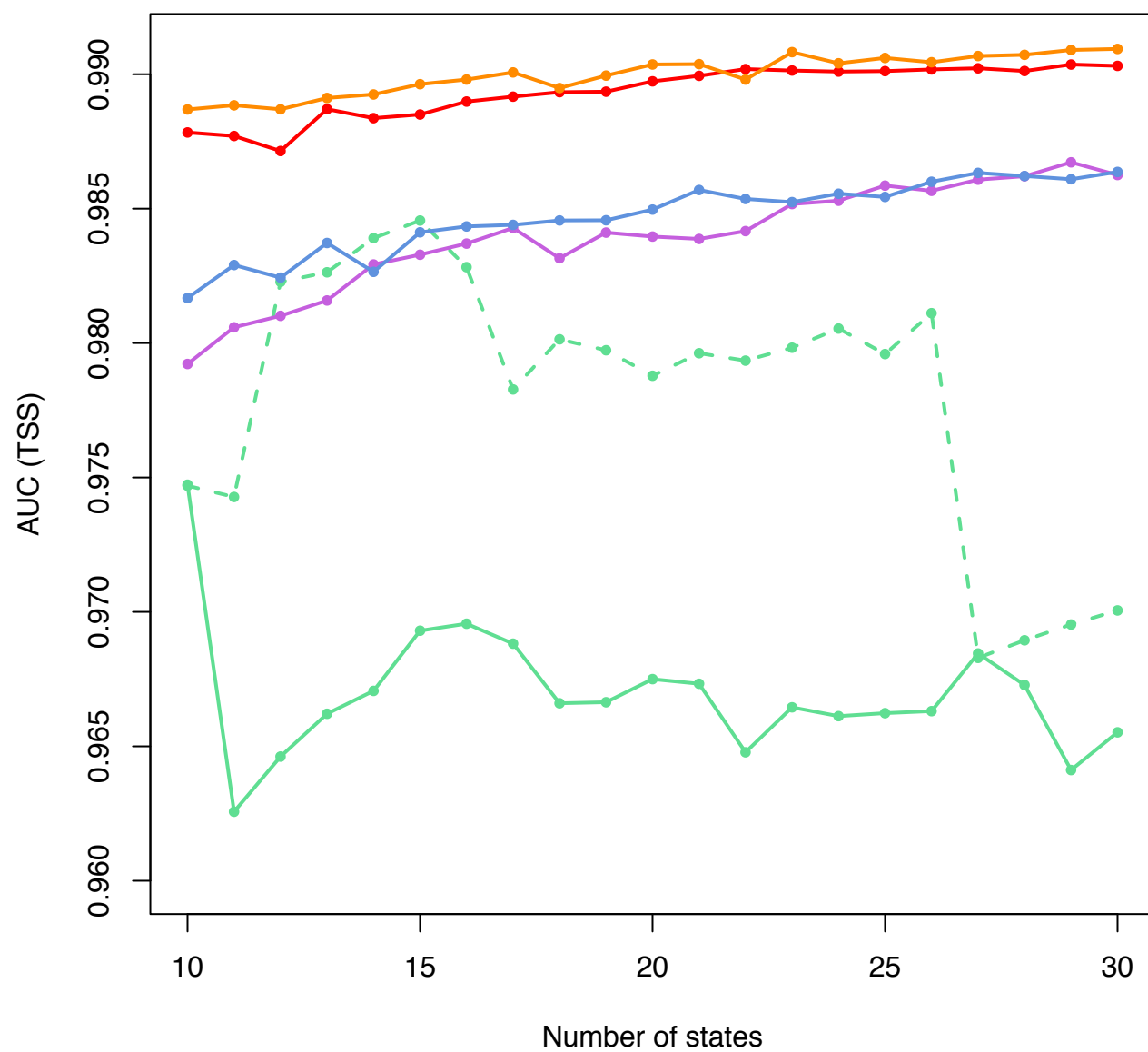

D

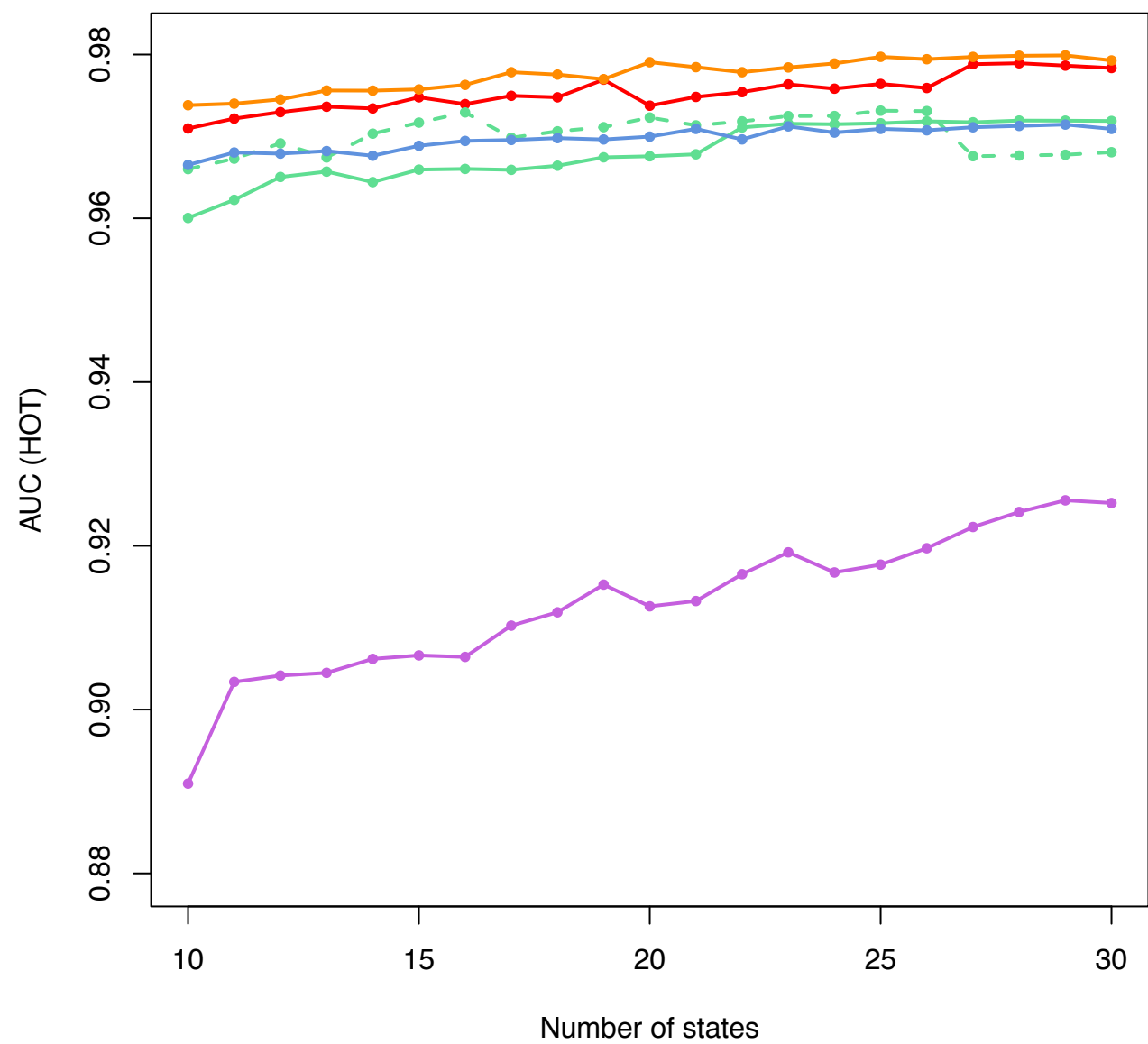

Supplement: S8 Fig — Comparison of chromatin segmentation algorithms with respect to their ability to call GRO-cap transcription start sites (left panels) and ENCODE HOT regions (right panels), as a function of the state number used in the respective algorithm (x-axes). All models were learned on dataset 1. (A-B) For each model, the state with highest precision in recalling HOT (respectively TSS) regions is shown. (C-D) For each model, an area under curve (AUC) score (see Methods) is plotted to asses the spatial accuracy of a genome segmentation. (PDF) [file pone.0169249.s008.pdf]

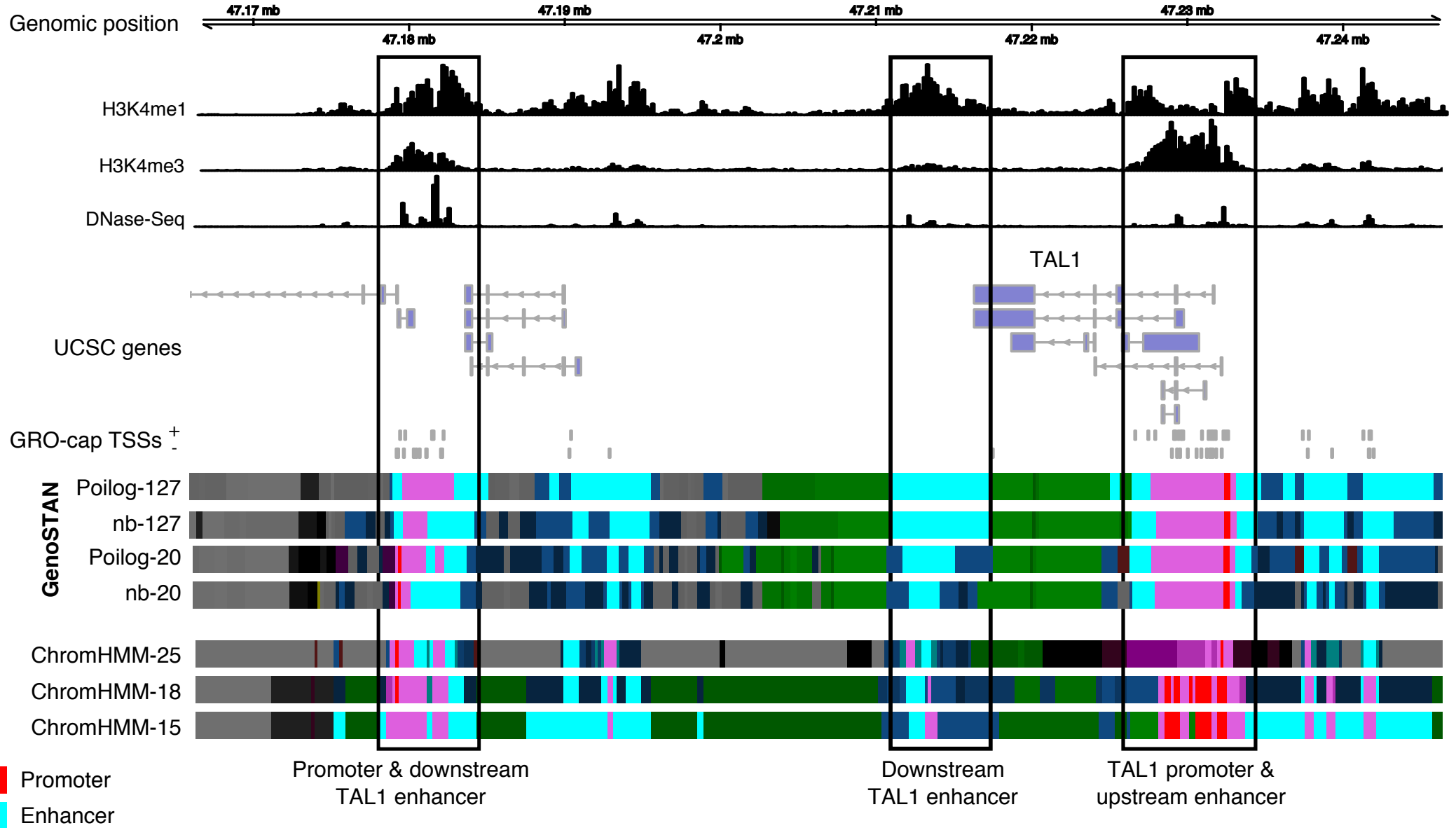

Supplement: S9 Fig — GenoSTAN models fitted on a subset of 20 and all 127 cell types and tissues from Roadmap Epigenomics are compared to ChromHMM models with 15, 18 and 25 states at the TAL1 gene in K562. (PDF) [file pone.0169249.s009.pdf]

A

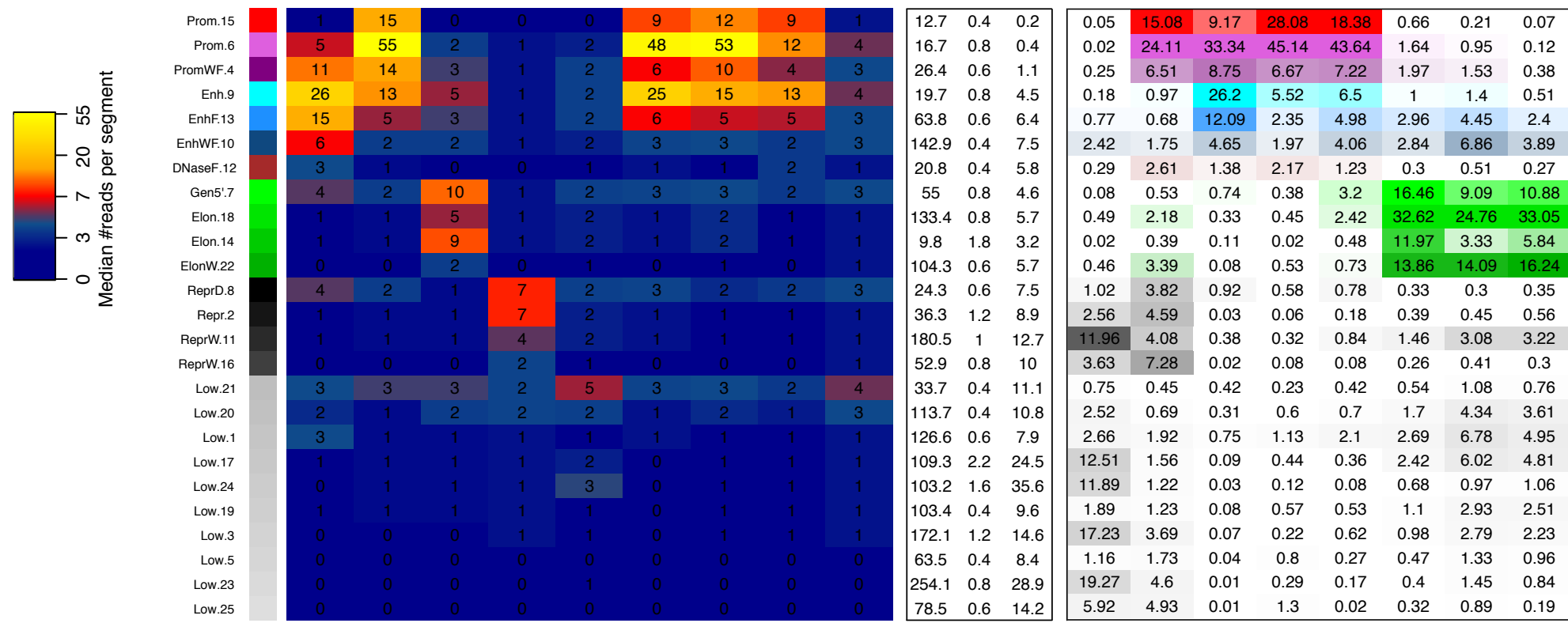

B

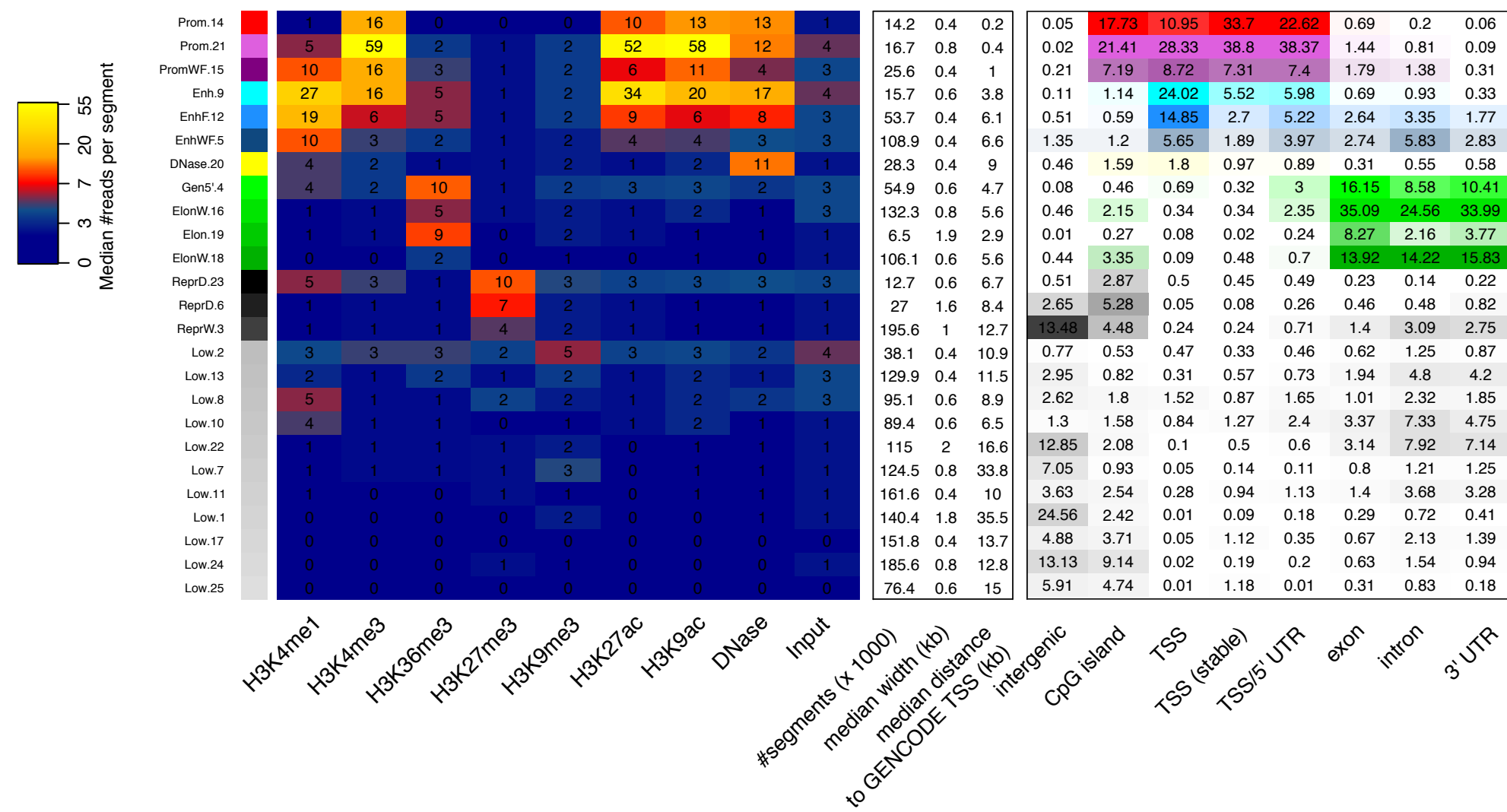

Supplement: S11 Fig — (A) Median read coverage of GenoSTAN-Poilog-20 chromatin states (left), their number of annotated segments in the genome, their median width and distance to the closest GENCODE TSSs of segments (middle). The right panel shows recall of genomic regions by chromatin states. (B) The same as (A) for GenoSTAN-nb-20. (PDF) [file pone.0169249.s011.pdf]

A

GENCODE

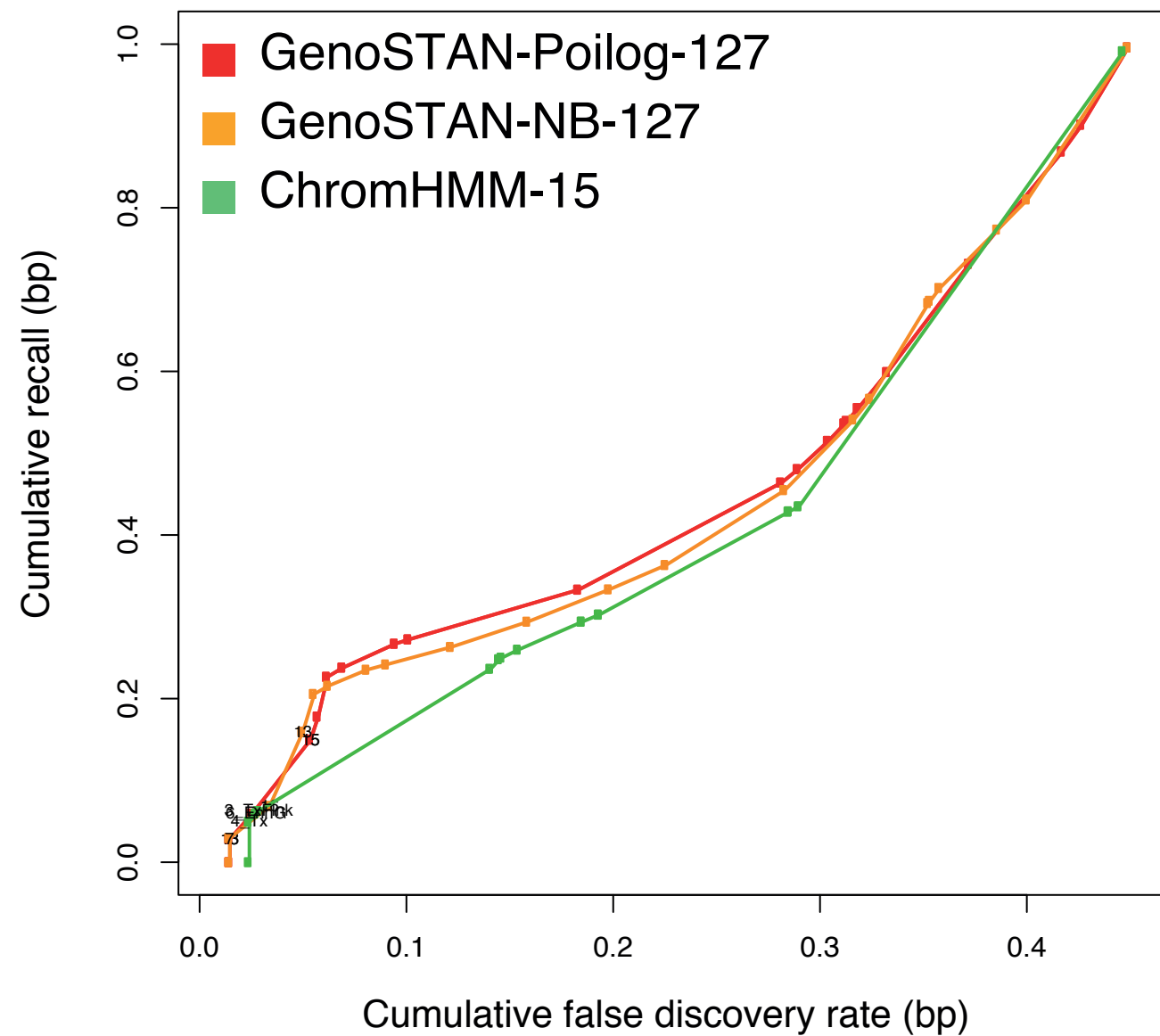

B

TT-Seq transcribed regions

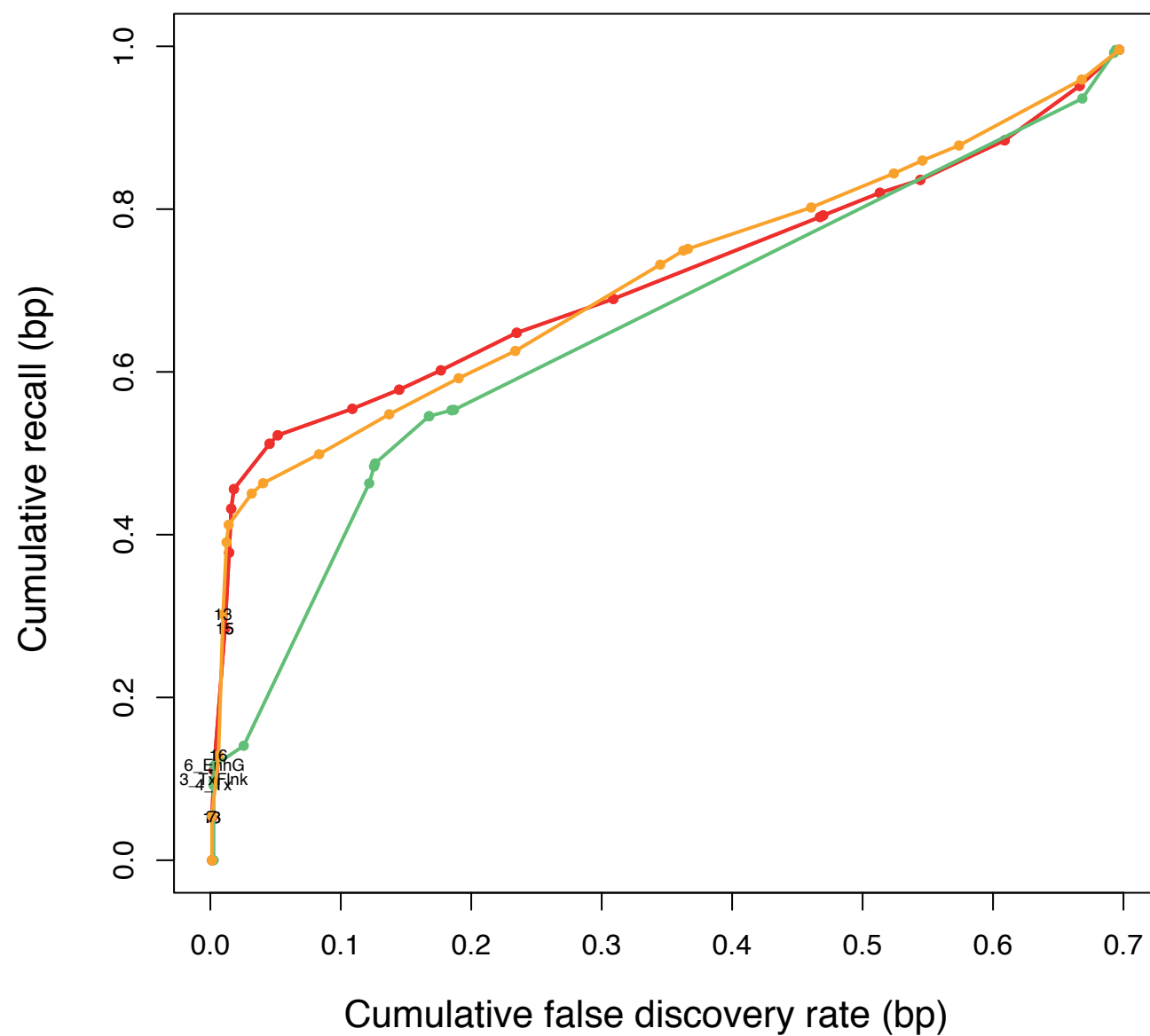

Supplement: S12 Fig — GenoSTAN-(Poilog/NB)-127 is shown in red/orange, ChromHMM-15 in green. All three models were learned on the same data (H3K4me1, H3K4me3, H3K36me3, H3K27me3, H3K9me3 and an input control). GenoSTAN models were learned with 20, ChromHMM-15 with 15 states. In both plots, cumulative FDR and recall are calculated by subsequently adding states (in order of increasing FDR). Performance of chromatin states in recovering GENCODE gene annotations (A) and TT-seq transcribed regions (B) in K562 at bp level. (PDF) [file pone.0169249.s012.pdf]

A

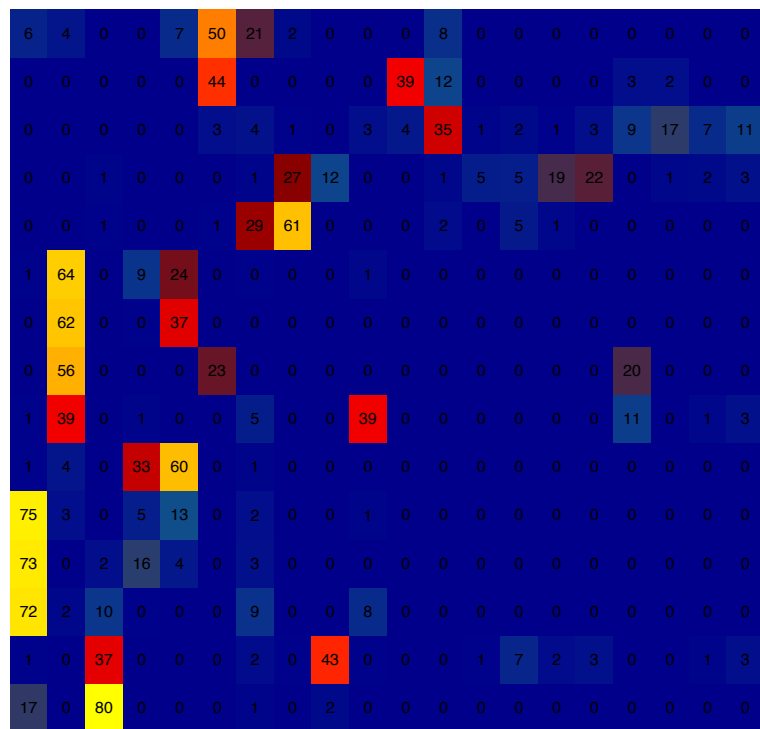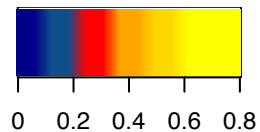

B

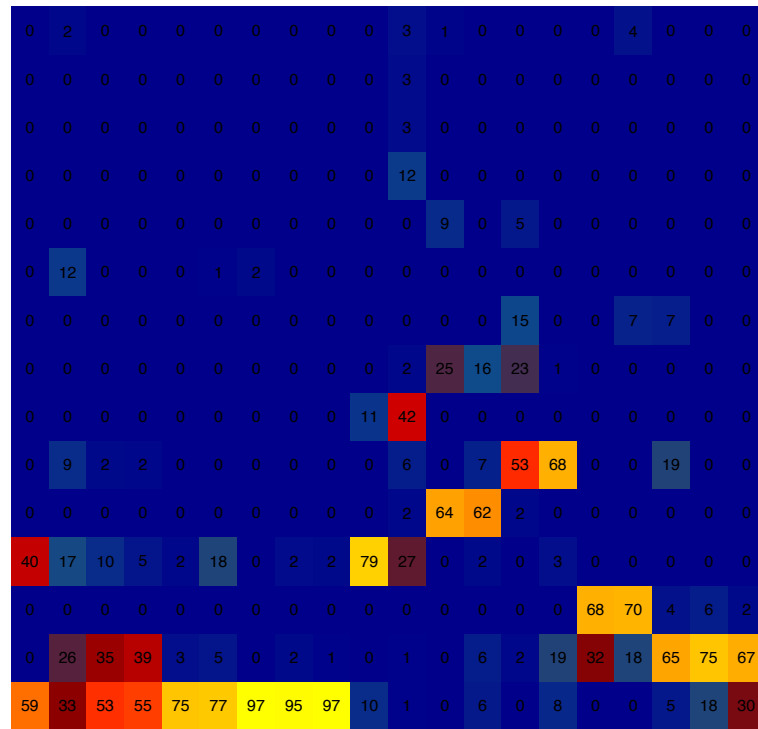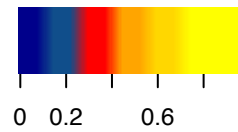

Supplement: S13 Fig — (A) Rows were normalized to sum up to 1. (B) Columns were normalized to sum up to 1. (PDF) [file pone.0169249.s013.pdf]

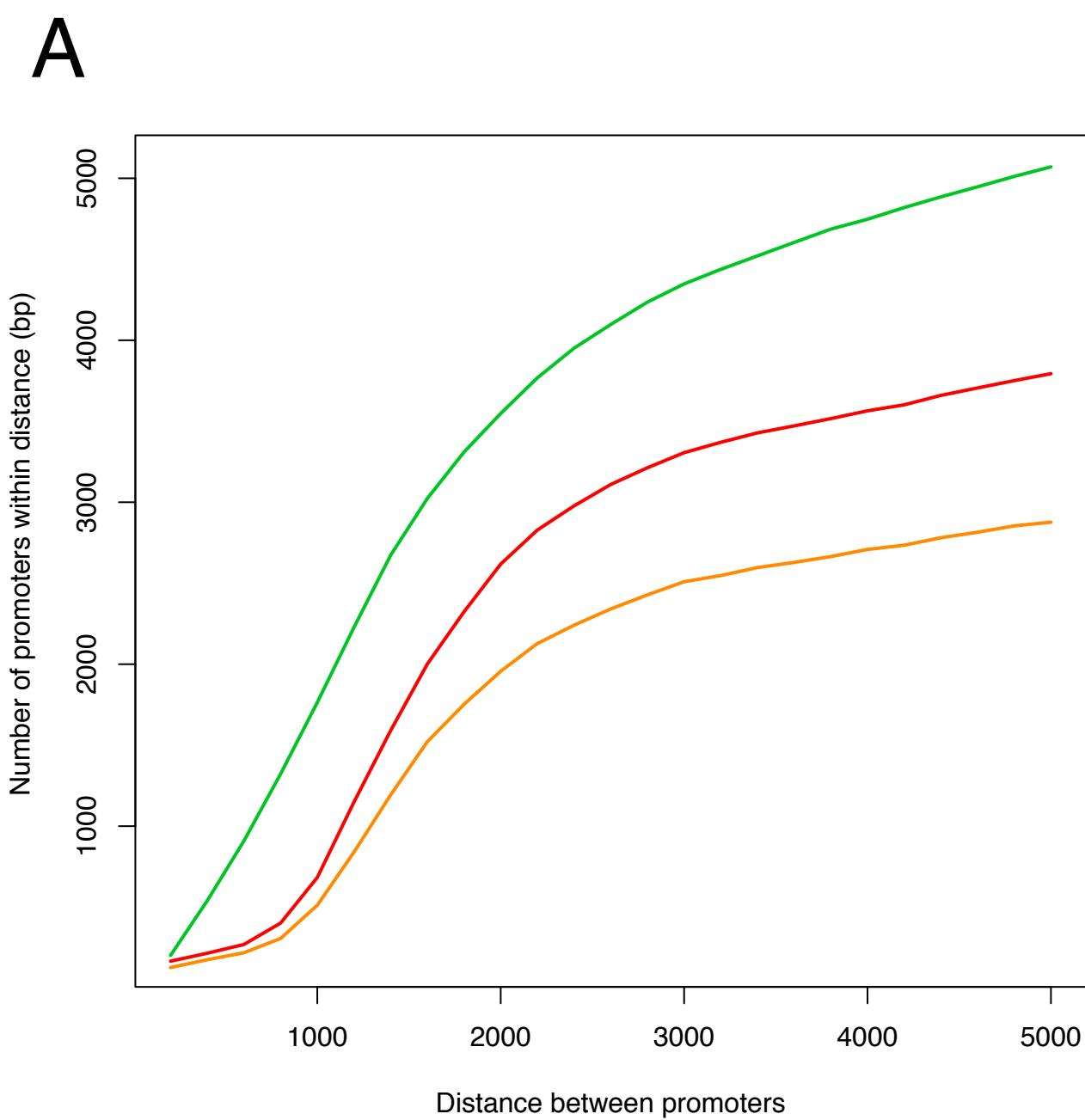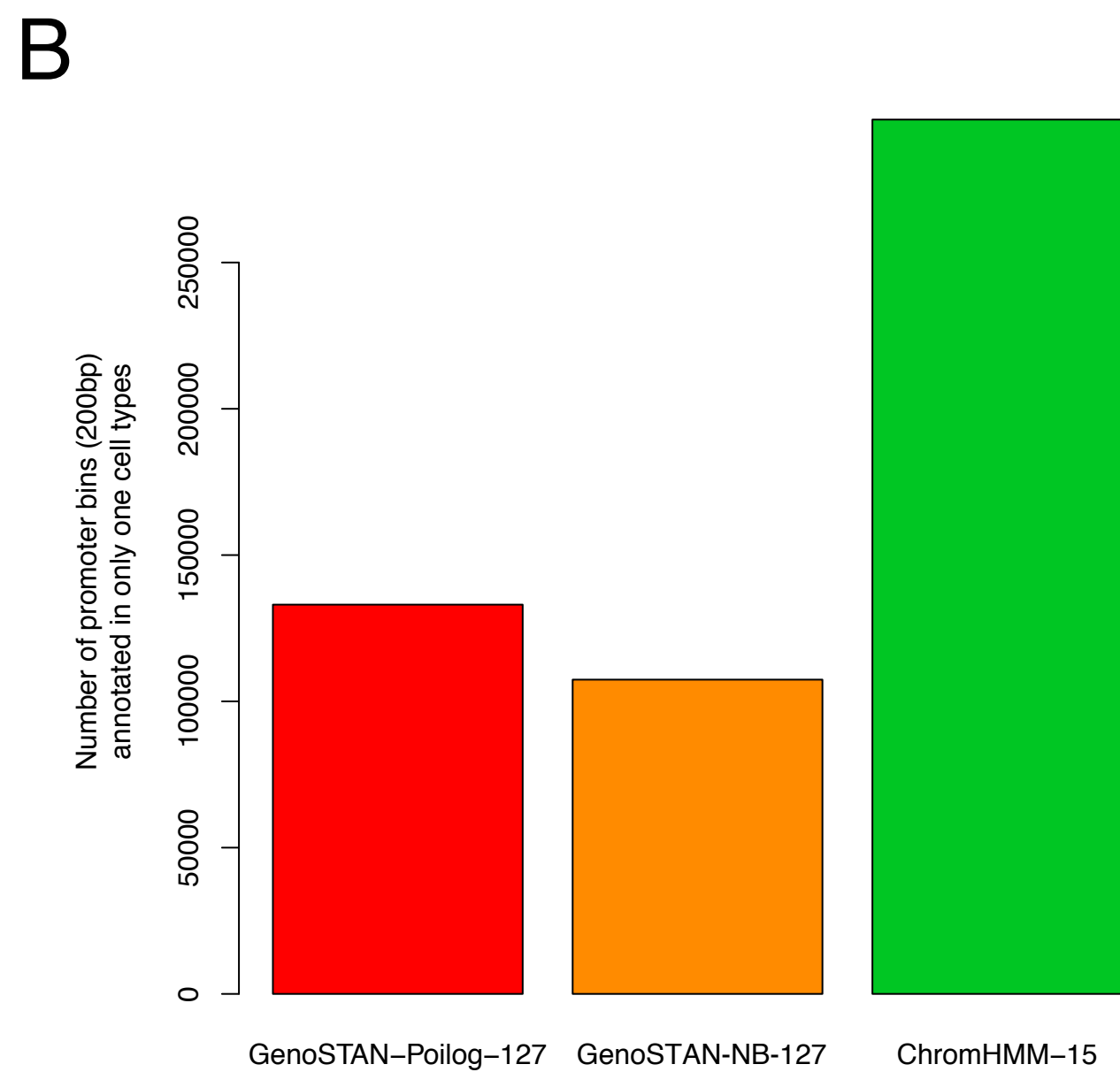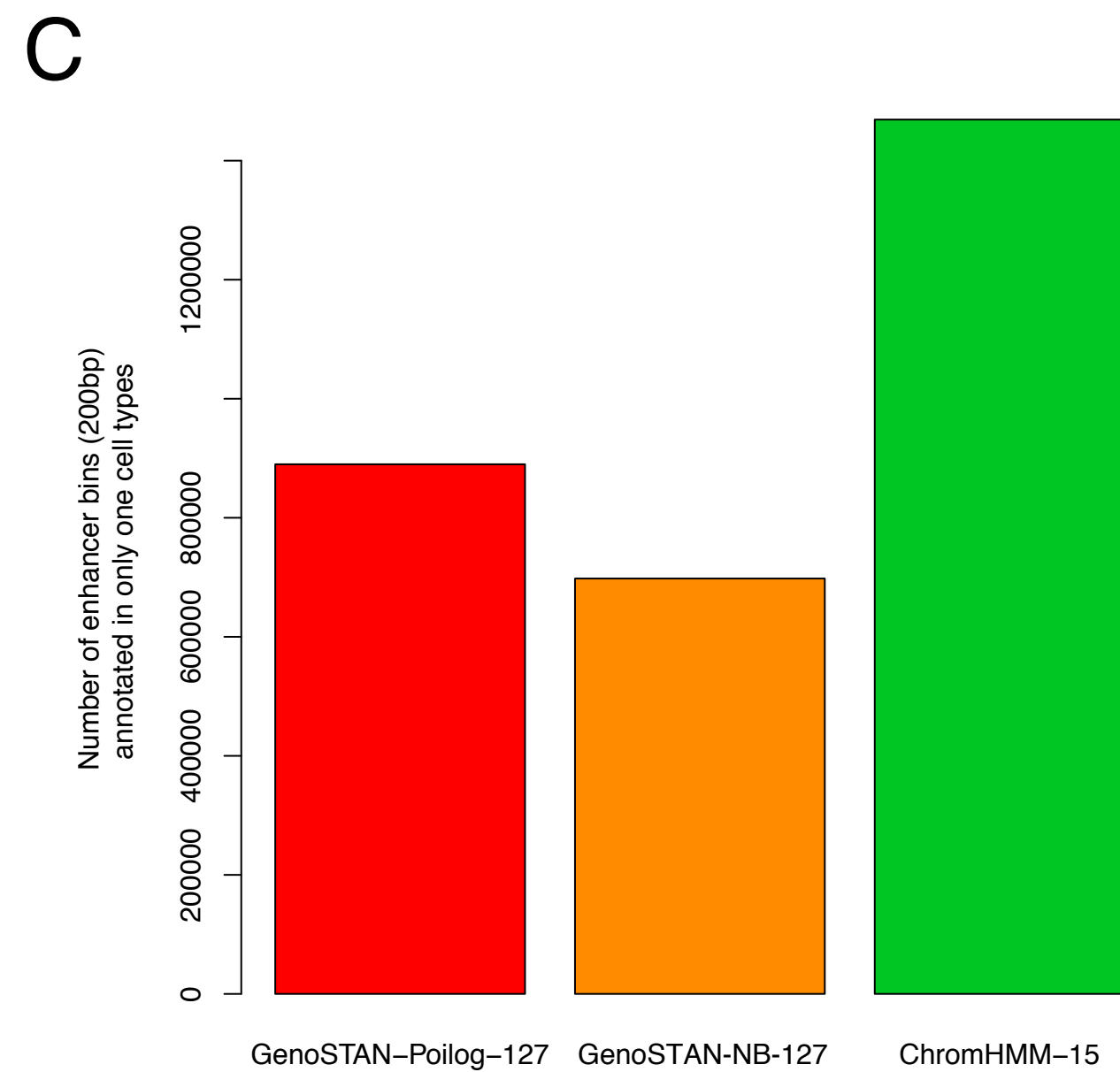

Supplement: S14 Fig — (A) Estimated cumulative distribution of promoter states within a certain distance along genome in K562. The number of 200bp bins that are annotated in only one (out of 127) cell types are counted for promoters (B) and enhancers (C) for the different segmentations. (PDF) [file pone.0169249.s014.pdf]

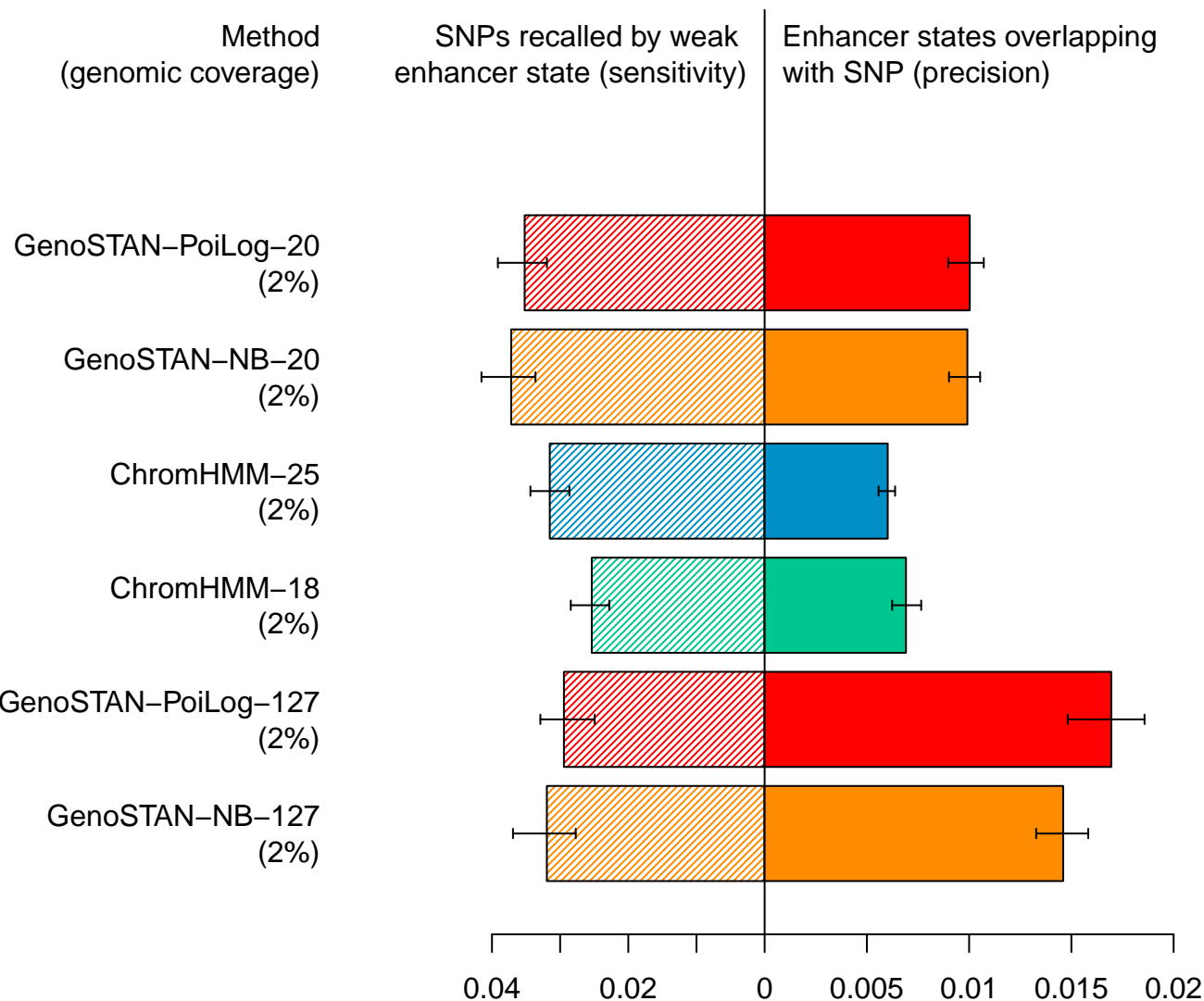

Supplement: S16 Fig — Median SNP recall and frequency was calculated for weak intergenic enhancer or enhancer flanking states (ChromHMM-18: ‘10_EnhA2’, ChromHMM-25: ‘14_EnhA2’, GenoSTAN-Poilog-20: ‘EnhF.13’, GenoSTAN-NB-20: ‘EnhF.12’, GenoSTAN-Poilog-127: ‘EnhW.9’, GenoSTAN-NB-127: ‘EnhW.8’) in different segmentations by restricting it to a total genomic coverage of 2% (100 samples of random subsetting) to control for different number of enhancer calls between the segmentations. Error bars show the 95% confidence interval. ChromHMM-15 is omitted because it only has one intergenic enhancer state. (PDF) [file pone.0169249.s016.pdf]

A

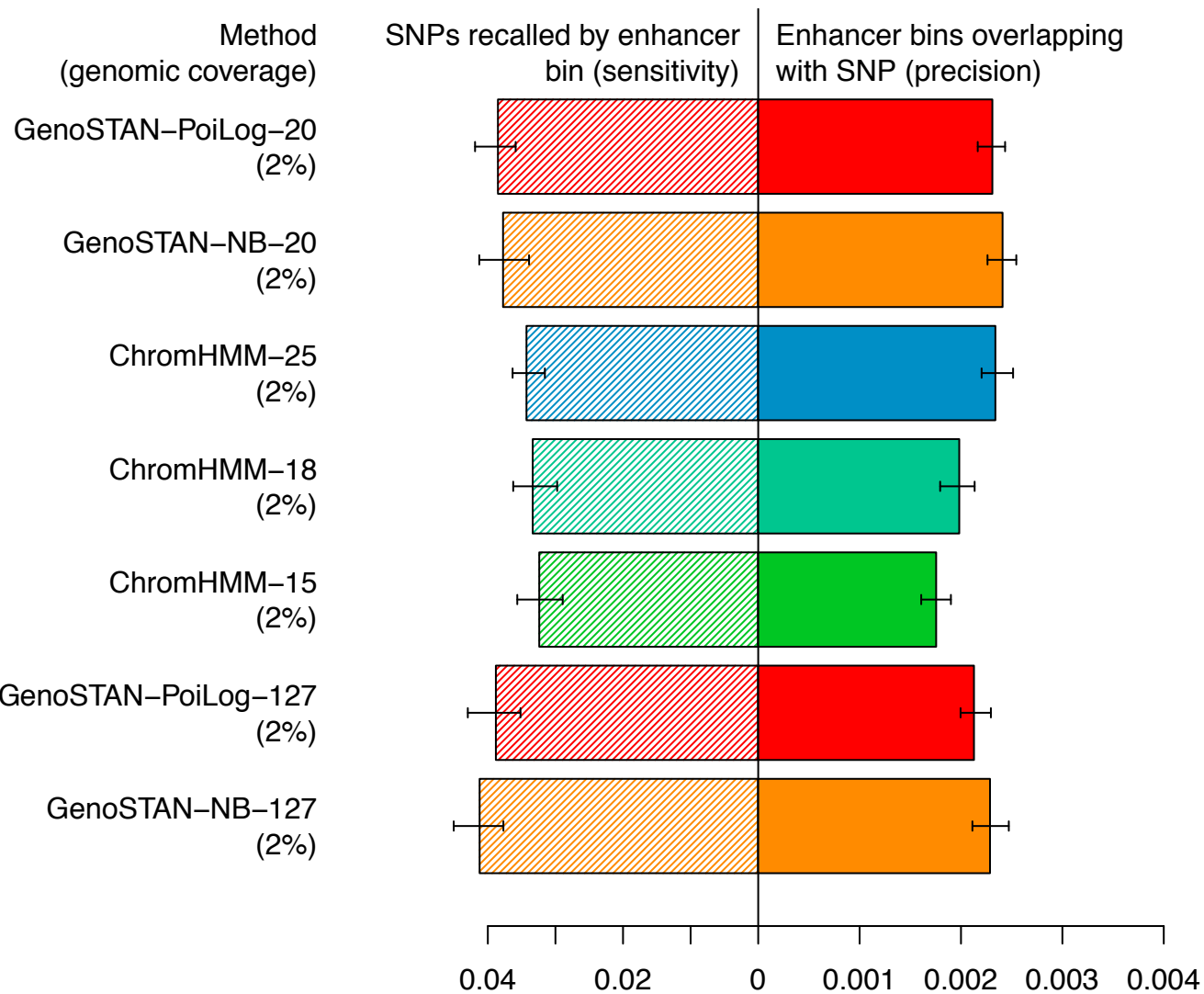

B

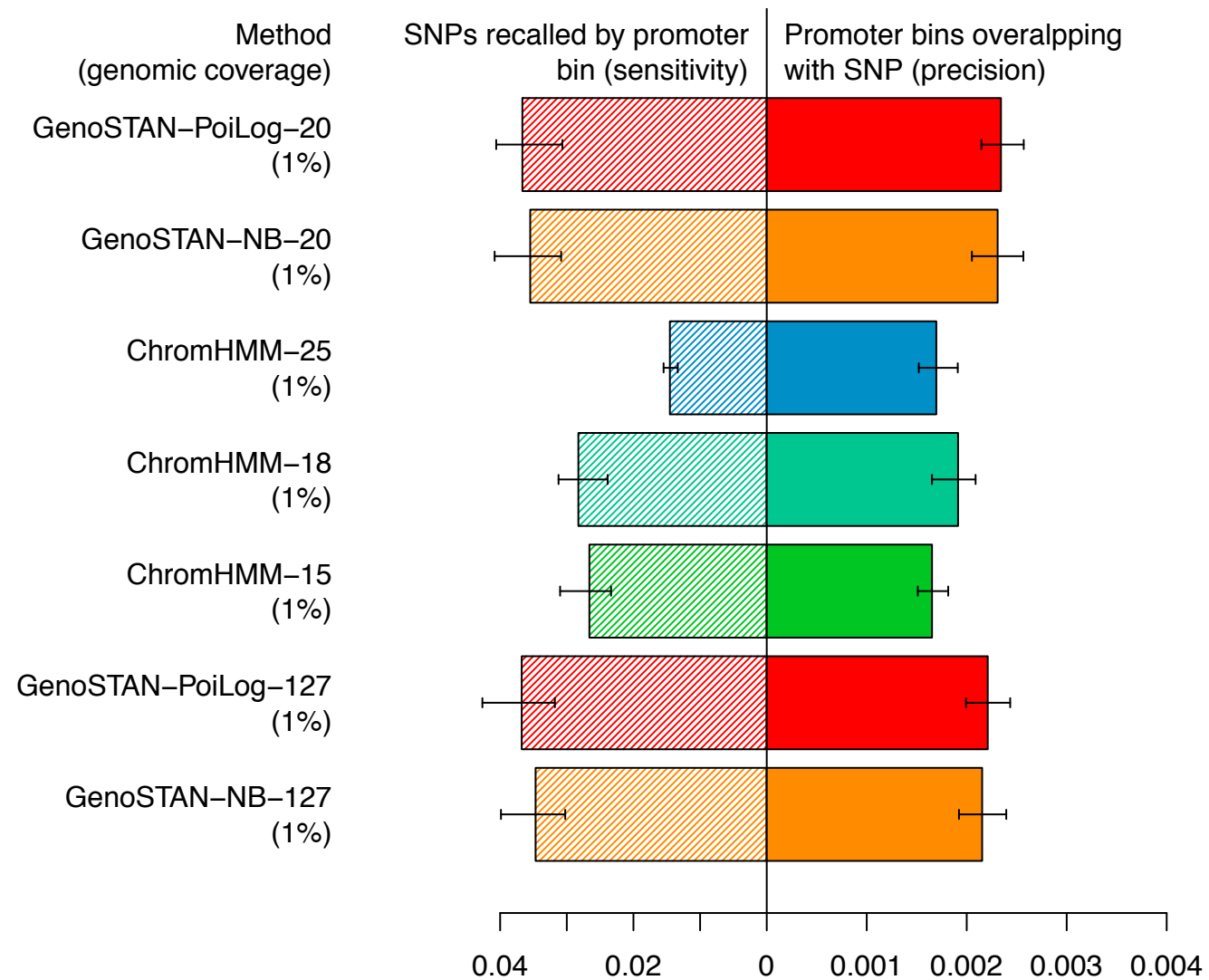

Supplement: S17 Fig — (A) Median SNP recall and frequency was calculated for enhancer states in different segmentations by restricting it to a total genomic coverage of 2% (100 samples of random subsetting) to control for different number of enhancer calls between the segmentations. Error bars show the 95% confidence interval. (B) The same as in (A) but for promoters. (PDF) [file pone.0169249.s017.pdf]

**A**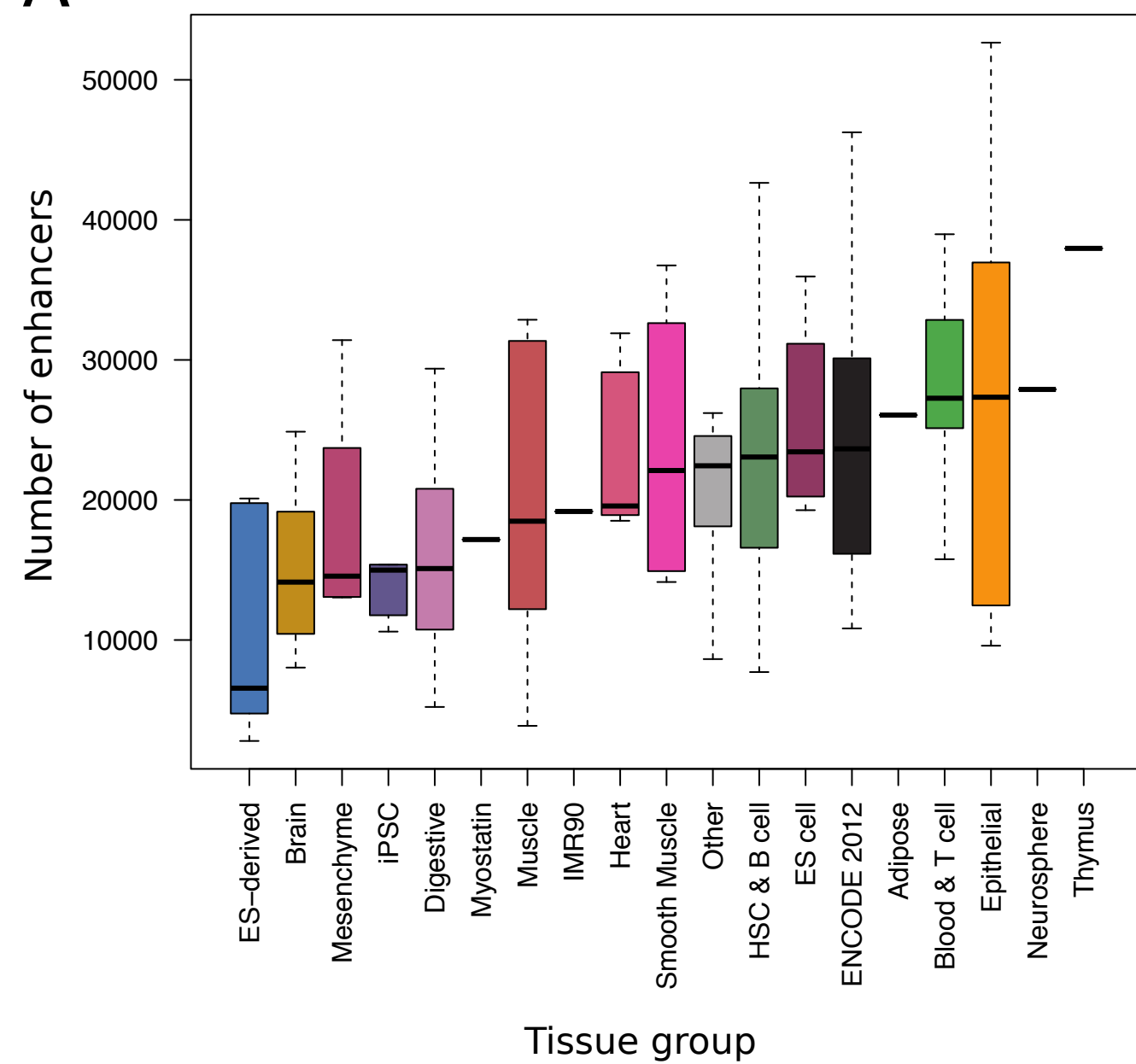**B**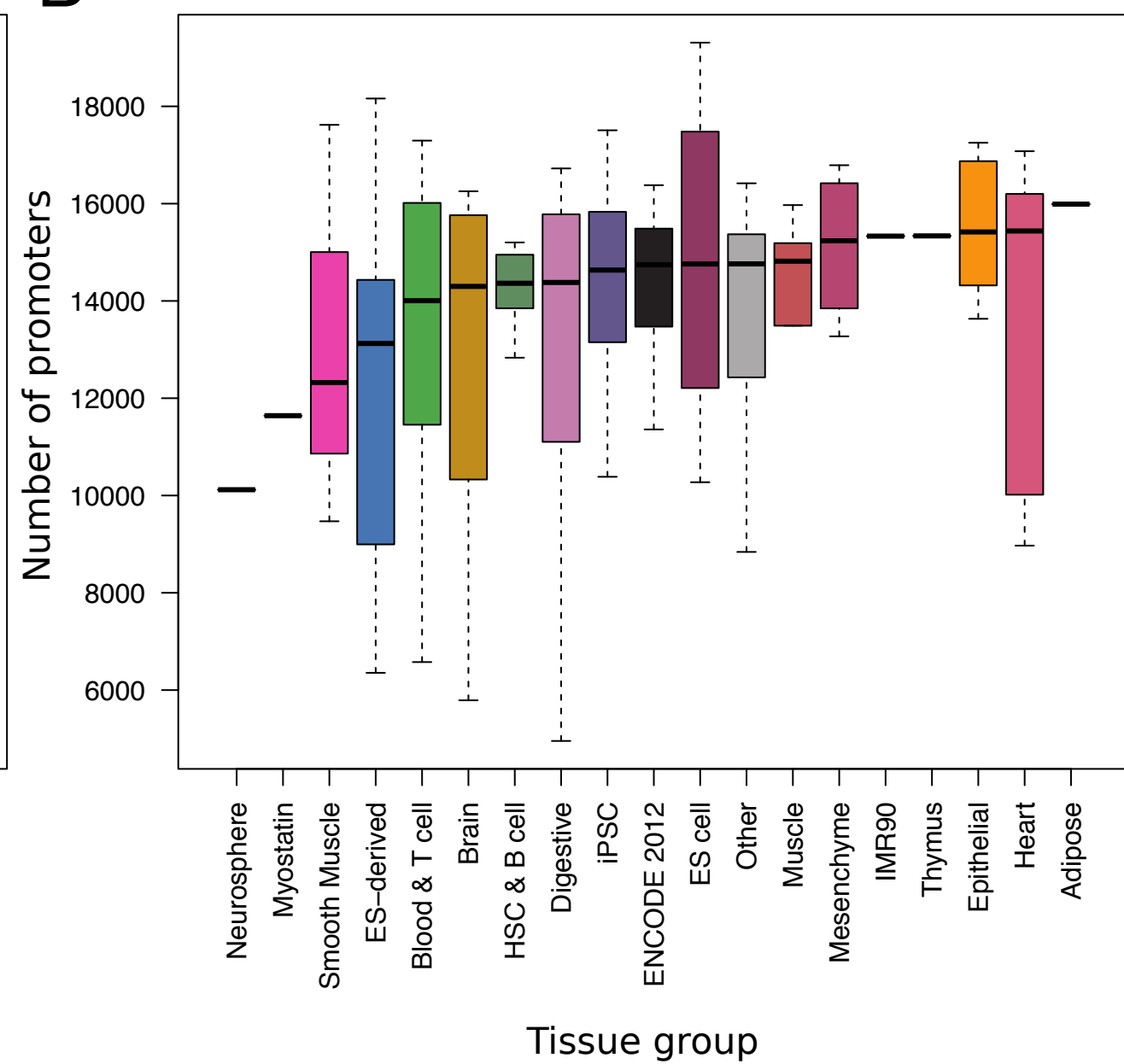**C**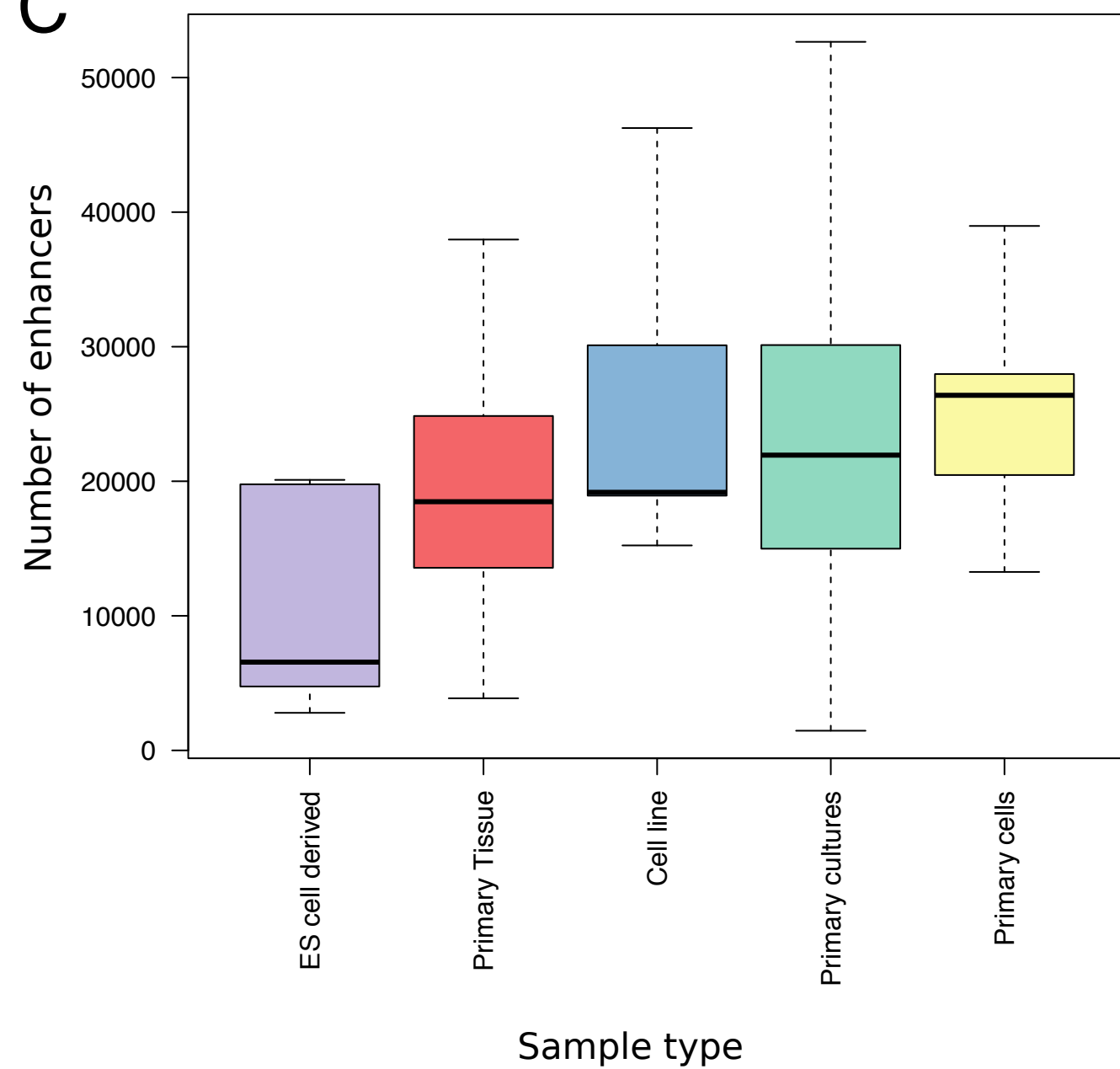**D**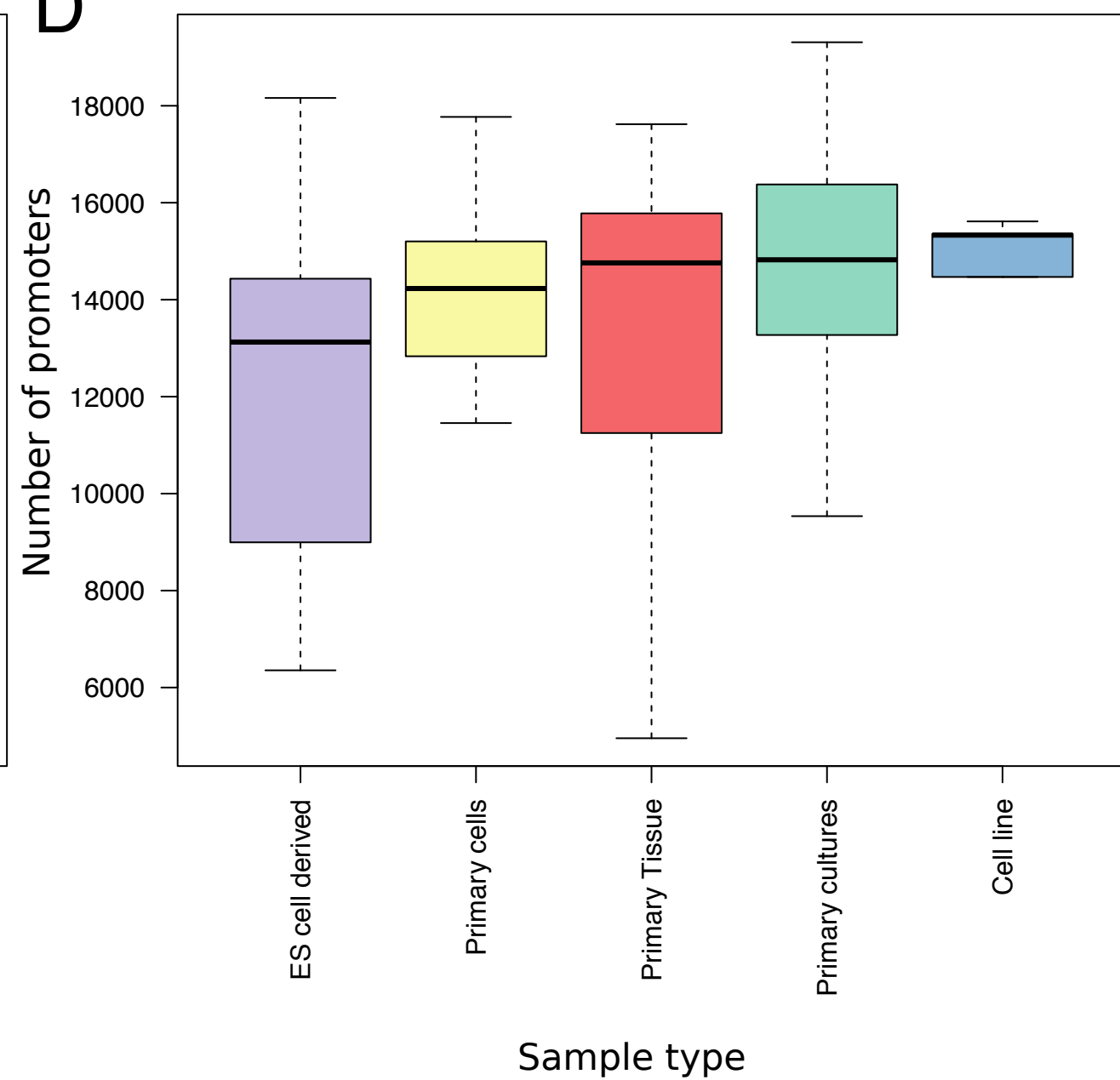

Supplement: S18 Fig — (A) Number of enhancer states per Roadmap Epigenomics cell/tissue group. (B) The same as in (A) for promoters. (C) Number of enhancer states per Roadmap Epigenomics sample type. (D) The same as in (C) for promoters. (PDF) [file pone.0169249.s018.pdf]
