# Supplementary material for: Accurate Promoter and Enhancer Identification in 127 ENCODE and Roadmap Epigenomics Cell Types and Tissues by GenoSTAN
Source: PLoS One. 2017 Jan 5;12(1):e0169249. doi: 10.1371/journal.pone.0169249 (PMC5215863; doi:10.1371/journal.pone.0169249)
Supplement: S3 Fig — (A) Performance of chromatin states in recovering GRO-cap transcription start sites using state segments. Cumulative FDR and recall are calculated using overlap with state segments by subsequently adding states (in order of increasing FDR). (B) The same as in (A) for ENCODE HOT regions. (C) TT-Seq transcribed regions were overlapped with state annotations on bp level and cumulative FDR and recall were calculated. (D,F) Performance of chromatin states in recovering GRO-cap transcription start sites and ENCODE HOT regions using bp overlap. (PDF) [file pone.0169249.s003.pdf]

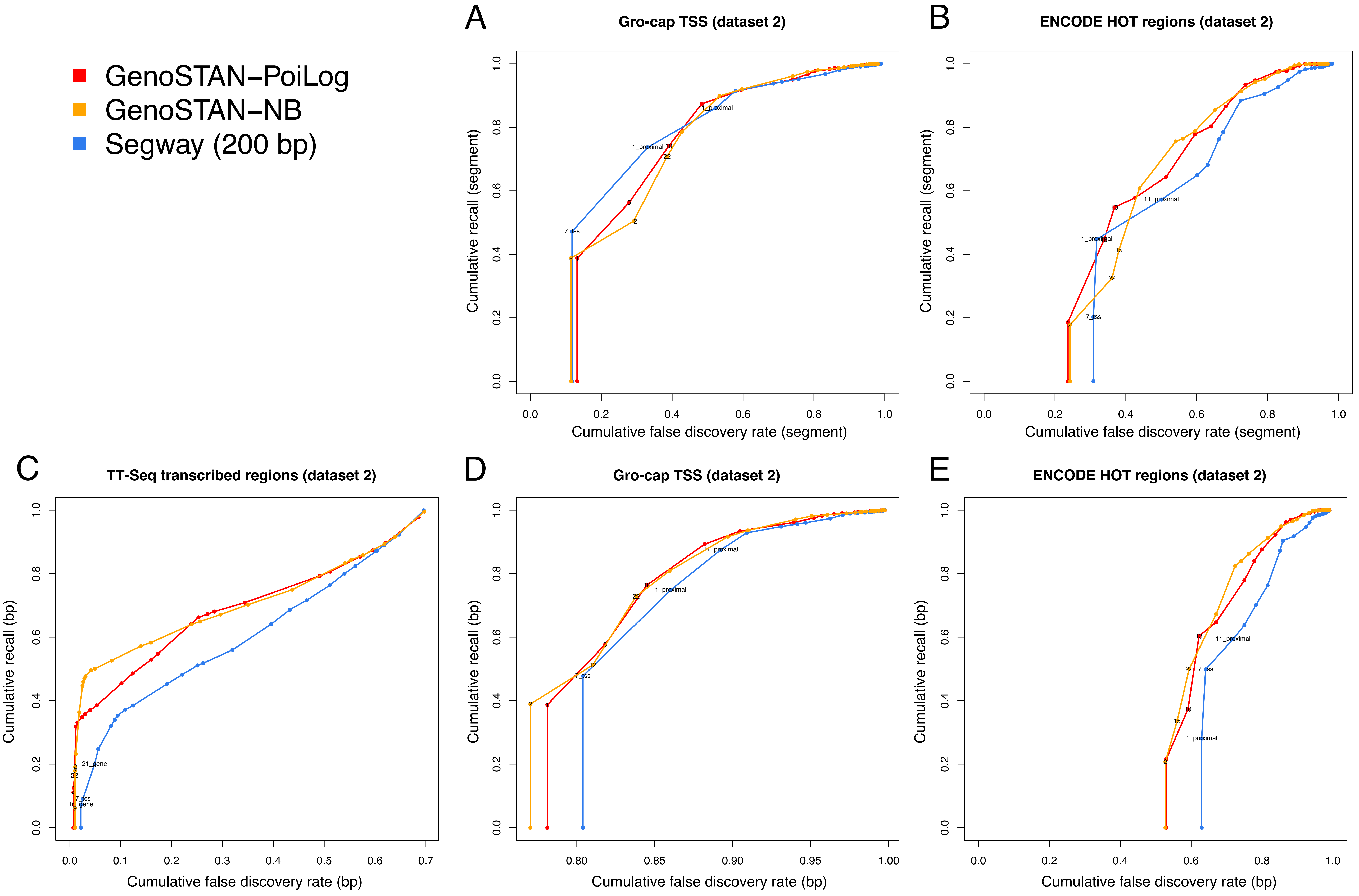

GenoSTAN−PoiLog
GenoSTAN−NB
Segway (200 bp)
A
Gro-cap TSS (dataset 2)
Cumulative recall (segment)
Cumulative false discovery rate (segment)
B
ENCODE HOT regions (dataset 2)
Cumulative recall (segment)
Cumulative false discovery rate (segment)
C
TT-Seq transcribed regions (dataset 2)
Cumulative recall (bp)
Cumulative false discovery rate (bp)
D
Gro-cap TSS (dataset 2)
Cumulative recall (bp)
Cumulative false discovery rate (bp)
E
ENCODE HOT regions (dataset 2)
Cumulative recall (bp)
Cumulative false discovery rate (bp)
