# Supplementary material for: Accurate Promoter and Enhancer Identification in 127 ENCODE and Roadmap Epigenomics Cell Types and Tissues by GenoSTAN
Source: PLoS One. 2017 Jan 5;12(1):e0169249. doi: 10.1371/journal.pone.0169249 (PMC5215863; doi:10.1371/journal.pone.0169249)
Supplement: S5 Fig — (A) Performance of chromatin states in recovering GRO-cap transcription start sites using state segments. Cumulative FDR and recall are calculated using overlap with state segments by subsequently adding states (in order of increasing FDR). (B) The same as in (A) for ENCODE HOT regions. (C) TT-Seq transcribed regions were overlapped with state annotations on bp level and cumulative FDR and recall were calculated. (D,F) Performance of chromatin states in recovering GRO-cap transcription start sites and ENCODE HOT regions using bp overlap. (PDF) [file pone.0169249.s005.pdf]

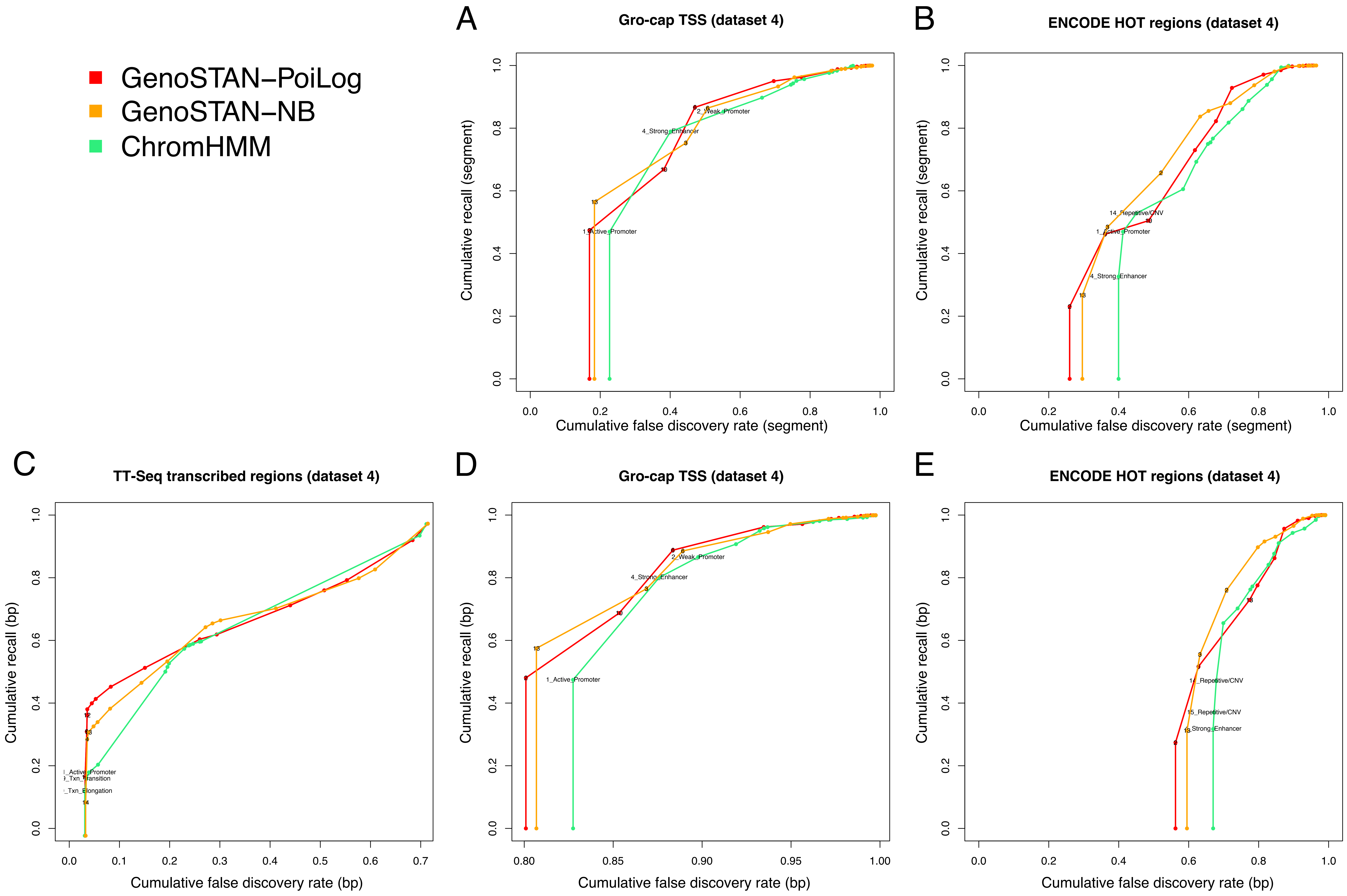
GenoSTAN−PoiLog
GenoSTAN−NB
ChromHMM
A
Gro-cap TSS (dataset 4)
Cumulative recall (segment)
Cumulative false discovery rate (segment)
1_Active_Promoter
2_Weak_Promoter
4_Strong_Enhancer
B
ENCODE HOT regions (dataset 4)
Cumulative recall (segment)
Cumulative false discovery rate (segment)
14_Repetitive/CNV
1_Active_Promoter
4_Strong_Enhancer
C
TT-Seq transcribed regions (dataset 4)
Cumulative recall (bp)
Cumulative false discovery rate (bp)
D
Gro-cap TSS (dataset 4)
Cumulative recall (bp)
Cumulative false discovery rate (bp)
2_Weak_Promoter
4_Strong_Enhancer
1_Active_Promoter
E
ENCODE HOT regions (dataset 4)
Cumulative recall (bp)
Cumulative false discovery rate (bp)
15_Repetitive/CNV
