# Supplementary material for: Accurate Promoter and Enhancer Identification in 127 ENCODE and Roadmap Epigenomics Cell Types and Tissues by GenoSTAN
Source: PLoS One. 2017 Jan 5;12(1):e0169249. doi: 10.1371/journal.pone.0169249 (PMC5215863; doi:10.1371/journal.pone.0169249)
Supplement: S6 Fig — (A) Performance of chromatin states in recovering GRO-cap transcription start sites. Cumulative FDR and recall are calculated by subsequently adding states (in order of increasing FDR). (B) The same as in (A) for ENCODE HOT regions. (C) The fraction of predicted enhancer segments bound by individual TFs is shown for different studies. GenoSTAN enhancers are more frequently bound by TFs than those from other studies. (D) Recall of FANTOM5 promoters and enhancers which are active in K562 (i.e. overlapping with a GRO-cap TSS and an ENCODE DNase hypersensitivity site) by predicted promoters and enhancers is plotted to assess how well models distinguish promoters from enhancers. (E) Predicted enhancers show significantly higher activity than repressed and low coverage regions as measured by a reporter assay (‘*’, ‘**’ and ‘***’ indicate p-values <0.05, 0,01 and 0.001). (F) Comparison of experimental measures of enhancer activity between different studies. (PDF) [file pone.0169249.s006.pdf]

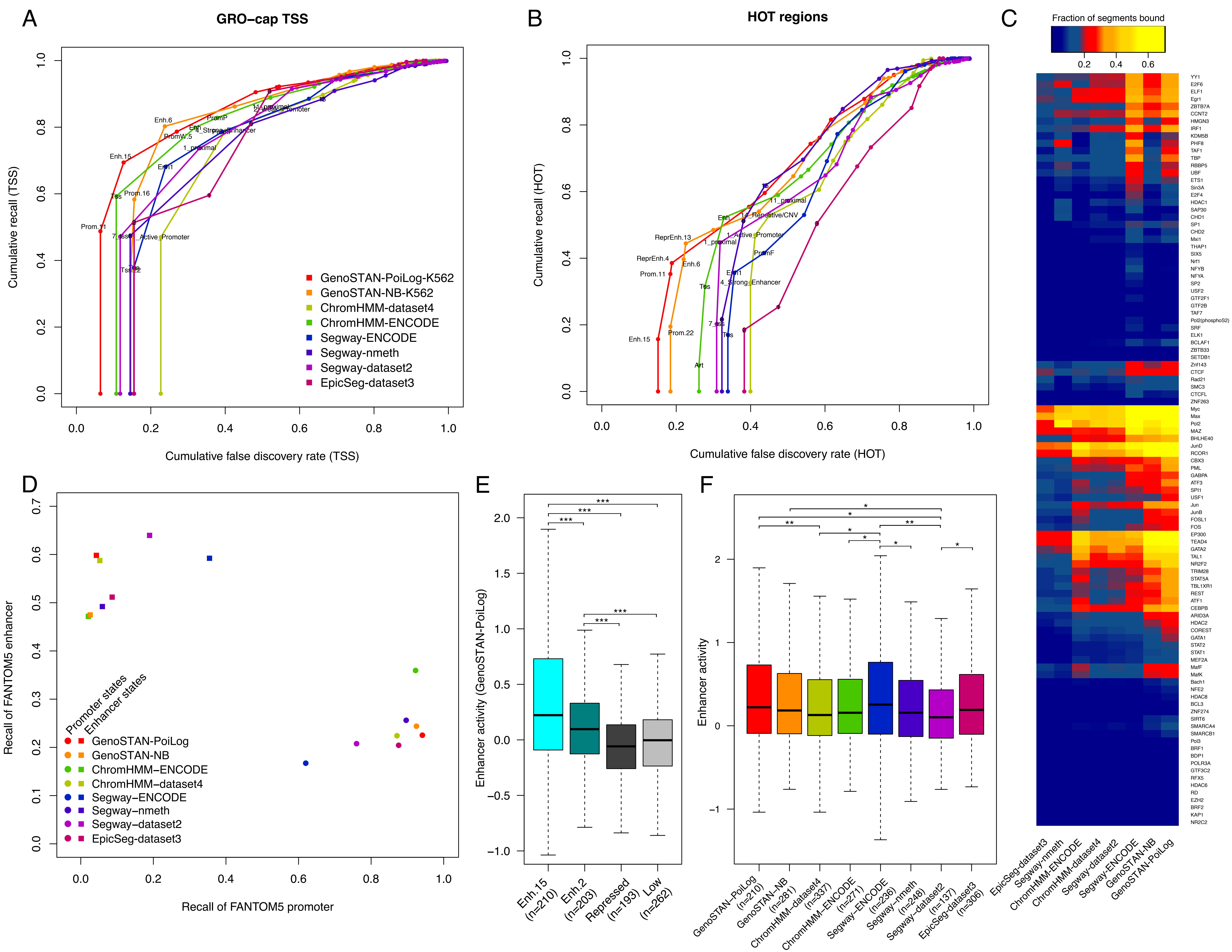

A
GRO−cap TSS
Cumulative recall (TSS)
Cumulative false discovery rate (TSS)
0.0
0.2
0.4
0.6
0.8
1.0
Enh.6
PromP
PromW.5
Enh
4_Strong_Enhancer
Enh.15
1_proximal
Enh1
Tss
Prom.16
Prom.11
7_tss
1_Active_Promoter
Tss.22
GenoSTAN-PoiLog-K562
GenoSTAN-NB-K562
ChromHMM-dataset4
ChromHMM-ENCODE
Segway-ENCODE
Segway-nmeth
Segway-dataset2
EpicSeg-dataset3
B
HOT regions
Cumulative recall (HOT)
Cumulative false discovery rate (HOT)
11_proximal
14_Repetitive/CNV
Enh
ReprEnh.13
1_Active_Promoter
1_proximal
ReprEnh.4
PromF
Enh.6
Prom.11
Enh1
4_Strong_Enhancer
Tss
7_tss
Enh.15
Prom.22
Art
C
Fraction of segments bound
0.2
0.4
0.6
YY1
E2F6
ELF1
Egr1
ZBTB7A
CCNT2
HMGN3
IRF1
KDM5B
PHF8
TAF1
TBP
RBBP5
UBF
ETS1
Sin3A
E2F4
HDAC1
SAP30
CHD1
SP1
CHD2
Mxi1
THAP1
SIX5
Nrf1
NFYB
NFYA
SP2
USF2
GTF2F1
GTF2B
TAF7
Pol2(phosphoS2)
SRF
ELK1
BCLAF1
ZBTB33
SETDB1
Znf143
CTCF
Rad21
SMC3
CTCFL
ZNF263
Myc
Max
Pol2
MAZ
BHLHE40
JunD
RCOR1
CBX3
PML
GABPA
ATF3
SPI1
USF1
Jun
JunB
FOSL1
FOS
EP300
TEAD4
GATA2
TAL1
NR2F2
TRIM28
STAT5A
TBL1XR1
REST
ATF1
CEBPB
ARID3A
HDAC2
COREST
GATA1
STAT2
STAT1
MEF2A
MafF
MafK
Bach1
NFE2
HDAC8
BCL3
ZNF274
SIRT6
SMARCA4
SMARCB1
Pol3
BRF1
BDP1
POLR3A
GTF3C2
RFX5
HDAC6
RD
EZH2
BRF2
KAP1
NR2C2
EpicSeg-dataset3
Segway-nmeth
ChromHMM-ENCODE
ChromHMM-dataset4
Segway-dataset2
Segway-ENCODE
GenoSTAN-NB
GenoSTAN-PoiLog
D
Recall of FANTOM5 enhancer
Recall of FANTOM5 promoter
0.0
0.1
0.2
0.3
0.4
0.5
0.6
0.7
Promoter states
Enhancer states
GenoSTAN−PoiLog
GenoSTAN−NB
ChromHMM−ENCODE
ChromHMM−dataset4
Segway−ENCODE
Segway−nmeth
Segway−dataset2
EpicSeg-dataset3
E
Enhancer activity (GenoSTAN-PoiLog)
−1.0
−0.5
0.0
0.5
1.0
1.5
2.0
***
Enh.15 (n=210)
Enh.2 (n=203)
Repressed (n=193)
Low (n=262)
F
Enhancer activity
−1
0
1
2
*
**
GenoSTAN−PoiLog (n=210)
GenoSTAN−NB (n=281)
ChromHMM-dataset4 (n=337)
ChromHMM−ENCODE (n=271)
Segway−ENCODE (n=236)
Segway−nmeth (n=248)
Segway-dataset2 (n=137)
EpicSeg-dataset3 (n=306)
