# Supplementary material for: Accurate Promoter and Enhancer Identification in 127 ENCODE and Roadmap Epigenomics Cell Types and Tissues by GenoSTAN
Source: PLoS One. 2017 Jan 5;12(1):e0169249. doi: 10.1371/journal.pone.0169249 (PMC5215863; doi:10.1371/journal.pone.0169249)
Supplement: S10 Fig — (A) Median read coverage of GenoSTAN-Poilog-127 chromatin states (left), their number of annotated segments in the genome, their median width and distance to the closest GENCODE TSSs of segments (middle). The right panel shows recall of genomic regions by chromatin states. (B) The same as (A) for GenoSTAN-nb-127. (PDF) [file pone.0169249.s010.pdf]

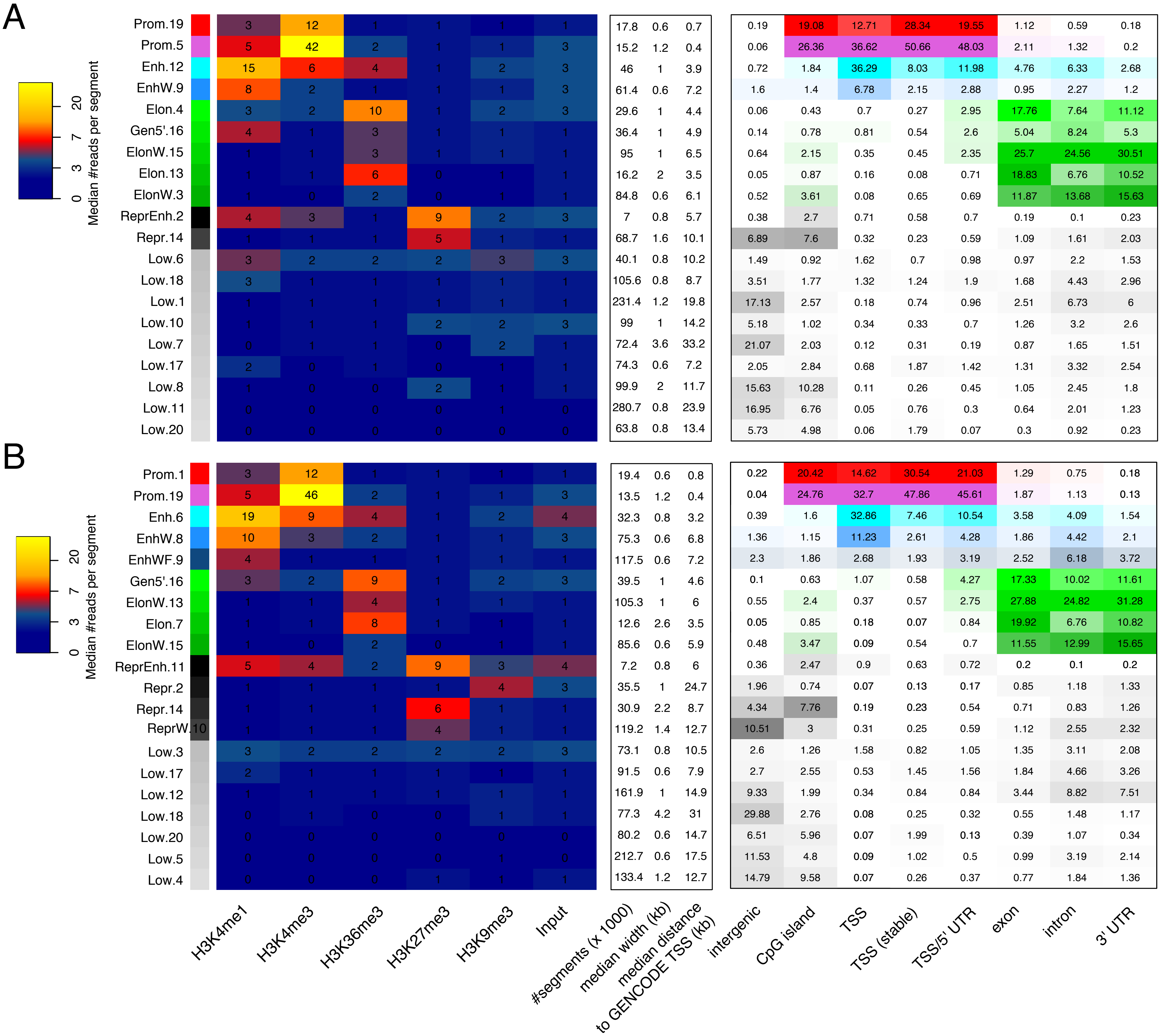

A
Median #reads per segment
0
3
7
20
Prom.19 3 12 1 1 1 1 17.8 0.6 0.7 0.19 19.08 12.71 28.34 19.55 1.12 0.59 0.18
Prom.5 5 42 2 1 1 3 15.2 1.2 0.4 0.06 26.36 36.62 50.66 48.03 2.11 1.32 0.2
Enh.12 15 6 4 1 2 3 46 1 3.9 0.72 1.84 36.29 8.03 11.98 4.76 6.33 2.68
EnhW.9 8 2 1 1 1 3 61.4 0.6 7.2 1.6 1.4 6.78 2.15 2.88 0.95 2.27 1.2
Elon.4 3 2 10 1 2 3 29.6 1 4.4 0.06 0.43 0.7 0.27 2.95 17.76 7.64 11.12
Gen5'.16 4 1 3 1 1 1 36.4 1 4.9 0.14 0.78 0.81 0.54 2.6 5.04 8.24 5.3
ElonW.15 1 1 3 1 1 1 95 1 6.5 0.64 2.15 0.35 0.45 2.35 25.7 24.56 30.51
Elon.13 1 1 6 0 1 1 16.2 2 3.5 0.05 0.87 0.16 0.08 0.71 18.83 6.76 10.52
ElonW.3 1 0 2 0 1 1 84.8 0.6 6.1 0.52 3.61 0.08 0.65 0.69 11.87 13.68 15.63
ReprEnh.2 4 3 1 9 2 3 7 0.8 5.7 0.38 2.7 0.71 0.58 0.7 0.19 0.1 0.23
Repr.14 1 1 1 5 1 1 68.7 1.6 10.1 6.89 7.6 0.32 0.23 0.59 1.09 1.61 2.03
Low.6 3 2 2 2 3 3 40.1 0.8 10.2 1.49 0.92 1.62 0.7 0.98 0.97 2.2 1.53
Low.18 3 1 1 1 1 1 105.6 0.8 8.7 3.51 1.77 1.32 1.24 1.9 1.68 4.43 2.96
Low.1 1 1 1 1 1 1 231.4 1.2 19.8 17.13 2.57 0.18 0.74 0.96 2.51 6.73 6
Low.10 1 1 1 2 2 3 99 1 14.2 5.18 1.02 0.34 0.33 0.7 1.26 3.2 2.6
Low.7 0 1 1 0 2 1 72.4 3.6 33.2 21.07 2.03 0.12 0.31 0.19 0.87 1.65 1.51
Low.17 2 0 1 0 1 1 74.3 0.6 7.2 2.05 2.84 0.68 1.87 1.42 1.31 3.32 2.54
Low.8 1 0 0 2 1 1 99.9 2 11.7 15.63 10.28 0.11 0.26 0.45 1.05 2.45 1.8
Low.11 0 0 0 0 1 0 280.7 0.8 23.9 16.95 6.76 0.05 0.76 0.3 0.64 2.01 1.23
Low.20 0 0 0 0 0 0 63.8 0.8 13.4 5.73 4.98 0.06 1.79 0.07 0.3 0.92 0.23
B
Median #reads per segment
0
3
7
20
Prom.1 3 12 1 1 1 1 19.4 0.6 0.8 0.22 20.42 14.62 30.54 21.03 1.29 0.75 0.18
Prom.19 5 46 2 1 1 3 13.5 1.2 0.4 0.04 24.76 32.7 47.86 45.61 1.87 1.13 0.13
Enh.6 19 9 4 1 2 4 32.3 0.8 3.2 0.39 1.6 32.86 7.46 10.54 3.58 4.09 1.54
EnhW.8 10 3 2 1 1 3 75.3 0.6 6.8 1.36 1.15 11.23 2.61 4.28 1.86 4.42 2.1
EnhWF.9 4 1 1 1 1 1 117.5 0.6 7.2 2.3 1.86 2.68 1.93 3.19 2.52 6.18 3.72
Gen5'.16 3 2 9 1 2 3 39.5 1 4.6 0.1 0.63 1.07 0.58 4.27 17.33 10.02 11.61
ElonW.13 1 1 4 1 1 1 105.3 1 6 0.55 2.4 0.37 0.57 2.75 27.88 24.82 31.28
Elon.7 1 1 8 1 1 1 12.6 2.6 3.5 0.05 0.85 0.18 0.07 0.84 19.92 6.76 10.82
ElonW.15 1 0 2 0 1 1 85.6 0.6 5.9 0.48 3.47 0.09 0.54 0.7 11.55 12.99 15.65
ReprEnh.11 5 4 2 9 3 4 7.2 0.8 6 0.36 2.47 0.9 0.63 0.72 0.2 0.1 0.2
Repr.2 1 1 1 1 4 3 35.5 1 24.7 1.96 0.74 0.07 0.13 0.17 0.85 1.18 1.33
Repr.14 1 1 1 6 1 1 30.9 2.2 8.7 4.34 7.76 0.19 0.23 0.54 0.71 0.83 1.26
ReprW.10 1 1 1 4 1 1 119.2 1.4 12.7 10.51 3 0.31 0.25 0.59 1.12 2.55 2.32
Low.3 3 2 2 2 2 3 73.1 0.8 10.5 2.6 1.26 1.58 0.82 1.05 1.35 3.11 2.08
Low.17 2 1 1 1 1 1 91.5 0.6 7.9 2.7 2.55 0.53 1.45 1.56 1.84 4.66 3.26
Low.12 1 1 1 1 1 1 161.9 1 14.9 9.33 1.99 0.34 0.84 0.84 3.44 8.82 7.51
Low.18 0 1 0 0 1 1 77.3 4.2 31 29.88 2.76 0.08 0.25 0.32 0.55 1.48 1.17
Low.20 0 0 0 0 0 0 80.2 0.6 14.7 6.51 5.96 0.07 1.99 0.13 0.39 1.07 0.34
Low.5 0 0 0 0 1 0 212.7 0.6 17.5 11.53 4.8 0.09 1.02 0.5 0.99 3.19 2.14
Low.4 0 0 0 1 1 1 133.4 1.2 12.7 14.79 9.58 0.07 0.26 0.37 0.77 1.84 1.36
H3K4me1
H3K4me3
H3K36me3
H3K27me3
H3K9me3
Input
#segments (x 1000)
median width (kb)
median distance to GENCODE TSS (kb)
intergenic
CpG island
TSS
TSS (stable)
TSS/5' UTR
exon
intron
3' UTR
