# Supplementary material for: Accurate Promoter and Enhancer Identification in 127 ENCODE and Roadmap Epigenomics Cell Types and Tissues by GenoSTAN
Source: PLoS One. 2017 Jan 5;12(1):e0169249. doi: 10.1371/journal.pone.0169249 (PMC5215863; doi:10.1371/journal.pone.0169249)
Supplement: S15 Fig — (A) The number of traits which are enriched in enhancer states in at least one cell type or tissue is plotted for p-values < 0.05. (B) The same as in (A) but for promoters. (C) The heatmap shows the -log10(p-value) of significantly enriched traits in promoter states (GenoSTAN-Poilog-127, p-value < 0.05, marked by ‘*’). P-values were adjusted for multiple testing using the Benjamini-Yekutieli correction. (PDF) [file pone.0169249.s015.pdf]

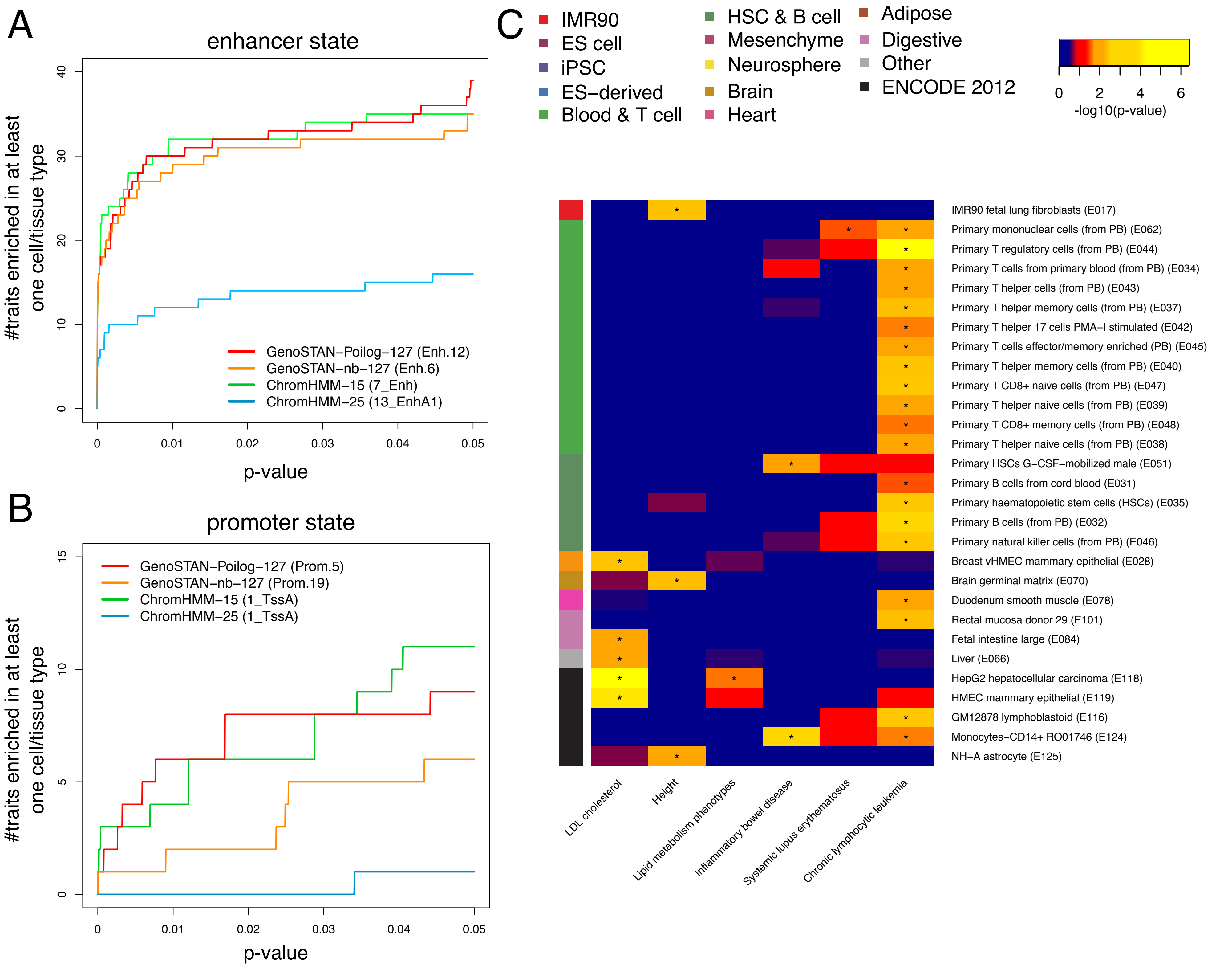
A
enhancer state
#traits enriched in at least one cell/tissue type
p-value
GenoSTAN–Poilog–127 (Enh.12)
GenoSTAN–nb–127 (Enh.6)
ChromHMM–15 (7_Enh)
ChromHMM–25 (13_EnhA1)
B
promoter state
#traits enriched in at least one cell/tissue type
p-value
GenoSTAN–Poilog–127 (Prom.5)
GenoSTAN–nb–127 (Prom.19)
ChromHMM–15 (1_TssA)
ChromHMM–25 (1_TssA)
C
IMR90
ES cell
iPSC
ES–derived
Blood & T cell
HSC & B cell
Mesenchyme
Neurosphere
Brain
Heart
Adipose
Digestive
Other
ENCODE 2012
-log10(p-value)
IMR90 fetal lung fibroblasts (E017)
Primary mononuclear cells (from PB) (E062)
Primary T regulatory cells (from PB) (E044)
Primary T cells from primary blood (from PB) (E034)
Primary T helper cells (from PB) (E043)
Primary T helper memory cells (from PB) (E037)
Primary T helper 17 cells PMA–I stimulated (E042)
Primary T cells effector/memory enriched (PB) (E045)
Primary T helper memory cells (from PB) (E040)
Primary T CD8+ naive cells (from PB) (E047)
Primary T helper naive cells (from PB) (E039)
Primary T CD8+ memory cells (from PB) (E048)
Primary T helper naive cells (from PB) (E038)
Primary HSCs G–CSF–mobilized male (E051)
Primary B cells from cord blood (E031)
Primary haematopoietic stem cells (HSCs) (E035)
Primary B cells (from PB) (E032)
Primary natural killer cells (from PB) (E046)
Breast vHMEC mammary epithelial (E028)
Brain germinal matrix (E070)
Duodenum smooth muscle (E078)
Rectal mucosa donor 29 (E101)
Fetal intestine large (E084)
Liver (E066)
HepG2 hepatocellular carcinoma (E118)
HMEC mammary epithelial (E119)
GM12878 lymphoblastoid (E116)
Monocytes–CD14+ RO01746 (E124)
NH–A astrocyte (E125)
LDL cholesterol
Height
Lipid metabolism phenotypes
Inflammatory bowel disease
Systemic lupus erythematosus
Chronic lymphocytic leukemia
