# Supplementary material for: Accurate Promoter and Enhancer Identification in 127 ENCODE and Roadmap Epigenomics Cell Types and Tissues by GenoSTAN
Source: PLoS One. 2017 Jan 5;12(1):e0169249. doi: 10.1371/journal.pone.0169249 (PMC5215863; doi:10.1371/journal.pone.0169249)
Supplement: S1 Appendix — This document contains the preprocessing steps of dataset 1 for ChromHMM, a detailed description of the GenoSTAN state annotation on dataset 1 and the author contributions. (PDF) [file pone.0169249.s019.pdf]

S1 Appendix

Accurate promoter and enhancer identification in 127 ENCODE and Roadmap Epigenomics cell types and tissues by GenoSTAN

Benedikt Zachert[1,*], Margaux Michel[4], Björn Schwalb[4], Patrick Cramer[4], Achim Tresch[2,3,*], Julien Gagneur[1,¤,*]

1 Feodor-Lynen-Str. 25, Munich, Germany, Gene Center and Department of Biochemistry, Center for Integrated Protein Science CIPSM, Ludwig-Maximilians-Universität München, Germany
2 Zülpicher Str. 47, Cologne, Germany, Department of Biology, University of Cologne, Germany
3 Carl-von-Linne-Weg 10, 24105 Cologne, Germany, Max Planck Institute for Plant Breeding Research, Germany
4 Am Fassberg 11, Göttingen, Germany, Department of Molecular Biology, Max Planck Institute for Biophysical Chemistry, Germany

¤Current Address: Technische Universität München, Department of Informatics, Boltzmannstr. 3, 85748 Garching, Germany
* Correspondence to: Benedikt Zacher, Email: benedikt.zacher@gmail.com and Achim Tresch, Email: tresch@mpipz.mpg.de and Julien Gagneur, Email gagneur@in.tum.de

Contents

1 Pre-processing of dataset 1 for ChromHMM **2**

2 Comparison of GenoSTAN with published chromatin state annotations for the K562 cell line **2**
2.1 Chromatin states recover biologically meaningful features 2
2.2 High variation of enhancer predictions between chromatin state annotations of different studies 3
2.3 Comparison of GenoSTAN with published chromatin state annotations 3

3 Author contributions **5**

1 Pre-processing of dataset 1 for ChromHMM

On dataset 1, without further manual processing of the data, ChromHMM fitted only one transcriptionally active state, which modeled both promoters and enhancers, regardless of state number. We suspected that the high read coverage in the H3K4me1 and H3K4me3 signal tracks made promoters and enhancers indistinguishable after binarization (H3K4me1 and H3K4me3 were called present at both, promoters and enhancers, and they were both called absent elsewhere). When all data tracks were subsampled to the same (and lower) library size, this problem was solved and ChromHMM fitted multiple transcriptionally active states thereby distinguishing promoters from enhancers and, at the same time, increased in accuracy. The same problem occurred for Segway, but changing Segway's parameters did not help distinguish different transcriptionally active chromatin states.

2 Comparison of GenoSTAN with published chromatin state annotations for the K562 cell line

2.1 Chromatin states recover biologically meaningful features

In order to assign biologically meaningful labels to each state of the Poilog-K562 and of the nb-K562 GenoSTAN models (dataset 1), we investigated their read coverage distributions and overlapped the occurrence of a state in the genome with known genomic features. This led to the definition of promoter, enhancer, repressed, actively transcribed and low coverage states, as previously described [1, 2, 3]. The median read coverage in state segments and genomic distributions were very similar for both the Poilog-K562 and the nb-K562 models (Figure 2B, Figure S1). Promoter states were characterized by a low (< 1) H3K4me1/H3K4me3 ratio, in contrast to enhancer states which showed a high ratio (> 1). Further, P300 levels were roughly two-fold higher in the enhancer state, which is in accordance with previous observations [4, 5, 6]. Promoter (Prom) states were located close to annotated GENCODE TSSs [7], with a median distance of 220 bp for GenoSTAN-Poilog-K562 and 400 bp for GenoSTAN-nb-K562 model. Enhancer states (Enh) on the other hand were located further away from TSSs, with a median distance of 3.6 kb (Poilog-K562) (respectively 5.8 kb for nb-K562, Figure 2B, Figure S1). Promoter and enhancer states also differed in their DNA sequence features. 45% of CpG islands were located within promoter states (strong, weak and promoter flanking states) in both models, but only 3% in enhancer states (strong, weak, enhancer flanking states, Figure 2B, Figure S1). While promoter states mostly recovered stable TSSs, enhancer states were located at unstable TSSs (GRO-cap TSSs that are not recovered by the GENCODE annotation), which supports previous findings [8]. Furthermore, both models (GenoSTAN-nb-K562 and GenoSTAN-Poilog-K562) contained 3 states, that we classified as "actively transcribed states", which were characterized by high values of H3K36me3 and overlap with UTRs, introns and exons. Two out of three "transcribed" states were also enriched in promoter associated marks (H3K4me1-3, H3K27ac, H3K9ac) and H4K20me1 and thus represented 5' transitions in transcription. Moreover, both models fitted four repressed states showing high read coverage of H3K27me3. Two of these states also exhibited high DNase-Seq and promoter/enhancer associated histone modification signals, suggesting that these states might reflect repressed regulatory regions (ReprEnh, ReprD). These elements were distal to annotated GENCODE TSSs (median distance: 5.2-11.8 kb). ReprEnh states were also enriched in P300 and recovered 0.2% of CpG islands, while ReprD states had lower P300 levels and recovered 8-9% of CpG islands in the genome (Figure 2B, Figure S1). The remaining states exhibited low coverage in chromatin marks and therefore were labeled as "low" states. Altogether, GenoSTAN accurately recovered many features of known chromatin states and provided a high resolution

map of these in K562.

2.2 High variation of enhancer predictions between chromatin state annotations of different studies

To assess the consistency of promoter and enhancer predictions across studies, we compared the GenoSTAN segmentations to other published segmentations in K562 by ChromHMM ('ChromHMM-ENCODE' [9, 3] and 'ChromHMM-Nature' [2]), Segway ('Segway-ENCODE' [9, 3], 'Segway-nmeth' [10] and 'Segway-Reg.Build' [11]) and EpicSeg [1]. We computed pairwise Jaccard indices (the ratio of the number of common elements over all elements predicted by two methods) of promoter and enhancer states to quantify the agreeement between the predictions of the different studies (Figure S7). Promoter state annotations generally agreed well (median Jaccard-Index: 0.78). However, enhancer prediction varied more (median Jaccard Index: 0.48), suggesting that enhancers are more difficult to annotate. This variation of enhancer calls was also reflected in the different numbers of annotated enhancer segments, which had been shown to vary greatly between different prediction methods [12]. The number of enhancer segments ranged from 10,932 segments in GenoSTAN-Poilog-K562 to 80,043 segments in one Segway annotation [10] (Table S1). Therefore, a thorough assessment of these predictions was necessary to provide a robust and accurate prediction of these elements.

2.3 Comparison of GenoSTAN with published chromatin state annotations

In order to benchmark the different segmentations, we used independent data including evidence of transcriptional activity (GRO-cap TSSs [8]), of transcription factor binding (ENCODE high occupancy target, or HOT regions [13], and ENCODE TF binding sites [9]), and of cis-regulatory activity (enhancer activity assessed by reporter assays [14]), which are all expected to be characteristics of promoters and enhancers. Transcription initiation activity is not only the hallmark of promoters, but also of enhancers [15, 16, 8, 17]. To benchmark the predictions using evidence for transcription, we used published data from a protocol called GRO-cap [8], a nuclear run-on protocol, which very sensitively maps transcription start sites genome-wide. To this end, we sorted for each method chromatin states by their overlap with GRO-cap TSSs by decreasing precision. Starting with the most precise state (i.e. highest overlap with TSSs) we calculated cumulative recall and false discovery rate (FDR) by subsequently adding states with decreasing precision (Figure S6A). GenoSTAN-Poilog-K562 had the highest recall and the lowest FDR (Methods, Figure S6A). GenoSTAN-nb-K562 performed similar to other segmentations (Segway-Reg. Build, ChromHMM-ENCODE). In particular, 94% of GenoSTAN-Poilog-K562 promoters (Prom.11) and 81% of its enhancer regions (Enh.15) overlapped with GRO-cap TSSs. This compares to 85% (Prom.16) and 65% (Enh.6) of GenoSTAN-nb-K562 and 89% (Tss) and 52% (Enh) of ChromHMM-ENCODE promoter and enhancer regions. Interestingly, the two ChromHMM segmentations (ChromHMM-ENCODE [3, 9], ChromHMM-Nature [2]) had very different accuracies for TSSs, which might be due to different data sets (the ChromHMM-ENCODE segmentation had access to DNase data, which is useful to map TSS) or difference in binarization cutoffs and pre-processing. In contrast, the overall accuracy of the Segway annotations was comparable across studies. This comparison shows that GenoSTAN chromatin state annotation identifies putative promoters and enhancers which show transcriptional activity more frequently than previous annotations of K562.

GRO-cap is a very sensitive method that captures also a large amount of TSSs of unstable transcripts. However it is limited to capped RNA species, misses RNAs below the detection threshold and cannot be used to validate regions that are generally bound by TFs but not necessary transcripitonaly active . To address these shortcomings we used two additional

independent features, TF binding and HOT regions. The binding of TFs to a region of DNA is a pre-requisite for potential regulatory function and transcriptional activity. High Occupancy of Target (HOT) regions are genomic regions which are bound by a large number of different transcription-related factors [13], which were shown to function as enhancers [18] and are enriched in disease- and trait-associated genetic variants [19]. As for the benchmark with TSSs, we sorted chromatin states by overlap with HOT regions by decreasing precision and calculated cumulative recall and FDR (Figure S6B). The best performing segmentations for HOT regions were GenoSTAN-Poilog-K562 and GenoSTAN-nb-K562, followed by ChromHMM-ENCODE. The ordering of states with HOT regions was indeed different from the GRO-cap TSSs benchmark. Additionally to GenoSTAN promoter and enhancer states, the repressed enhancer state frequently overlapped with HOT regions with an overall precision of 81% (GenoSTAN-Poilog-K562) and 77% (GenoSTAN-nb-K562). In comparison, the top three ChromHMM-ENCODE states had together a precision of 67%. All other segmentation methods showed a lower precision and recall for HOT regions. This was also reflected in the frequency of individual TF binding sites at enhancer regions, which were generally higher in GenoSTAN enhancer states than in other segmentations (Figure S6C). In particular, only a very small fraction of EpicSeg and Segway-nmeth enhancers were found to be bound by TFs. EpicSeg and Segway-nmeth segmentations were also those with the highest number of predicted enhancers, suggesting that many of these predictions are spurious.

Next, we calculated the recall of FANTOM5 promoters [16] and enhancers [15] to assess how well the models distinguish promoters from enhancers, as it was evident from inspection of specific examples that this distinction was difficult to be established by current methods (Figure 2A). The FANTOM5 consortium have performed extensive mapping of capped transcripts 5' ends using CAGE and defined enhancers and promoters based on transcriptional activity pattern. FANTOM5 enhancers were defined as regions showing balanced bidirectional capped transcripts, a hallmark of enhancer RNAs [15], whereas FANTOM5 promoters were defined as regions where transcription was biased towards one direction. The FANTOM5 annotation of enhancers and promoters could not entirely replace a chromatin state based approach because (i) the use of expression data in FANTOM5 limits the identified regulatory regions to transcriptionally active elements and (ii) CAGE was shown to be not as sensitive to rapidly degraded transcripts as GRO-cap and therefore might miss regulatory enhancers with unstable transcripts [8]. Nonetheless, FANTOM5 provides an annotation of enhancers and promoters based on independent data that is well suited to assess how well the models distinguish promoters from enhancers. We filtered the FANTOM5 annotation to promoters and enhancers for activity in K562 by overlapping them with DHS [9] and GRO-cap TSSs [8]. We considered that a promoter state performed well, when the recall of FANTOM5 promoters was high and the recall of FANTOM5 enhancers was low and vice versa for enhancer states. GenoSTAN-Poilog-K562 and ChromHMM-nature enhancer states recall most FANTOM5 enhancers (60%, Figure S6D, Table S2). For enhancer states, the recall of FANTOM5 promoters was around 10% except for those of Segway-ENCODE, which recalls almost 35% of FANTOM5 promoters and EpicSeg which 21% FANTOM5 promoters. In accordance with this, many promoter regions were erroneously classified as enhancer regions in this segmentation (e.g. TAL1 promoter in Figure 2A). The recall of FANTOM5 enhancers by promoter states was generally higher (17% - 37%). GenoSTAN-Poilog-K562 and -nb-K562 recalled more than 90% of FANTOM5 promoters and around 20% of FANTOM5 enhancers which is comparable to other studies (Segway-nmeth, ChromHMM-nature, EpicSeg). ChromHMM-ENCODE promoter states had a comparable recall of FANTOM5 promoters (92%), but higher recall of FANTOM5 enhancers (37%) (Figure S6D). This strong overlap of ChromHMM-ENCODE promoters with FANTOM5-labeled enhancers is in accordance with our observation that some enhancer regions were errenuously classified as promoters in ChromHMM-ENCODE (Figure 2A). These results show that GenoSTAN segmentations distinguish promoters from enhancers at similar or better accuracy than other segmentations.

So far we only used indirect evidence (TSSs, HOT regions, TF binding, FANTOM5 enhancer) to draw conclusions about the cis-regulatory activity of a candidate enhancer. As additional and direct evidence for the cis-regulatory activity of enhancer regions inferred by GenoSTAN, we overlapped our enhancers to genomic sequences that were previously tested for cis-regulatory activity in a reporter assay, where candidate elements had been cloned into a plasmid upstream of the promoter of a reporter gene [14]. Enhancers from GenoSTAN segmentations showed significantly higher activity than repressed or low coverage regions (GenoSTAN-Poilog-K562 & GenoSTAN-nb-K562: p-value < 0.001 wilcoxon-test, Figure S6E). Interestingly, repressed regions (marked by H3K27me3) showed lower activity than low coverage regions. Moreover, GenoSTAN-Poilog-K562 enhancers showed significantly higher enhancer activity than those of two other studies (Figure S6F). We note that there is a bias against the original study, since the evaluation is made only for regions that had beeen selected by Kheradpour et al. [14]and thus is not a representative set of our predictions. The comparison to all other methods however is justified. This analysis shows that GenoSTAN has higher success rate in predicting *in vivo* enhancer activity than previous methods.

3 Author contributions

BZ, JG and AT developed the statistical methods and computational workflow of the study. BZ developed and implemented all software and scripts and carried out all computational analyses. BS helped with preprocessing of dataset 1. MM and PC helped with interpretation of the biological results. BZ, JG and AT wrote the manuscript with input from all authors. All authors read and approved the final version of the manuscript.

References

[1] Mammana A, Chung HR. Chromatin segmentation based on a probabilistic model for read counts explains a large portion of the epigenome. Genome Biol. 2015;16:151.

[2] Ernst J, Kheradpour P, Mikkelsen TS, Shoresh N, Ward LD, Epstein CB, et al. Mapping and analysis of chromatin state dynamics in nine human cell types. Nature. 2011;473(7345):43–49. doi:10.1038/nature09906.

[3] Hoffman MM, Ernst J, Wilder SP, Kundaje A, Harris RS, Libbrecht M, et al. Integrative annotation of chromatin elements from ENCODE data. Nucleic Acids Res. 2013;41(2):827–841.

[4] Heintzman ND, Stuart RK, Hon G, Fu Y, Ching CW, Hawkins RD, et al. Distinct and predictive chromatin signatures of transcriptional promoters and enhancers in the human genome. Nat Genet. 2007;39(3):311–318.

[5] May D, Blow MJ, Kaplan T, McCulley DJ, Jensen BC, Akiyama JA, et al. Large-scale discovery of enhancers from human heart tissue. Nat Genet. 2012;44(1):89–93.

[6] Visel A, Blow MJ, Li Z, Zhang T, Akiyama JA, Holt A, et al. ChIP-seq accurately predicts tissue-specific activity of enhancers. Nature. 2009;457(7231):854–858. doi:10.1038/nature07730.

[7] Harrow J, Frankish A, Gonzalez JM, Tapanari E, Diekhans M, Kokocinski F, et al. GENCODE: the reference human genome annotation for The ENCODE Project. Genome Res. 2012;22(9):1760–1774.

[8] Core LJ, Martins AL, Danko CG, Waters CT, Siepel A, Lis JT. Analysis of nascent RNA identifies a unified architecture of initiation regions at mammalian promoters and enhancers. Nat Genet. 2014;46(12):1311–1320.

[9] Dunham I, Kundaje A, Aldred SF, Collins PJ, Davis CA, Doyle F, et al. An integrated encyclopedia of DNA elements in the human genome. Nature. 2012;489(7414):57–74.

[10] Hoffman MM, Buske OJ, Wang J, Weng Z, Bilmes JA, Noble WS. Unsupervised pattern discovery in human chromatin structure through genomic segmentation. Nat Methods. 2012;9(5):473–476.

[11] Zerbino DR, Wilder SP, Johnson N, Juettemann T, Flicek PR. The ensembl regulatory build. Genome Biol. 2015;16:56.

[12] Kleftogiannis D, Kalnis P, Bajic VB. DEEP: a general computational framework for predicting enhancers. Nucleic Acids Res. 2015;43(1):e6.

[13] Yip KY, Cheng C, Bhardwaj N, Brown JB, Leng J, Kundaje A, et al. Classification of human genomic regions based on experimentally determined binding sites of more than 100 transcription-related factors. Genome Biol. 2012;13(9):R48.

[14] Kheradpour P, Ernst J, Melnikov A, Rogov P, Wang L, Zhang X, et al. Systematic dissection of regulatory motifs in 2000 predicted human enhancers using a massively parallel reporter assay. Genome Res. 2013;23(5):800–811.

[15] Andersson R, Gebhard C, Miguel-Escalada I, Hoof I, Bornholdt J, Boyd M, et al. An atlas of active enhancers across human cell types and tissues. Nature. 2014;507(7493):455–461.

[16] Forrest AR, Kawaji H, Rehli M, Baillie JK, de Hoon MJ, Haberle V, et al. A promoter-level mammalian expression atlas. Nature. 2014;507(7493):462–470.

[17] Kim TK, Hemberg M, Gray JM, Costa AM, Bear DM, Wu J, et al. Widespread transcription at neuronal activity-regulated enhancers. Nature. 2010;465(7295):182–187. doi:10.1038/nature09033.

[18] Kvon EZ, Stampfel G, Yanez-Cuna JO, Dickson BJ, Stark A. HOT regions function as patterned developmental enhancers and have a distinct cis-regulatory signature. Genes Dev. 2012;26(9):908–913.

[19] Li H, Chen H, Liu F, Ren C, Wang S, Bo X, et al. Functional annotation of HOT regions in the human genome: implications for human disease and cancer. Sci Rep. 2015;5:11633.
