# Supplementary material for: Accurate Promoter and Enhancer Identification in 127 ENCODE and Roadmap Epigenomics Cell Types and Tissues by GenoSTAN
Source: PLoS One. 2017 Jan 5;12(1):e0169249. doi: 10.1371/journal.pone.0169249 (PMC5215863; doi:10.1371/journal.pone.0169249)
Supplement: S1 Table — (PDF) [file pone.0169249.s020.pdf]

	Dataset 1- K562 (one cell type)	
Method/segmentation	#promoters	#enhancers
GenoSTAN-Poilog-K562	11,358 (Prom.11)	10,932 (Enh.15)
GenoSTAN-nb-K562	12,829 (Prom.22)	18,551 (Enh.6)
ChromHMM-Nature	16,118 (1_Active_Promoter)	30,492 (4_Strong_Enhancer)
ChromHMM-ENCODE	16,452 (Tss)	22,323 (Enh)
Segway-ENCODE	19,894 (Tss)	33,518 (Enh1)
Segway-nmeth	25,812 (8)	80,043 (0)
Segway-Reg.Build	13,668 (7_tss)	38,992 (11_proximal)
EpicSeg	16,192 (2)	53,982 (3)

	ENCODE and Roadmap epigenomics - 127 cell types and tissues	
Method/segmentation	#promoters	#enhancers
GenoSTAN-Poilog-127	15,229 (Prom.5)	45,955 (Enh.12)
GenoSTAN-nb-127	13,547 (Prom.19)	32,280 (Enh.6)
GenoSTAN-Poilog-20	12,710 (Prom.15)	19,730 (Enh.9)
GenoSTAN-nb-20	14,168 (Prom.14)	15,655 (Enh.9)
ChromHMM-15	21,002 (1_TssA)	92,824 (7_Enh)
ChromHMM-18	20,049 (1_TssA)	22,678 (9_EnhA1)
ChromHMM-25	12,525 (1_TssA)	12,706 (13_EnhA1)
