# Supplementary material for: Accurate Promoter and Enhancer Identification in 127 ENCODE and Roadmap Epigenomics Cell Types and Tissues by GenoSTAN
Source: PLoS One. 2017 Jan 5;12(1):e0169249. doi: 10.1371/journal.pone.0169249 (PMC5215863; doi:10.1371/journal.pone.0169249)
Supplement: S2 Table — Two promoter and enhancer states were used for each segmentation, except for the EpicSeg segmentation, which only fitted one enhancer state. (PDF) [file pone.0169249.s021.pdf]

	**Dataset 1- K562 (one cell type)**	
Method/segmentation	promoter states	enhancer states
GenoSTAN-Poilog-K562	Prom.11, PromW.5	Enh.15, Enh.2
GenoSTAN-nb-K562	Prom.16, Prom.22	Enh.6, Enh.19
ChromHMM-Nature	Tss, TssF	Enh, EnhW
ChromHMM-ENCODE	1_Active_Promoter, 2_Weak_Promoter	4_Strong_Enhancer, 5_Strong_Enhancer
Segway-ENCODE	Tss, PromF	Enh1, Enh2
Segway-nmeth	8,6	0, 13
Segway-Reg.Build	7_tss, 0_proximal	1_proximal, 11_proximal
EpicSeg	2	3

	**ENCODE and Roadmap epigenomics - 127 cell types and tissues**	
Method/segmentation	promoter states	enhancer states
GenoSTAN-Poilog-127	Prom.19, Prom.5	Enh.12, EnhW.9
GenoSTAN-nb-127	Prom.1, Prom.19	Enh.6, EnhW.8
GenoSTAN-Poilog-20	Prom.15, Prom.6	Enh.9, EnhF.13
GenoSTAN-nb-20	Prom.14, Prom.21	Enh.9, EnhF.12
ChromHMM-15	1_TssA, 2_PromU	13_EnhA1, 14_EnhA2
ChromHMM-18	1_TssA, 2_TssFlnk	9_EnhA1, 10_EnhA2
ChromHMM-25	1_TssA, 2_TssAFlnk	7_Enh, 6_EnhG
